# Stem cell therapy for female stress urinary incontinence: Results, limitations and lessons learned from a pilot clinical study

**DOI:** 10.1371/journal.pone.0342452

**Published:** 2026-02-27

**Authors:** Rodrigo C. Souza, Maria A. T. Bortolini, Yasmin Melino, Juliana A. P. de Godoy, Kelen Alvarez, Denise De Oliveira, Andrea T. Kondo, Jose M. Kutner, Mariane Secco, Eder Zucconi, Natássia Vieira, Sérgio Podgaec, Rebecca S. P. Silva, Rodrigo A. Castro

**Affiliations:** 1 Paulista School of Medicine, Division of Urogynecology and Reconstructive Pelvic Surgery, Department of Gynecology, Federal University of São Paulo, São Paulo, São Paulo, Brazil; 2 Department of Gynecology, Federal Hospital of Ipanema, Rio de Janeiro, Brazil; 3 Hospital Israelita Albert Einstein, São Paulo, São Paulo, Brazil; 4 StemCorp, Ltda., São Paulo, São Paulo, Brazil; 5 Department of Obstetrics and Gynecology, Medical School, University of São Paulo, São Paulo, Brazil; ICAR-Indian Veterinary Research Institute: Indian Veterinary Research Institute, INDIA

## Abstract

**Objective:**

This pilot clinical study aimed to develop and validate standardized manufacturing and quality control procedures for autologous skeletal muscle-derived (SkM-MSCs) and bone marrow-derived mesenchymal stem cells (BM-MSCs), and to explore the feasibility, safety and preliminary efficacy of periurethral injection of these products in women with stress urinary incontinence (SUI).

**Methods:**

Twenty-six diagnosed with SUI were enrolled and allocated to receive either SkM-MSCs or BM-MSCs. Autologous MSCs were isolated from skeletal muscle or bone marrow biopsies, expanded under Good Manufacturing Practices (GMP), and subjected to rigorous quality control assessments, including identity, genetic stability, viability, potency, and sterility. In this pilot study, ten million MSCs were injected periurethrally under local anesthesia, and participants were followed for 12 months post-treatment.

**Results:**

Eleven SkM-MSCs and nine BM-MSCs final products met all quality criteria and were administered. One participant from the SkM-MSCs lost the follow-up. MSC therapies were well tolerated, with no long-term adverse effects or tumor formation observed. In the SkM-MSCs group, the proportion of women with a positive cough test decreased significantly from 100% to 40% (p = 0.010). In the BM-MSCs group, modest improvements were seen but did not reach statistical significance. Overall, improvements in both pad test outcomes and quality of life measures among participants were observed, though not uniformly significant. The study was discontinued before reaching its intended sample size due to limited efficacy, logistical challenges, and financial constraints.

**Conclusion:**

Autologous MSC‑based therapy for SUI was feasible and showed an acceptable short‑term safety profile in this pilot research setting; however, clinical efficacy remained modest. The manufacturing and quality control methodology is reproducible in specialized cell processing centers, but its application should remain confined to clinical research conducted in compliance with current regulations governing human cell therapy. Future studies with optimized cell products, refined delivery strategies and adequately powered, randomized designs are required before any potential translation to routine clinical practice can be considered.

## Introduction

Stress urinary incontinence (SUI), defined as the involuntary leakage of urine during physical exertion or activities that increase intra-abdominal pressure, such as coughing or sneezing [1], is a common and distressing condition among women, significantly affecting quality of life. Its etiology is multifactorial, often related to urethral hypermobility and/or intrinsic sphincter deficiency, typically as a result of childbirth, aging, or pelvic floor trauma [2,3].

Conventional treatments for SUI include lifestyle modifications, pelvic floor muscle training, pharmacologic agents, and surgical interventions, particularly midurethral sling procedures using synthetic prosthesis [3]. While surgical treatments often performed with good results they are not without complications such as voiding dysfunction, pain, mesh erosion, and failure rates over time have been increasingly reported [3]. Additionally, a subset of women may not be ideal surgical candidates due to comorbidities, previous failed surgeries, or patient preference for less invasive options.

Given these challenges, stem cells (SCs) therapy has emerged as a potential treatment for SUI due to their capability of self-renewal and ability to differentiate into mature cell types [4,5]. The local injection of SCs into the periurethral region has been explored as a minimally invasive and safe treatment option, primarily for regenerating the external urethral sphincter and potentially restoring other periurethral components including smooth muscle, connective tissue, vasculature and nerves [6–12].

Adult stem cells (ASCs) found in various organs play a crucial role in cellular regeneration during both physiological and pathological processes [13]. It is known that some types of adult stem cells (such as mesenchymal stem cells – MSC) have greater plasticity and may therefore represent promising candidates for cell therapeutic applications [13].

Although the application of bone marrow-derived mesenchymal stem cells (BM-MSCs) in treating SUI remains inadequately documented, skeletal muscle-derived mesenchymal stem cells (SkM-MSCs) have been extensively studied with contradictory findings [14–16]. Most experimental and clinical investigations involving muscle-derived cells lack comprehensive methodologies for cellular product preparation, including cells isolation, identification, characterization, and thorough safety assessments prior to clinical use as preconized by the International Society for Cellular Therapy (ISCT) [17]. The unclear disclosure and inconsistent methodologies prevent a full understanding of the mechanisms of action of these cellular products, such as their renewal and differentiation potential. This hinders comparative analyses across studies and affects reproducibility [18,19].

The development and use of cell‑based medicinal products are now increasingly regulated under specific human cell therapy and advanced therapy medicinal product frameworks. Within this context, the present study was conceived as a pilot clinical research initiative primarily focused on establishing and validating manufacturing and quality control procedures for autologous MSC products for SUI, rather than defining a definitive treatment protocol for routine clinical practice.

We hypothesized that optimized autologous biological products composed of undifferentiated MSCs derived from adult tissues (skeletal muscle and bone marrow) can be safely developed and manufactured for clinical research use in women with SUI, in accordance with applicable quality standards for human cell therapy. Our objectives include the establishment and validation of protocols for isolation and characterization of SkM-MSCs and BM-MSCs products and the exploratory evaluation of the feasibility, safety and preliminary efficacy of their periurethral administration. Moreover, this study aims to evaluate the feasibility, efficacy, and safety profile of autologous SkM-MSCs and BM-MSCs administered via periurethral injection in women with SUI, thereby addressing existing challenges and enhancing knowledge in cellular therapies for SUI management.

## Methods

### Study design

This pilot, multicenter, non-randomized and prospective clinical study was conducted under the auspices of the Urogynecology and Vaginal Surgery Section of the General Gynecology Division at the Federal University of São Paulo – Paulista School of Medicine (UNIFESP-EPM) and involved women with diagnosis of SUI. The date of the first enrollment of the patients was Jan 1^st^, 2018, and Dec 31, 2020, as the last enrollment date. The last patient enrolled completed the follow-up on Feb 01, 2022. The study involved collaborative efforts from São Paulo Hospital (UNIFESP), the Gynecologic Surgery Section at Hospital Santa Marcelina Hospital, the Hemotherapy and Cell Therapy Department at Hospital Israelita Albert Einstein, and StemCorp – Specialized Center in Stem Cells. The group allocation followed the sequence of the cell products development: the first group included those women with SUI treated with SkM-MSCs, followed by the second group treated with BM-MSCs.

This study was approved by the Research Ethics Committee (REC) of all participating institutions. The following approvals were obtained: Opinion No. 707.060 (Certificate of Presentation for Ethical Consideration – CAAE: 18150613.7.1001.5505), Opinion No. 3.907.497 (CAAE: 18150613.7.3005.0071), and Opinion No. 4.400.583 (CAAE: 18150613.7.3002.0066). All participants provided written informed consent prior to enrollment. The authors confirm that this trial for this intervention is registered on the Brazilian Clinical Trials Registry (ReBec), Register Number: RBR-29x2pr, Trial identification UTN code: U1111-1230–8247. This report complies with the guidelines of the TREND Reporting Guidelines for Nonrandomized/Quasi-Experimental Study Designs [20].

The authors disclose that the original clinical trial protocol was modified during the research due to: development of the protocols to obtain the cell products, due to logistical challenges, patient recruitment and follow-up issues, resource limitations, and the pandemic. Those modifications were properly notified to and approved by the Research Ethics Committees.

This study was designed as an exploratory pilot clinical investigation intended to inform future, more definitive trials of MSC-based therapies for SUI, rather than to establish a protocol for routine clinical use.

### Protocol deviations between the initial and updated study designs

The initial study protocol outlined a prospective randomized clinical trial involving 45 patients diagnosed with stress urinary incontinence (SUI), who were to receive periurethral injections of 5 ml solution containing 100 million autologous stem cells derived from muscle, bone marrow, or adipose tissue. The design included three intervention groups corresponding to the different cell sources SkM-MSCs, BM-MSCs or adipose derived-stem cells.

During the study, adaptations led to deviations from the initial protocol, resulting in the updated protocol with important modifications as outlined in the methods section of this manuscript. Overall, the study design was a non-randomized trial, with two intervention groups: SkM-MSCs and BM-MSCs. The sample size was reduced from the original 45 patients to a total of 26 patients, and the cellular dose administered was 10 million stem cells per patient as opposed to the anticipated 100 million cells. Modifications in the protocol for obtaining the stem cells to use in the therapy of women with SUI was foresaw by the researchers as the primary aim was to develop optimized autologous cellular products composed of undifferentiated stem cells to be used in women with SUI.

Despite the modifications, the aims of developing the cell product and the evaluating the safety and efficacy of autologous stem cell therapy for SUI was maintained.

### Participants

Patients were enrolled at the outpatient clinic of the Urogynecology and Vaginal Surgery Section – UNIFESP-EPM, and the Gynecologic Surgery Section at Hospital Santa Marcelina, and were selected based on their medical history and gynecological examination. The cell therapy injection and the follow-up assessments were conducted at the same unit. Inclusion criteria encompassed a diagnosis of SUI or mixed urinary incontinence with predominant stress component, with or without prior conservative treatment. Participants had to exhibit urinary leakage as confirmed by either the cough stress test [21] or the simplified pad test [22,23]. Exclusion criteria included age under 18, pelvic organ prolapse exceeding the hymen level, previous surgeries for SUI, anatomical conditions compromising periurethral injection, prior pelvic radiotherapy, paradoxical incontinence, neurogenic bladder, urinary obstruction, refusal to participate, and inability to comprehend the study. Eligible patients were informed about the risks and benefits of the experimental treatment, provided written informed consent, and they were non-randomly assigned to one of two groups: skeletal muscle-derived cell (SkM-MSCs) therapy or bone marrow-derived cell (BM-MSCs) therapy. Neither the investigators or the patients were blinded to the intervention allocation.

To address potential sources of bias associated with the non-randomized design, several methodological strategies were employed. Group assignment followed the chronological order of cellular product development (SkM-MSCs followed by BM-MSCs), minimizing investigator-driven allocation decisions. Eligibility criteria were strictly defined and uniformly applied to all participants, ensuring comparability between groups. All outcome measures were assessed using standardized protocols by trained evaluators blinded to group allocation, thus reducing measurement bias. Objective and a validated assessment tool (stress test and the 20-minute pad test) were prioritized to minimize subjective interpretation. Baseline characteristics were analyzed to identify and account for any imbalances between groups. Furthermore, all patients followed the same schedule of clinical assessments at predefined time points (2 weeks, 3-, 6-, and 12-months post-treatment), ensuring consistency in follow-up procedures across groups. Together, these measures were implemented to mitigate potential biases and strengthen the internal validity of the study.

### Outcomes and measurements

#### Primary outcomes.

To develop optimized autologous cellular products composed of undifferentiated SkM-MSCs and BM-MSCs properly validated through quality control tests in accordance with national and international standards, for safe administration in pilot clinical research setting in women with SUI.

The quality control tests include identity, genetic stability, viability and cell counting, potency, endotoxin detection, and microbiological testing.

#### Secondary outcomes.

To evaluate the feasibility of periurethral injection of autologous SkM-MSCs and BM-MSCs for the treatment of both groups of women with SUI. To determine the efficacy and safety of the cell therapy over a 12-month period.

We assessed the clinical findings using a cough test, pad test, and quality of life questionnaire.

#### Assessment.

##### Baseline:

Stress urinary incontinence was evaluated through a physical examination incorporating the cough stress test and Valsalva maneuver performance, both with the bladder comfortably filled after the patients consumed 500 mL of water. In a semi-seated position, patients were instructed to cough on command while the examiner observed for any synchronous urine loss. A positive result was defined by the presence of at least one episode of leakage, whereas a negative result required four or more coughs without leakage [21]. Additionally, a simplified pad test was performed. The bladder was emptied via catheter, and 250 mL of distilled water was instilled. A pre-weighed pad was placed at the perineum, and the patient engaged in various activities including coughing, squatting, stair climbing, jumping, walking, and handwashing over a 20-minute period. A weight increase of ≥2 g was considered a positive result [22,23]. Baseline quality of life was assessed using the I-QoL questionnaire, validated in Portuguese, administered immediately after the initial consultation [24].

##### Follow-up:

Patients were followed up at 2 weeks post-biopsy and 2 weeks post-therapy, which involved clinical examinations mainly addressing adverse effects of the procedures. Moreover, assessments were performed at 3-, 6-, and 12-month post-treatment. The 3- and 6-months assessments involved a targeted medical history and physical examination to evaluate symptom relief, side effects, and potential local tumor formation. At the 12-month follow-up, additional evaluations including cough stress test, the 20-minute pad test, and the I-QoL questionnaire were repeated to comprehensively assess treatment outcomes. The participants were contacted by telephone prior to their appointment to enhance their adherence. The participants had direct contact with the investigators throughout the study.

### Development of stem cells products: Protocols

#### SkM-MSCs: Biopsy procedure, MSCs isolation and manufacturing.

Muscle biopsies were performed in outpatient setting at São Paulo Federal Hospital or at Santa Marcelina Hospital, under aseptic conditions. Local anesthesia was administered using 2% lidocaine hydrochloride (5–10 mL). A 3.0-cm incision was made in the quadriceps femoris muscle to extract a 1.0 x 1.0 cm fragment. The *fascia lata* was sutured with 3−0 polyglycolic acid, and the skin incision was closed with 4−0 nylon sutures, which were removed 15 days post-biopsy.

The extracted muscle fragments were stored in sterile vials containing phosphate-buffered saline (PBS) at pH 7.4, supplemented with 2% of antibiotic–antimycotic solution (10,000 units/mL of penicillin, 10,000 ug/mL of streptomycin and 25ug/mL of Amphotericin B; Gibco). Samples were transported in a UN3373-compliant box with recyclable ice and temperature logger (Kooltrak) to maintain a temperature range of 4-24^o^C. The samples arrived at the GMP-certified StemCorp Cell Processing Center on the same day, and processing commenced within 24 hours.

Standard operational protocol for processing and expansion of the SkM-MSCs is presented as supplement (S1 File). Cell isolation was conducted in a Class II laminar flow hood. Muscle fragments were washed twice in PBS (Gibco) containing 0.05% gentamicin (Gibco) and 1% antibiotic-antimycotic solution (Gibco). After washing, the fragments were mechanically fragmented and placed in a 50 mL tube (Sarstedt) containing a 0.075% collagenase solution (Sigma) prepared in PBS with calcium (Ca^2^⁺) and magnesium (Mg^2^⁺) ions (Sigma). This tube was sealed with Parafilm and incubated in a water bath at 37°C for 30 minutes, with agitation every 10 minutes using a vortex mixer. Following enzymatic digestion, PBS was added to halt the reaction, and undigested tissue fragments were removed. The mixture was centrifuged at 280 × g for 5 minutes at room temperature. The resulting pellet was resuspended in culture medium composed of DMEM F12 (Gibco), supplemented with 15% USDA-tested fetal bovine serum (FBS; HyClone), 1% antibiotic-antimycotic solution (Gibco), and 1% non-essential amino acids (Sigma).

Cell counting was performed using a Neubauer chamber. After counting, cells were seeded into a 6-well plate (Corning) and incubated in a humidified atmosphere at 37°C with 5% CO₂ (Thermo Fisher). The culture medium was refreshed every 3–4 days until the cells reached approximately 80–90% confluence.

**Fig 1:**
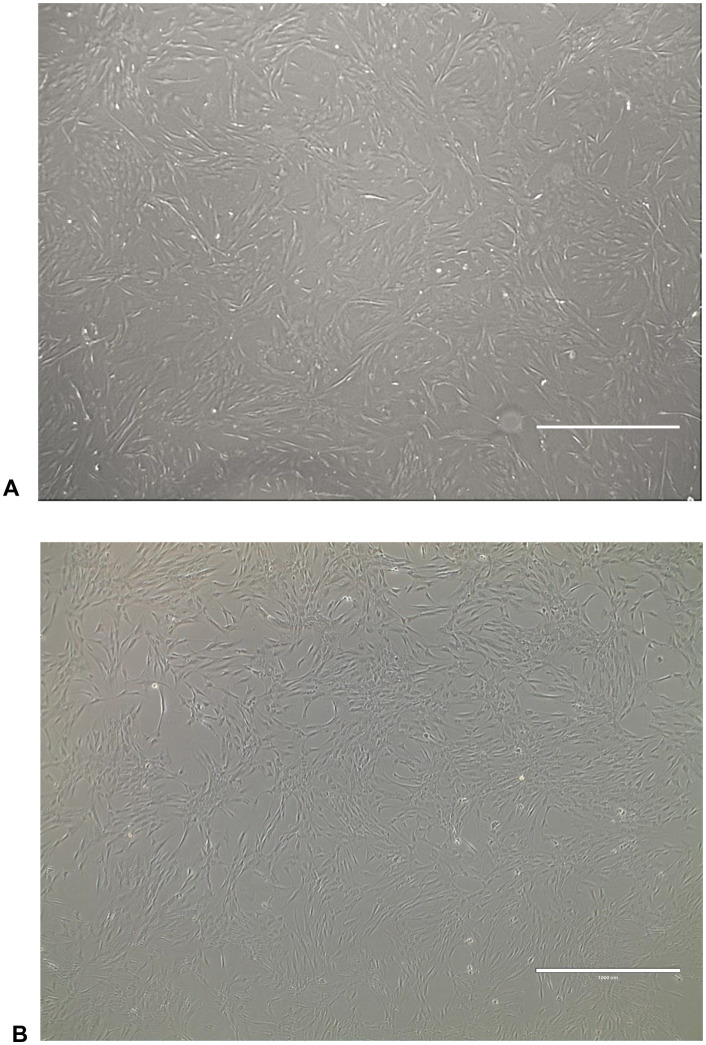
MSCs culture images. **(A)** In vitro culture of SkM-MSCs; **(B)** In vitro culture of BM-MSCs. Both cultures are shown at passage 4 (P4), displaying the characteristic fibroblast-like morphology of MSCs and reaching confluence suitable for clinical application. Images were captured at 40X magnification. Scale bar represents 1000 μm.

Cells were trypsinized using TrypLE Express (Gibco) as follows: the culture medium was removed, cells were washed with PBS (Gibco), and TrypLE Express was added to detach cells from the plate surface. The plate was incubated at 37°C with 5% CO₂ for approximately 5–8 minutes until detachment occurred. FBS-containing culture medium was then added to neutralize enzymatic activity. Detached cells were collected and transferred to progressively larger flasks for expansion until reaching 80–90% confluence: Passage 1 (P1) cells were transferred to a 25 cm^2^ flask (Corning); Passage 2 (P2) cells were transferred to a 75 cm^2^ flask; Passage 3 (P3) cells were transferred to a 225 cm^2^ flasks; Passage 4 (P4) cells were further expanded into three new flasks of 225 cm^2^ size; Passage 5 (P5) cells were cultured in nine 225 cm^2^ flasks until reaching 80–90% confluence. During the final passage, autologous serum replaced FBS to eliminate residual animal proteins from the final product (Fig 1).

For each patient, ten million cells (1 x 10^7^) were suspended in sterile PBS for periurethral injection. A small aliquot of cells was reserved for viability and quality control tests.

The final product was packaged in a sealed sterile syringe (20 mL) compliant with GMP standards. Upon delivery to UNIFESP-EPM outpatient clinic, cell viability and transport temperature conditions were verified to ensure compliance with specified requirements.

#### BM-MSCs: Sample acquisition, MSCs isolation and manufacturing.

Bone marrow harvesting was performed in an outpatient setting by the medical team of the Department of Hemotherapy and Cell Therapy at Hospital Israelita Albert Einstein. Standard operational protocol for processing and expansion of the BM-MSCs is presented as supplement (S2 File). Patients received intravenous and inhalation sedation, along with local anesthesia (2% lidocaine) administered at the superior iliac crest. A bone marrow biopsy was performed using an 11G biopsy needle connected to a 20 mL syringe containing an anticoagulant. Approximately 50 mL of bone marrow was collected and transported in a sterile bag to the GMP-compliant laboratory within the same facility.

The bone marrow aspirated was diluted in PBS, filtered, and mixed with Ficoll-Paque Plus density gradient medium (GE Healthcare). The mixture was centrifuged at 500 × g for 30 minutes at 21°C to isolate the mononuclear cell fraction. The isolated mononuclear cells were washed and centrifuged again at 500 × g for 10 minutes at 21°C. Cell counting was performed using a Neubauer chamber.

For initial culture, passage 0 (P0), cells were seeded in two T75 flasks (Corning) at a density of 5–6 × 10³ cells/cm², resulting in 3.75–4.5 × 10⁵ cells per T75 flask or 1.0 × 10⁷ cells in a T175 flask (Passage P0). MSCs were cultivated in low-glucose DMEM (DMEM-LG; Gibco) supplemented with 1% antibiotic–antimycotic solution (10,000 units/mL of penicillin, 10,000 μg/ml of streptomycin, and 25 μg/ml of amphotericin B; Gibco), 1% L-glutamine 200 mM, and 10% fetal bovine serum (FBS) or 10% autologous serum (AS) (Gibco). Cultures were maintained at 37°C in a humidified 5% CO2 atmosphere, with medium changes every other day.

After 48 hours, non-adherent cells were discarded, and the adherent layer was washed twice with DMEM-LG. Cells were maintained in culture until 70–80% confluency was reached. Cell harvesting was performed using TrypLE™ Express (Gibco) for 5 min at 37°C. FBS-supplemented culture medium was added to inactivate the enzyme activity. Cells were centrifuged at 500 × g for 10 minutes at 21°C and counted using a Neubauer chamber.

For subsequent passages, cells were plated at the same density (5–6 × 10³ cells/cm²) in T175 or T225 flasks (Passage P1). Next, cells were maintained until they reached 70–80% confluence. After this period, trypsinization was performed, and the cells were plated in three T175 or T225 flasks (Passage P2) and maintained until they reached 70–80% confluence. Each T175 or T225 flask was then trypsinized, and three new flasks were plated (Passage P3). Passage 4 was performed if needed. In the final passage, autologous serum replaced FBS to eliminate residual animal protein from the final product (Fig 1).

The final yield was 10 million (1 x 10^7^) cells per sample/patient, prepared for periurethral injection. Cells were suspended in sterile PBS, with a small aliquot reserved for viability and quality control tests (see protocol below).

Transportation protocols ensured maintenance of sterility and cellular integrity. The final product was sealed in a 20 mL syringe and packaged with recyclable ice and a temperature logger, in compliance with UN3373 regulations. Upon arrival at the UNIFESP-EPM outpatient clinic, cell viability and transport temperature were verified to ensure adherence to specified conditions.

#### Quality control tests for cellular products.

Comprehensive quality and safety assessments of the cellular products intended for clinical use were conducted in accordance with guidelines established by the Foundation for the Accreditation of Cell Therapy (FACT) and the Brazilian National Health Surveillance Agency (ANVISA) [25]. These tests encompassed identity verification, genetic stability analysis, viability assessment, potency evaluation, endotoxin detection, and microbiological screening. Only cellular samples that met all specified quality control criteria were approved and released for clinical application. Samples falling to meet these standards were discarded. These procedures reflect contemporary recommendations for human cell therapy products and are consistent with current regulatory principles for advanced cell-based interventions, although the present study was not designed as a full regulatory development program for an advanced therapy medicinal product.

##### 1- Identity test:

MSCs characterization followed the International Society for Cellular Therapy (ISCT) guidelines [17]. The release criteria for MSC required a specific cell surface expression profile, as follows: positive markers CD105 (endoglin), CD73 and CD90; negative markers CD14, CD34, CD45, CD19 and HLA-DR [17]. The following antibodies were used for immunophenotyping (BD Pharmingen): CD14-APC, CD19-PECy5, CD29-APC, CD31-FITC, CD45-FITC, CD73-PE, CD90-PE, CD105-FITC, CD166-PerCP-Cy5, KDR (CD309)-PE, HLA-DR-PECy5. Cells at passage 3 (P3) were resuspended in a staining solution containing PBS supplemented with 1% FBS and 0.05% sodium azide and incubated for 30 min at room temperature in the dark. After incubation, cells were washed with PBS (Gibco) and centrifuged at 500 × g for 5 minutes (Eppendorf). The cell pellet was resuspended in 200 µL of PBS (Gibco) for analysis.

**Fig 2:**
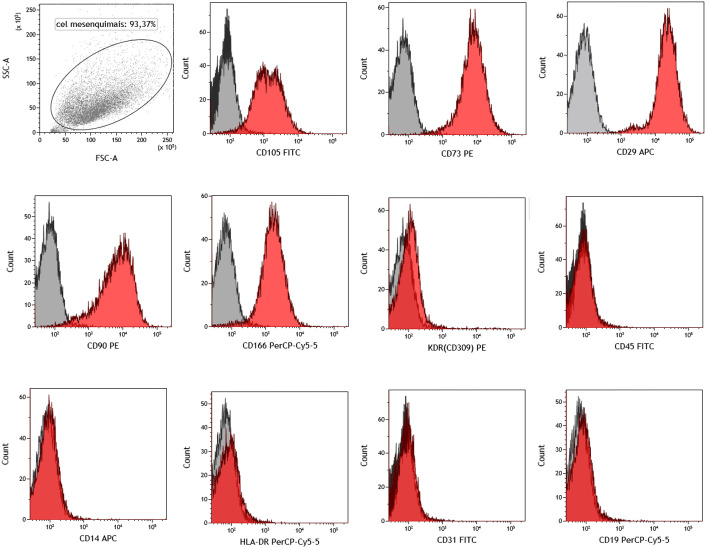
Identity test for characterization of the MSCs. Immunophenotyping by flow-cytometry of MSCs. Similar antibodies profile for the SkM-MSCs and BM-MSCs. The red histograms indicate the percentage of the cell population positive for each antibody, while the grey histograms represent unmarked cells control. The antigenic expression profile confirmed the nature of undifferentiated MSCs. Positive markers: CD29, CD73, CD90, CD105, and CD166. Negative markers: CD14, CD19, CD31, CD45, KDR-CD309, and HLA-DR.

Flow cytometry analysis was performed using a FACS Canto II flow cytometer (BD Biosciences, San Jose, CA, USA). Data acquisition and analysis were conducted using Kaluza software (Beckman-Coulter). A minimum of 10,000 events were acquired for each sample (Fig 2). Cells that did not conform to the established MSC immunophenotypic profile were excluded from further use in the study.

##### 2- Genetic stability:

To ensure genetic integrity, cell samples at the infusion passage were analyzed for clonal abnormalities using karyotyping of 20 metaphases, in accordance with International Standing Committee on Human Cytogenomic Nomenclature (ISCN) 2020 criteria [26]. Clonal abnormalities were defined as: at least three metaphases with the absence of the same chromosome, or two metaphases with the same additional chromosome, or the same structural alteration.

The Standard G-band karyotyping involved culturing MSCs in T-25 flasks until 70–80% confluence. Cell synchronization into the G1 phase was achieved by replacing the culture medium with FBS-free medium for 20 hours, followed by an additional 30 hours of culture in FBS-containing medium for mitotic arrest. Metaphase arrest was induced using KaryoMAX™ Colcemid™ (Gibco) to disrupt spindle formation. Cells were then harvested using TrypLE (Gibco), subjected to hypotonic treatment with 0.075 M KCl for chromosome spreading, and fixed using a 3:1 methanol:acetic acid solution. Fixed cells were dropped onto glass slides, air-dried, and subjected to G-banding using Giemsa staining to generate characteristic chromosome banding patterns. Analysis involved examining 20 metaphases per sample under a light microscope using 100x oil immersion objective lens. Chromosomes were arranged in pairs (22 pairs of autosomes + 1 pair of sex chromosomes) and analyzed for abnormalities.

A karyotype was considered abnormal in two or more metaphases showed clonal chromosomal aberrations (≥10% of metaphases). Non-clonal chromosomal alterations (<10% of metaphases with identical alterations) were assessed individually, with further analysis by Fluorescence In Situ Hybridization (FISH) or repeat karyotyping as needed.

**Fig 3:**
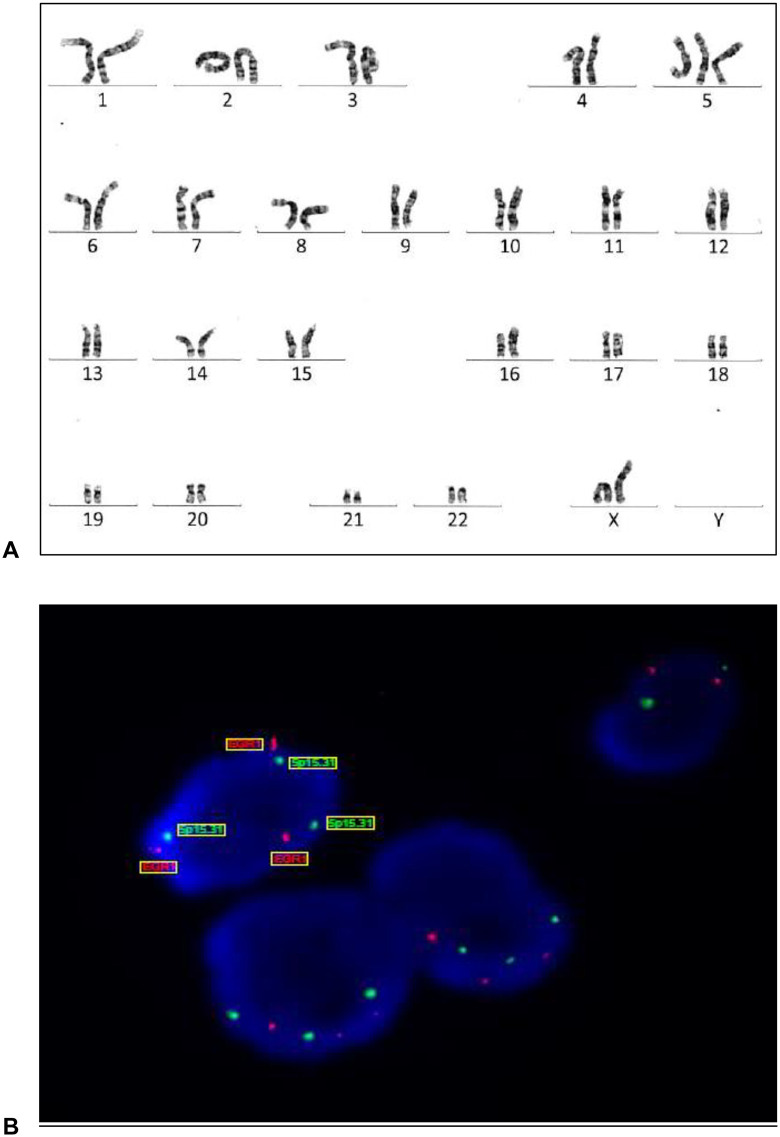
Genetic stability tests. Report of karyotyping confirmed by FISH tests detecting genetic instability in cultured MSCs cells derived from bone-marrow, showing trisomy 5. **(A)** G-banding karyotype analysis: number of metaphases karyotyped and analyzed: 20; result: 47, XX, + 5 [2]/ 46, XX [18]. **(B)** Fluorescence In Situ Hybridization (FISH): Nuclear in situ hybridization using probes D5S630-D5S2064 and BGR1, revealing three copies (x3 – trisomy) of chromosome 5 in 39 out of 100 analyzed nuclei.

FISH was employed as a complementary technique to confirm or further cha24racterize karyotyping findings. This molecular cytogenetic method detects and localizes specific DNA sequences on chromosomes using fluorescently labeled DNA probes. The FISH protocol included cell fixation on slides, DNA denaturation to allow probe binding, application of fluorescence-labeled DNA probes, washing and counterstaining with DAPI, and analysis under a fluorescence microscope to confirm clonal abnormalities identified in karyotyping (Fig 3).

Evidence of genetic instability precludes the clinical use of the cellular product.

##### 3- Viability and cell count:

Immediately prior to infusion, cell viability and total count were evaluated using the Trypan Blue exclusion method. The procedure involved preparing a cell suspension by diluting cells in PBS, then mixing this suspension with 0.4% Trypan Blue dye (Gibco) at a 1:1 ratio. The stained cell suspension was loaded onto a Neubauer chamber and examined under an EVOS microscope (ThermoScientific) to distinguish between viable (unstained, transparent) and non-viable (blue-stained) cells. Both viable and non-viable cells were counted in the four corner squares of the hemocytometer. Cell viability was calculated as the percentage of live cells relative to the total cell count, while the total number of viable cells was calculated accounting for the dilution factor and chamber volume. After the fourth passage (P4), a target yield of 10 million cells was obtained. Stringent quality control measures were applied, with only samples demonstrating viability exceeding 80% being approved for clinical application. This threshold ensures the delivery of a highly viable cell population, potentially optimizing therapeutic efficacy and minimizing the introduction of cellular debris or non-functional cells into the treatment site.

##### 4- Potency test:

**Fig 4:**
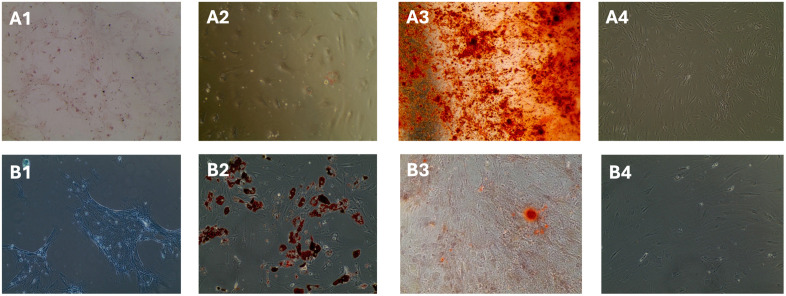
Differentiation potential test for MSCs. Multilineage differentiation of SkM-MSCs (A) and BM-MSCs **(B)**. (A1,B1) Chondrogenic differentiation was demonstrated by the presence of proteoglycans stained with toluidine blue or Safranin **O.** (A2,B2): Adipogenic differentiation was confirmed by the formation of intracytoplasmic lipid droplets stained with Oil Red **O.** (A3,B3): Osteogenic differentiation was evidenced by calcium deposition visualized by Alizarin Red staining. (A4, B4): Negative control showing undifferentiated cells without induction.

MSC cultures at passage 3 (P3) were subjected to multi-lineage differentiation assays, confirming their capacity to differentiate into adipocytes, osteoblasts, and chondrocytes (Fig 4).

**Adipocyte Differentiation:** For adipogenic differentiation, MSCs were seeded in triplicate in 12-well culture plates (Corning) at a density of 4 × 10^4^ cells/well. After 48 hours, the medium was replaced with an adipogenic induction medium (StemPro® Adipogenesis Differentiation Kit, Gibco), with one well serving as a negative control maintained in basal medium with serum and antibiotics. The medium was changed every three days for 21 days. Cells were then fixed in 4% paraformaldehyde, washed with PBS, and stained with 0.3% Oil Red O stain (Fisher Scientific) to visualize intracellular lipid granules.

**Osteocyte Differentiation:** Osteogenic differentiation was initiated by seeding MSCs in triplicate onto 12-well plates at a density of 2 × 10^4^ cells/well. After 48 h, the medium was switched to an osteogenic inducing medium (StemPro® Osteogenesis Differentiation Kit, Gibco), with one well serving as a negative control in regular basal medium with serum and antibiotics. Following 21 days of culture, cells were fixed with 4% paraformaldehyde and stained with 1% Alizarin Red S (Acros Organics) to detect calcium deposits.

**Chondrocyte Differentiation:** MSC at passage 3 (P3) were seeded in triplicate onto 12-well plates to create micromass cultures at a density of 3,75 × 10^6^ cells/mL or, alternatively, for monolayer cultures at a density of 2 × 10^4^ cells/well. Chondrogenic induction was achieved by replacing the medium with StemPro® Condrogenesis Differentiation Kit (Gibco), with one well serving as a negative control in regular basal medium with serum and antibiotics. After 21 days, cells were fixed with 4% paraformaldehyde and stained with Alcian blue or Safranin O (Fisher Scientific) to detect proteoglycan deposits.

All differentiation assays were analyzed using an inverted microscope EVOS M5000 (Invitrogen) to confirm successful lineage-specific differentiation and validate the multi-lineage potential of the MSCs.

##### 5- Endotoxin test:

Endotoxin levels were quantified using the Endosafe nexgen-PTS system (Charles River), a spectrophotometric method employing Limulus amebocyte lysate (LAL) cartridges (Endosafe-PTS). For each assessment, a 25 μL sample was applied to each of the four cartridge wells. Samples exhibiting endotoxin levels below 5.0 EU/mL were suitable for clinical application, adhering to established safety thresholds (S3 File).

##### 6- Microbiological control:

Screening was conducted using the BD Bactec FX system (BD Biosciences), wherein samples from the culture medium were incubated under both aerobic and anaerobic conditions. The system monitored microbial growth over a five-day period through CO₂ fluorescence detection, providing a comprehensive assessment of potential bacterial or fungal contamination. Additionally, mycoplasma testing was performed using real-time RT-PCR with primers specific to *Mycoplasma pneumoniae*, a common contaminant in cell cultures. This molecular approach ensured high sensitivity and specificity in detecting mycoplasma presence. Any cultures showing evidence of microbial contamination, including mycoplasma, were excluded from the study, maintaining the integrity and safety of the cellular products used in clinical applications.

### Interventions

#### Periurethral injection of stem cells.

The SCs therapy was delivered through periurethral injection in only one occasion – single dose. Either cell products containing SkM-MSCs or BM-MSCs was administered in an outpatient setting at UNIFESP-EPM by the researchers (RCS, YM) under strict aseptic conditions. The procedure began with the introduction of 5–10 mL of 2% lidocaine hydrochloride gel into the urethra, followed by catheterization to empty the bladder. The urinary catheter was retained to facilitate urethral palpation and prevent cell extravasation during the injection process. To enhance SCs integration and activation, three initial punctures were made at each planned injection site using a 12x40mm needle, creating localized trauma. Subsequently, the cellular product, consisting of 10 million cells suspended in 10 mL of sterile PBS was strategically distributed as follows: 3 mL paraurethrally on the right side (3 o’clock position), 3 mL on the left (9 o’clock position), and 4 mL in the lower region (6 o’clock position). This precise distribution resulted in localized tissue bulging, corresponding to the injected volume and ensuring optimal placement of the SCs in the target area. The delivery took around 15 minutes to be completed for every patient. This procedure was implemented exclusively within the context of the present pilot clinical study.

### Sample size

The sample size for this pilot study was primarily determined based on the primary outcome of developing protocols for stem cell extraction, culture, and quality control. Drawing from the researchers’ experience, an initial empirical estimate of 10 samples per group was deemed sufficient for these purposes. It is important to note that the sample size was not calculated to analyze the clinical aspects of the therapies, as this was a secondary endpoint.

### Statistical analysis

For statistical analysis of clinical outcomes, variables of interest were presented in tables showing absolute and relative frequency distributions. Intragroup analyses of outcomes were conducted using McNemar’s test for categorical variables and Wilcoxon signed-rank test for numerical variables, and included only the participants which completed the 12-month follow-up. A significance level of 5% (p < 0.05) was established for all statistical tests. Data management involved compiling collected information into a database developed in Excel® for Windows. All statistical analyses were performed using STATA® 11 SE software, ensuring robust reproducible statistical processing of the study data.

In this study, the unit of assignment and the unit of analysis were both the individual participant, as outcomes were analyzed within each intervention group using pre- and post-treatment comparisons. No statistical comparisons were made between the treatment groups; thus, there was no discrepancy between the unit of assignment and the unit of analysis. Intragroup comparisons were conducted to evaluate changes over time within each group.

## Results

**Fig 5:**
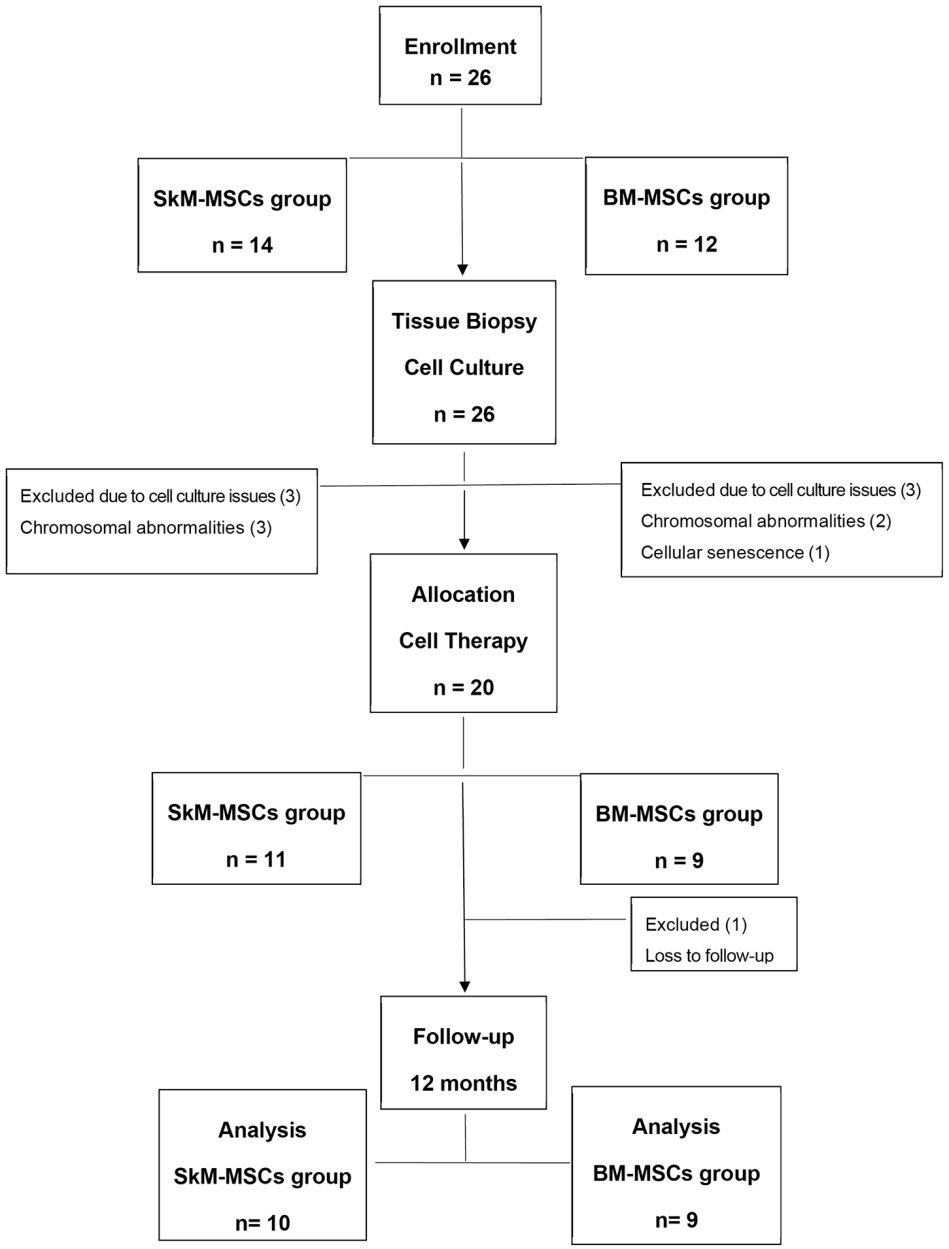
Flow diagram of participants enrollment, allocation, follow-up, and analysis in our pilot study evaluating autologous stem cell therapy for stress urinary incontinence.

This pilot study included twenty-six patients diagnosed with stress urinary incontinence. Of these participants, 14 underwent muscular biopsy for SkM-MSCs group, while 12 underwent bone marrow biopsy for the BM-MSCs group. Ultimately, 11 women received SkM-MSCs therapy, and 9 received BM-MSCs therapy. The 12-month follow-up was completed by 10 participants from the SkM-MSCs group and 9 from the BM-MSCs group (Fig 5). Table 1 presents the demographic and clinical data of the study groups containing only the participants that were treated.

**Table 1:** Clinical, demographic, gynecological, and obstetrical characteristics of study groups.

	SkM-MSCs group (n = 11)	BM-MSCs group (n = 9)
Age (years) – mean ± SD	56.6 ± 10.2	49.0 ± 10.8
BMI (kg/m²) – mean ± SD	33.7 ± 8.5	28.9 ± 6.2
Deliveries – mean ± SD	2.3 ± 1.2	1.8 ± 1.3
Vaginal delivery – mean ± SD	1.5 ± 1.6	1.7 ± 1.2
Forceps delivery – mean ± SD	0.4 ± 0.8	0.4 ± 0.7
Postmenopausal status – n (%)	7 (63.6)	5 (55.6)
Hormone replacement therapy – n (%)	1 (9.1)	0
Sexually active – n (%)	8 (72.7)	7 (77.8)
Smoking – n (%)		
Yes	0 (0)	1 (11.1)
No	10 (90.9)	7 (77.8)
Former	1 (9.1)	1 (11.1)
Mixed urinary incontinence – n (%)	3 (27.3)	5 (55.6)

Continuous variables are described by mean and standard deviation and categorical variables by number and proportion. n: number of participants. SD: standard deviation. Data considering participants who received cellular therapy.

The diagram illustrates the progression of participants from initial screening through allocation into the two treatment arms: skeletal muscle-derived mesenchymal stem cells (SkM-MSCs) and bone marrow-derived mesenchymal stem cells (BM-MSCs). The number of patients who underwent biopsy, received cellular therapy, and completed the 12-month follow-up for each group was detailed, providing a clear visual representation of our study.

### Primary endpoints

Autologous MSC products were successfully obtained for the treatment of women with SUI. Eleven final products containing 1 x 10^7^ SkM-MSCs and 9 final products with an equivalent number of BM-MSCs were successfully manufactured.

Rigorous quality control measures were implemented to ensure the safety and efficacy of the cell products. Among the SkM-MSCs cultures, three out of 14 cell cultures (21.4%) derived from muscular samples were discarded. The observed abnormalities included gain of chromosome X, trisomy 7, and tetraploidy. Similarly, in BM-MSCs, three out of 12 samples (25%) fail to meet the requirements for clinical use. The reasons for exclusion were chromosomal abnormalities (two cases of trisomy 5) and cellular senescence (one culture).

The final autologous MSCs products that passed all control tests (Figs 1-4), were deemed suitable for administration within this pilot clinical study. These products demonstrated genetic stability, absence of chromosomal abnormalities, and appropriate cellular viability and proliferation. All analyzed samples were confirmed to be free of microbial contamination with no evidence of bacterial or fungal growth. Real-time RT-PCR testing for Mycoplasma pneumoniae yielded negative results for all samples. Endotoxin levels in all samples were consistently below 5.0 EU/mL, in full compliance with safety standards for clinical use (S1 Appendix). This comprehensive selection process ensured that only high-quality, genetically stable MSC products were used for therapeutic intervention in the study participants. However, a further limitation of this work is that residual bovine proteins were not quantified by ELISA in the final cell products despite the use of FBS during expansion, representing an important safety and quality consideration for translational application of MSC-based therapies.

### Secondary endpoints

#### Efficacy.

The efficacy of MSC therapy for SUI was evaluated in two groups: SkM-MSCs and BM-MSCs (Table 2). In the SKM-MSCs group, the proportion of participants with a positive cough test for urinary leakage decreased significantly from 100% at a baseline to 40% at 12 months (p = 0.010). The average pad test weight in this group reduced from 8.3 ± 10.3 g to 2.5 ± 3.0 g (p = 0.250), with 40% of patients achieving at least 50% reduction in pad weight after 12 months. Quality of life scores improved from 68.6 ± 14.4 at baseline and 79.9 ± 19.4 at the 12-month follow-up, although this change was not statistically significant (p = 0.546).

**Table 2:** Clinical outcomes of the study groups.

	SkM-MSCs group n = 10	BM-MSCs group n = 9
**Positive cough test**		
Baseline – n/ total n (%)	10/10 (100)	8/9 (88.9)
After 12 months – n/ total n (%)	4/10 (40)	7/9 (77.8)
p value•	0.010*	0.526
**Pad test**		
Baseline – mean ± SD	8.3 ± 10.3	10.4 ± 10.9
After 12 months – mean ± SD	2.5 ± 5.5	2.4 ± 3.0
p value••	0.250	0.120
**I-QoL**		
Baseline – mean ± SD	68.6 ± 14.4	63.1 ± 17.4
After 12 months – mean ± SD	79.9 ± 19.4	66.9 ± 21.4
p value••	0.546	0.955
**≥ 50% weight reduction in pad test after 12 months** – n/ total n (%)	4/10 (40)	5/9 (55.6)
**Urinary Urgency**		
Baseline – n (%)	7/10 (70)	5/9 (55.6)
After 12 months – n/ evaluated n (%)	3/10 (30)	3/9 (33.3)
***Denovo***	0	0
**Urinary Frequency** (≥ 7/ day)		
Baseline – n (%)	6/10 (54.5)	5/9 (55.6)
After 12 months – n/ total n (%)	5/10 (50)	5/9 (55.6)
***Denovo***	3	1
**Urge incontinence**		
Baseline – n (%)	3/10 (30)	5/9 (55.6)
After 12 months – n/ total n (%)	1/10 (10)	3/9 (33.3)
***Denovo***	1	0
**Nocturia** (≥ 2/ night)		
Baseline – n (%)	6/10 (54.5)	3/9 (33.3)
After 12 months – n/ total n (%)	3/10 (30)	0/9 (0)
***Denovo***	1	0
**Adverse effects**		
**Post biopsy – 2-week period** – n/ evaluated n	10/10	9/9
Site pain	6/10	5/9
Site bleeding/ ecchimosys/ haematoma	2/10	2/9
Site infection	0/10	0/9
**Post periurethral injection – 2-week period** – n/ evaluated n	10/10	5/5
Site pain	6/10	0/5
Site bleeding/ ecchimosys/ haematoma	8/10	1/5
Site infection	0/10	0/5
Dysuria	6/10	1/5
Urinary urgency	5/10	1/5
UTI	0/10	0/5
Voiding dysfunction/ obstruction	1/10	0/5

Per protocol analysis: analysis of the participants who completed the 12-month follow-up. n: number of participants. SD: standard deviation. • McNemar’s test; •• Wilcoxon signed-rank test. *P value < 0.05 as significant.

In the BM-MSCs group, the percentage of patients with a positive cough test decreased from 88.9% at baseline to 77.8% after 12 months (p = 0.526). The average pad test weight in this group reduced from 10.4 ± 10.9 g to 2.4 ± 3.0 g in 12 months (p = 0.120), with 55% of patients achieving at least a 50% reduction in pad weight. Quality of life scores in BM-MSCs group showed a modest, non-significant improvement from 63.1 ± 17.4 at baseline to 66.9 ± 21.4 in 12 months (p = 0.955). These results suggest potential efficacy of MSC therapy for SUI, with the SkM-MSCs group demonstrating more pronounced improvements in some outcome measures compared to the BM-MSCs group.

### Adverse effects

The adverse effects following tissue biopsies and periurethral injection therapy are summarized in Table 2. Overall, the incidence of symptoms was low, with reported effects being predominantly mild and transient, resolving spontaneously in the short-term. It is important to note that due to the COVID-19 pandemic, complete data collection for the 3- and 6-month assessments was not feasible, potentially limiting the comprehensive evaluation of short-term adverse effects.

At the 12-month follow-up, no patients reported persistent adverse effects such as local pain, leukorrhea, dysuria, bleeding, difficulty initiating urination or straining to urinate, voiding dysfunction, or de novo lower urinary tract symptoms. Importantly, there was no evidence of tumor formation at the injection site of the injection or in other tissues, addressing a critical safety concern for cell-based therapies. These findings suggest a favorable safety profile for the MSC therapy in the treatment of SUI, with no significant long-term adverse effects observed in this cohort. However, the limitations in data collection due to the pandemic should be considered when interpreting these results, and further long-term follow-up studies may be warranted to confirm the sustained safety of this approach.

### Discontinuation of the study

The study initially included tissue biopsies from 26 participants, which successfully established feasibility protocols for extracting stem cells from adult tissues and preparing cellular products containing muscle- and bone marrow-derived stem cells. Despite demonstrating safety in preclinical assays, the clinical study was discontinued after administering cell therapy to 20 patients due to several factors. Primarily, we were affected by the pandemic period. Furthermore, the modest long-term clinical efficacy outcomes did not meet the anticipated therapeutic potential. Furthermore, the study encountered significant challenges during the optimization of protocols for cell extraction, cultivation, and quality control testing. These challenges let to sample losses and disruptions in cell cultures, resulting in substantial financial burden and budgetary constraints. The labor-intensive and time-consuming nature of these procedures, which was not fully anticipated at the study’s outset, further contributed to the decision to discontinue. These combined factors – limited long-term efficacy, technical challenges, financial constraints, and resource-intensive processes – ultimately led to the premature termination of the study. This experience highlights the complex nature of translating stem cell therapies from bench to bedside and underscores the importance of comprehensive planning and resource allocation in clinical trials involving advanced cellular therapies. These considerations underline that the present protocol should be regarded as an exploratory approach whose main role is to inform future, more definitive and regulation-compliant trials, rather than a regimen ready for routine clinical adoption.

## Discussion

Cell therapy using adult stem cells represents a vital area of Regenerative Medicine. Its potential has been widely demonstrated in advanced research using BM-MSCs in the fields of Cardiology and Rheumatology with studies showing improvement in left ventricular function post-acute myocardial infarction [27,28], and important improvement in pain, quality of life, and cartilage tissue volume in patients with osteoarthritis [29,30]. In addition to the encouraging results, it has proven to be a safe therapy, with only mild side effects and no severe complications reported [27–30].

Our aim was to obtain an autologous multipotent undifferentiated mesenchymal stem-cells derived from skeletal muscle and from bone marrow for use in stress urinary incontinence management in women, and to describe reproducible protocols for cell product preparation that could be compared to others.

Prior to the use of the stem cells therapy in clinical practice, it is crucial to characterize the cells and assess their safety and viability [18,19]. In accordance with Brazilian regulations set forth by ANVISA [25], we conducted rigorous tests including identity verification, karyotype analysis, microbiological and endotoxin assessments conducted under Good Manufacturing Practice guidelines [17,24,25], and viability assays of the stem cells.

By minimizing culture passages, we followed Neri’s recommendations [31] to maintain cellular efficacy and reduce risks associated with genetic instability as excessive passages reduce the replication potential of cells and their multipotent capacity. Binato et al [32] demonstrated that genetic instability may occur after four passages, and that cells have reduced multipotent capacity after P8, concluding that genetic stability is maintained until P4, when the cells reached their best therapeutic potential. Most of our cells were obtained in early passages, which minimized the risk of genetic instability. Nevertheless, we observed chromosomal aberrations in two BM-MSCs and three SkM-MSCs cultures, suggesting that additional factors may be involved in genetic instability. Indeed, the literature has reported abnormal karyotypes in up to 20% of MSCs from bone-marrow in large scale culture [33–35].

The implications of these aberrations in cell therapy are not fully understood. Neri et al [31] stated that karyotype can lead to either malignant transformation or senescence. To our knowledge, tumor formation has never been reported among the numerous clinical trials using MSCs (autologous or allogeneic), making this occurrence highly unlikely. The tendency towards senescence, however, can potentially reduce the effectiveness of cell therapy.

Our group has confirmed the benign behavior of genetically abnormal cells in an animal model [36]. By injecting the trisomy 5 BM-MSCs from our samples into “nu-nu” mice, which lack an effective immune system and are incapable of preventing tumor formation, the altered cells did not cause any harm, and the animals remained healthy for an extended period. In the same experiment, the immunomodulatory capacity of the aberrant cells remained unchanged. Therefore, in principle there would be no greater risk in their use, and they might retain some therapeutic effect. It is important to emphasize that none of the altered samples were injected into our patients, despite their apparent safety.

The method of obtaining and characterizing the cells, along with strict adherence to potency and quality tests, directly influence their regenerative capacity [37]. Unfortunately, precise description of the methodology for obtaining cell products derived from adult tissues have been incompletely reported in most studies of SUI therapy [6–8,10,12] posing an issue for reproducibility and comparison between the studies.

Adult tissue-specific stem cells are rare and generally do not display characteristic morphology or surface markers that would readily distinguish them from mature cells. They cannot, therefore, be readily ‘isolated’ from any given tissue, but a variety of protocols have succeeded in enriching stem/progenitor cells to different degrees of purity [14]. Most investigations in SUI reported have reported the use of so-called muscle-derived cells, muscular progenitor cells or myoblasts [38–40] which are unipotent cell committed to unique muscular lineage, therefore regenerating only the striated musculature [5]. This differs from the effect of our multipotent cells, which, in theory, could regenerate the urethral sphincter, the supporting fascial tissues of the urethra, and the vascularization and innervation [5].

The results of our study, however, were not optimal. Both the response to the cough test and the pad test were modest compared to the existent treatments for SUI, whether using SkM-MSCs or BM-MSCs. Quality of life also showed slight improvement with both therapies after 12 months.

These results agree with the literature, especially regarding the use of SkM-MSCs [6–12]. We highlight the work of Sharifiagdas et al [9,11], who, like our study, demonstrated the use of undifferentiated MSC derived from skeletal muscle injected at the urethral sphincter of women with SUI via endoscopy [11]. Cell identification, characterization by immunophenotyping, karyotype chromosomal analysis, microbiological and endotoxin tests were also performed as recommended. Twenty patients received 50 x 10^6^ cells and were followed for 24 months. The findings resemble ours, with a relevant reduction in the average weight of the 1-hour pad test baseline: between grades 3 (10-20g weight) and 4 (> 20g); 24-months: grade 2 (2-10g); p < 0.0001]. Quality of life showed significant improvement in both the Urinary Incontinence Impact Questionnaire (IIQ-7) and Urogenital Distress Inventory (UDI-6) questionnaires after 12 and 24 months. Nevertheless, there was a tendency for quality of life to worsen over the years, highlighting the limitation of this therapy in achieving complete continence [11].

Despite different methodologies, our results were comparable with other studies that used “muscle-derived cells” which do not necessarily indicates multipotent cells [7,8,40]. Peters et al [8] conducted a dose-response study (1 x 10⁷ to 20 x 10⁷ muscle-derived cells) evaluating a total of 80 women with SUI followed for a year. They reported improvement in the pad test, with at least 50% reduction in pad weight in 20% of women who received the lowest dose, and 64% treated with the highest dose. A negative pad test was observed in 7 out of 22 (32%) women receiving the highest dose. Quality-of-life improvement (around 50% decrease in the mean scores of IIQ-7 and UDI-6 questionnaires) was detected irrespective of the dose.

Regarding adverse effects, our study’s findings are consistent with the literature on SUI [6,8,10,12], which reported mild and transient symptoms post therapy with periurethral injection of myocytes. We found no severe side effects after one year, aligning with reviews affirming the safety of MSC treatments [14–16].

There are no studies using BM-MSCs for SUI for comparison purposes.

Taken together, although safe and encouraging, the efficacy of the cell therapy in SUI management remains modest, which led us to critically analyze different aspects of the cell therapy conducted so far. Moreover, our results should be taken interpreted with caution as this is a pilot study, with a small sample size.

Several factors may hinder the production of cell products and, consequently, cell therapy. The donor’s age is the most important factor to be considered, due to the greater difficulty in obtaining and expanding cells with sufficient quality and quantity for clinical use [18,41,42]. Moreover, systemic conditions such as obesity, rheumatoid arthritis, diabetes and other chronic inflammatory diseases appear to reduce the efficacy of MSCs [18,43]. Considering that the profile of patients with SUI often includes women over 50 and obese patients with concomitant diseases, this may have affected our *in vitro* and clinical results.

To date, there is no consensus on the optimal number of cells or local injections to achieve maximal efficacy in SUI management. It has been shown that a greater number of myoblasts injected in the periurethral region in women correlates with better clinical response [7,8]. Weather our stem cell therapy would have resulted in better outcomes by using more cells remains to be investigated.

The mechanism of action of MSCs may also have influenced our clinical results. Stem cells act through immunomodulation (pro- or anti-inflammatory actions) and trophic activity (activation of cell proliferation, angiogenesis, reduction of apoptosis, reduction of oxidative stress). These mechanisms are mainly triggered by acute changes in the injured tissues such as trauma, ischemia or inflammation, which respond by releasing chemokines and cytokines that activate the stem cells [42]. The structures into which cells are injected in SUI cases are long-term scarred and quiescent and therefore do not present the ideal conditions for MSC activation and fixation. Furthermore, the minimal trauma caused by the injection needle does not seem to be sufficient to trigger cellular action.

Neri et al [31] list the factors for obtaining the best possible results in cell therapy: culture conditions marked by lower number of passages, the method of cells application, and donor conditions. Following the first factor cited, we use cells with the lowest possible number of passages. Regarding local cell injections, we envision possibilities for future research in SUI: a greater number of cells offered by multiple injections; the addition of growth factors to our cell products, which likely enhance the therapeutical effects; a more aggressive method of offering cells to the urethral sphincter to trigger the mechanism of action of the MSCs such as the use of scaffolds seeded with MSCs under the mid-urethra. Moreover, allogeneic cells from healthy donors can be considered for cases involving older women and/or women with other chronic diseases. With this, we disclose and share the challenges in cell therapy reported by Choi et al [44] in a recent review.

In summary, this pilot clinical study demonstrates that autologous SkM‑MSCs and BM‑MSCs can be manufactured under rigorous quality control and delivered periurethrally to women with SUI with an acceptable short‑term safety profile, but with modest and variable clinical benefit. The detailed manufacturing and testing procedures may serve as a structured starting point for future research; however, they should not be interpreted as a definitive clinical protocol for routine SUI management. Further, we agree with other authors that larger and randomized trials, fully aligned with current regulatory frameworks for human cell therapy, are required before MSC‑based approaches can be considered for broader clinical implementation in this indication [18,44,45].

## Conclusions

The development of an autologous cellular products composed of undifferentiated SkM-MSCs and BM-MSCs, properly validated through quality control tests in accordance with national and international standards, is viable. However, the process is highly demanding, time-consuming, and financially burdensome. We confirmed the feasibility and safety of periurethral injection of autologous SkM-MSCs and BM-MSCs for the treatment of SUI. Consistent with other cell therapy investigations, this pilot study demonstrated that the cell products and the methodology used resulted in modest effects in the benefiting women with SUI, as assessed by both objective and subjective measurements.

Our findings showed the potential of stem cell therapy in SUI management while also underscoring the challenges involved in translating this approach into clinical practice. The rigorous methodology employed in cell preparation and characterization provides a foundation for future studies, emphasizing the importance of standardized protocols in the field. Despite the modest clinical outcomes, the short-term safety profile and the insights gained from this study contribute valuable knowledge to the ongoing development of cell-based therapies for SUI.

Future research should focus on optimizing cell delivery methods, exploring the use of growth factors, biomaterials, scaffolds seeded with MSCs, and investigating the potential of allogeneic stem cells from healthy donors. Additionally, further studies are needed to elucidate the mechanisms of action of MSCs in the context of SUI and to determine the optimal cell dosage and injection protocols. As we continue to refine these approaches, cell therapy may yet emerge as a promising treatment option for women with SUI.

## Supporting information

S1 File Standard Operation Protocol – Processing and Expansion of Skeletal Muscle-Derived Mesenchymal Stromal Cells. (PDF)

S2 File Standard Operation Protocol – Processing and Expansion of Bone Marrow-Derived Mesenchymal Stromal Cells. (PDF)

S3 File Endotoxin assay – parameters used. (PDF)

S1 Appendix Supporting Information Files. (ZIP)
