# Supplementary material for: Stem cell therapy for female stress urinary incontinence: Results, limitations and lessons learned from a pilot clinical study
Source: PLoS One. 2026 Feb 27;21(2):e0342452. doi: 10.1371/journal.pone.0342452 (PMC12948050; doi:10.1371/journal.pone.0342452)
Supplement: S1 File — (PDF) [file pone.0342452.s001.pdf]

SOP Title: Isolation, Expansion, and Transport of Skeletal Muscle-Derived Mesenchymal Stem/Stromal Cells (SkM-MSCs) from Quadriceps Biopsies

1. PURPOSE

This SOP describes the standardized procedure for collection of skeletal muscle biopsies, isolation of muscle-derived mesenchymal stem/stromal cells (SkM-MSCs), their in vitro expansion under Good Manufacturing Practice (GMP) conditions, and preparation/transport for clinical application. This method ensures reproducibility, cell viability, and compliance with safety and sterility requirements.

2. SCOPE

Applicable to all clinical and laboratory personnel performing SkM-MSC extraction, culture, and handling at GMP-certified facilities, specifically from quadriceps femoris muscle (vastus lateralis) samples.

3. RESPONSIBILITIES

- **Medical team:** Perform the biopsy under aseptic conditions, ensure proper sample collection and packing.

- **Cell processing staff:** Receive, process, culture, expand, and prepare final product under GMP/ISO 5 conditions.

- **Quality control team**: Monitor temperature logs, sterility, viability, and product release criteria.

4. SAFETY PRECAUTIONS

- Follow biosafety level 2 (BSL-2) procedures.

- Wear sterile gown, gloves, mask, hair cover, and goggles.

- Work inside a Class II biological safety cabinet (ISO 5 environment) for all aseptic manipulations.

- Dispose of all biological waste according to institutional and governmental regulations.

5. MATERIALS AND REAGENTS

Biopsy Collection:

- 2% lidocaine hydrochloride + norepinephrine hemitartrate (local anesthetic)

- 3-0 polyglactin (Vicryl) suture and 4-0 or 2-0 nylon sutures

- Sterile surgical instruments

- Phosphate-buffered saline (PBS) 1X, pH 7.4 (Gibco)
- Antibiotic–antimycotic solution (10,000 U/mL penicillin, 10 mg/mL streptomycin, 25 µg/mL amphotericin B) (Gibco)
- Gentamicin solution (10 mg/mL) (Gibco)
- Sterile collection tubes

Cell Isolation and Culture:

- Class II laminar flow hood
- Collagenase type (0.075% w/v) (Sigma) prepared in PBS with Ca^{2+} and Mg^{2+} (Sigma)
- DMEM/F12 medium (Gibco)
- Fetal bovine serum (FBS), USDA-tested, 15% (HyClone)
- Non-essential amino acids (NEAA) (Gibco)
- TrypLE Express (Gibco)
- 50 mL conical tubes (Sarstedt)
- Petri dishes
- T25, T75, T225 culture flasks (Corning)
- Neubauer counting chamber
- Parafilm
- CO_2 incubator (37°C, 5% CO_2, humidified) (Thermo Fisher)

Transport of Final Product:

- Sterile Luer-lock syringes (20 mL, BD)
- Sterile Luer caps (BD)
- Primary sealed sterile container + secondary sealed plastic bag (ziplock)
- UN3373-certified outer transport box
- Recyclable ice packs (Gelox)
- Temperature logger (Kooltrak)

6. PROCEDURE

6.1 Skeletal Muscle Biopsy Collection

1. Perform biopsy in an outpatient setting under aseptic conditions at authorized clinical facility.

2. Identify the biopsy site at the vastus lateralis region of the quadriceps femoris.

3. Disinfect skin; drape with sterile fields.

4. Infiltrate skin and deeper tissues with 5–10 mL of 2% lidocaine hydrochloride (+ norepinephrine hemitartrate) as local anesthetic.

5. Perform a ~3–4 cm skin incision to access subcutaneous tissue.

6. Dissect and retract subcutaneous tissue and fat to expose fascia lata.

7. Incise fascia lata and identify underlying muscle.

8. Excise a muscle fragment of ~1.0 × 1.0 cm.

9. Immediately transfer the fragment to a sterile 50 mL tube containing:

- PBS 1X pH 7.4
- 2% antibiotic–antimycotic (penicillin/streptomycin/amphotericin B)
- 0.05% gentamicin

10. Close fascia with 3-0 polyglactin sutures, skin with nylon sutures (simple interrupted), apply a compressive dressing.

11. Schedule suture removal at postoperative day 15.

6.2 Transport of Biopsy to GMP Facility

1. Place the primary container in a UN3373-compliant insulated box with recyclable ice, maintaining 4–24°C.

2. Include a temperature logger (Kooltrak) inside the package.

3. Ensure delivery to GMP-certified cell processing facility within 24 h of collection.

6.3 Muscle-Derived MSC Isolation

1. Inside a Class II laminar flow hood, decontaminate external vial surface with 70% ethanol.

2. Using sterile forceps, transfer the muscle fragment to a sterile Petri dish or new tube.

3. Record sample weight and volume using a precision balance.

4. Wash the tissue twice with sterile PBS 1X containing:

- 0.05% gentamicin
- 1% antibiotic–antimycotic solution

5. Mince tissue into small (~1 mm^3) fragments using a sterile disposable scalpel.

6. Transfer minced tissue to a 50 mL tube containing 0.075% collagenase solution in PBS (with Ca^{2+} and Mg^{2+}).

7. Seal tube with Parafilm and incubate at 37°C in a water bath for 30 min.

8. Agitate every 10 min using vortex mixing.

9. After digestion, add PBS to stop enzymatic reaction.

10. Remove undigested fragments with sterile forceps.

11. Centrifuge at 280 × g for 5 min at room temperature.

12. Discard supernatant; resuspend pellet in 1 mL complete culture medium (DMEM/F12 + 15% FBS + 1% antibiotic–antimycotic + 1% NEAA).

13. Count viable cells using a Neubauer chamber.

6.4 Primary Culture (Passage 0 – P0)

1. Plate cells in a T25 culture flask (or 6-well plate for initial seeding). Consider cell density at $5x10^3$ cells/cm^2.

2. Incubate at 37°C, 5% CO_2, humidified atmosphere.

3. Change medium every 2–3 days.

4. When 70–80% confluent, detach cells with TrypLE Express:

   - Wash with PBS
   - Add enough TrypLE to cover surface
   - Incubate 5–8 min at 37°C until cells detach
   - Neutralize with medium containing FBS

5. Collect cells with sterile Pasteur pipette and transfer to larger flasks.

6.5 Cell Expansion

1. After primary culture (Passage 0 – P0) reaches 70–80% confluence, detach cells using the standardized TrypLE Express procedure (see Section 6.4).

2. Resuspend cells in complete culture medium and reseed into larger flasks at a 1:3 split ratio, calculated according to the total available surface growth area. This ensures optimal cell density for continued proliferation without inducing early senescence or differentiation.

3. **Expansion workflow:**

   - **P1:** Transfer from T25 to T75 flasks (1:3 passaging ratio by growth area).
   - **P2:** Transfer from T75 to T225 flasks (1:3 ratio).
   - **P3–P5:** Continue expansion in T225 flasks, maintaining the 1:3 split ratio at each passage, until the target cell yield is reached.

4. At each passage:

- Incubate cultures at 37 °C in a 5% CO_2 humidified environment.

- Replace culture medium every 2–3 days (or as needed to maintain pH and nutrient levels).

- Perform visual inspection to monitor morphology, confluence, and absence of microbial contamination.

5. Continue expansion until approximately 10 × 10^6 viable cells are obtained (typically at P5–P6), meeting the **Critical Quality Parameters** outlined in Section 7 prior to product release.

6.6 Preparation of Final Product for Transport

1. At final passage (P5–P6), resuspend cells in sterile PBS at desired concentration in a 20 mL Luer-lock syringe sealed with a sterile Luer cap.

2. Place syringe into sealed primary sterile bag, then into a sealed secondary bag (ziplock).

3. Place inside an insulated container with recyclable ice packs and a temperature logger.

4. Package in UN3373-compliant transport box labeled with all required regulatory information.

5. Ship using specialized biomedical transport.

7. CRITICAL QUALITY PARAMETERS

Critical quality parameters must be rigorously evaluated before the release of the final advanced therapy medicinal product (ATMP) comprising muscle-derived mesenchymal stem/stromal cells (MSCs). These parameters ensure cell safety, identity, potency, and sterility, and comply with regulatory standards established by regulatory agencies.

The critical quality attributes to be analyzed include:

- **Cell quantity:** The total number of viable cells must meet the predefined minimum threshold for therapeutic efficacy, commonly expressed as millions of cells per dose.

- **Cell viability**: Viability should be assessed using validated methods (e.g., Trypan Blue exclusion or equivalent) and must exceed 80% at the point of release to guarantee sufficient live cell content for clinical use.

- **Microbiological testing:** The product must undergo sterility testing according to pharmacopeial guidelines to exclude contamination by bacteria and fungi, ensuring a sterile final product.

- **Endotoxin testing:** Quantification of bacterial endotoxins (e.g., Limulus Amebocyte Lysate assay) is mandatory to confirm levels below established safety limits, preventing pyrogenic reactions upon administration.

- **Mycoplasma testing:** Sensitive molecular or culture-based assays are required to detect and exclude mycoplasma contamination, a common cell culture contaminant with potential patient risks.

- **Immunophenotyping:** Flow cytometry analysis must demonstrate the expected MSC surface marker profile, typically positive expression of CD73, CD90, CD105, and negative for hematopoietic and endothelial markers (e.g., CD34, CD45, CD14, CD19, HLA-DR), confirming cell identity.

- **Karyotype analysis:** Cytogenetic evaluation should confirm genomic stability and absence of chromosomal abnormalities that could imply malignant transformation or genetic instability during cell expansion.

These quality control tests must be performed under validated conditions using qualified equipment and trained personnel. Release criteria should be clearly defined and aligned with regulatory guidance to assure that only safe, efficacious, and high-quality MSC products proceed to clinical application. Documentation of all testing results and conformity assessments is essential for traceability and regulatory compliance.

8. CHANGE HISTORY

SOP no.	Effective Date	Significant Changes	Previous SOP no.
**B.153 V.0**	**5/7/18**	**Initial version**	**NA**
