# Supplementary material for: Stem cell therapy for female stress urinary incontinence: Results, limitations and lessons learned from a pilot clinical study
Source: PLoS One. 2026 Feb 27;21(2):e0342452. doi: 10.1371/journal.pone.0342452 (PMC12948050; doi:10.1371/journal.pone.0342452)
Supplement: S2 File — (PDF) [file pone.0342452.s002.pdf]

Standard Operation Protocol

Processing and Expansion of Bone Marrow-Derived Mesenchymal Stromal Cells

1. Purpose and Scope

This SOP describes the Good Manufacturing Practice (GMP)-compliant procedure for the processing, culture, expansion, and maintenance of bone marrow-derived mesenchymal stromal cells (BM-MSCs) intended for clinical applications.

2. Responsibilities

It is the responsibility of the trained and authorized cell processing personnel to strictly follow this SOP, ensure proper documentation, and maintain aseptic conditions throughout the process.

3. Materials and Equipment

- Equipment

- Class A2 Biological Safety Cabinet (BSC)
- CO_2 incubator
- Centrifuge with temperature control
- Neubauer counting chamber

- Materials

PRODUCT	MANUFACTURER	CATALOG NUMBER
antibody monoclonal CD14 - APC Mouse Anti-Human CD14<br>Clone M5E2	BD Pharmingen™	555399
antibody monoclonal CD19 - PerCP-Cy™5.5 Mouse Anti-Human CD19<br>Clone HIB19	BD Pharmingen™	561295
antibody monoclonal CD31 - FITC Mouse Anti-Human CD31<br>Clone WM59	BD Pharmingen™	555445
antibody monoclonal CD34 (4H11), PE, eBioscience™	Invitrogen	12034942
antibody monoclonal CD44 - CD44 Monoclonal Antibody (MEM-85), FITC	Invitrogen	MHCD4401

antibody monoclonal CD45 - FITC Mouse Anti-Human CD45<br>Clone HI30	BD Pharmingen™	560976
antibody monoclonal CD73 - PE Mouse Anti-Human CD73<br>Clone AD2 (RUO)	BD Pharmingen™	550257
antibody monoclonal CD90 - PE Mouse Anti-Human CD90<br>Clone 5E10	BD Pharmingen™	555596
antibody monoclonal CD105 - FITC Mouse anti-Human CD105<br>Clone 266	BD Pharmingen™	561443
antibody monoclonal CD309 - PE Mouse Anti-Human CD309 (VEGFR-2)<br>Clone 89106	BD Pharmingen™	560494
antibody monoclonal HLA-ABC - HLA-ABC Monoclonal Antibody (W6/32), APC	Invitrogen	17-9983-42
antibody monoclonal HLA-DR (LN3), PerCP-Cyanine5.5, eBioscience™	Invitrogen	45995642
Needle 1,20x25	BD	305243
Antibiotic-Antimycotic (100X)	Gibco	15240062
BD BACTEC™ Plus Aerobic medium	BD Bactec	442023
BD BACTEC™ Plus Anaerobic medium	BD Bactec	442022
BD MYCO/F LYTIC	BD Bactec	442206
Cell Strainer - Falcon® 100 µm Yellow, Sterile, Individually Packaged	Corning	352360
Corning® 500 mL Vacuum Filter/Storage Bottle System, 0.22 µm Pore	Corning	430769
Criovial - Corning® Internal Thread Cryogenic Vials 1.2 mL	Corning	430487
CryoPur™ DMSO Solutions	Origen	CP-50
DMEM, low glucose, pyruvate	Gibco	11885084
Endosafe Cartridge	Charles River	PTS2005F
Flask T25 - Corning® 25cm² Rectangular Canted Neck Cell Culture Flask with Vent Cap	Corning	430639
Flask T75 - Corning® 75cm² U-Shaped Canted Neck Cell Culture Flask with Vent Cap	Corning	430641U
Flask T225 - Corning® 225 cm² Angled Neck Cell Culture Flask with Vent Cap	Corning	431082
Bottle 250 mL (sterile square media bottle, PETG)	Life Solutions	2019-0250
Ficoll Paque Plus	GE Healthcare	17144002
Bottle Filter 500 mL	Millipore	S2GPT05RE
LAL REAGENT WATER (30ML)	Charles River	W130
L-Glutamina	Life Solutions	25030081
Oil Red O	Sigma Aldrich	O0625
PBS (phosphate buffered saline), pH 7.4	Gibco	10010023

Serological pipette 5 ml	Sarstedt	861.253.001
Serological pipette 10 ml	Sarstedt	861.254.001
Serological pipette 25 ml	Sarstedt	861.685.001
Culture plate 12 wells	Sarstedt	833.921
Biosphere® Fil. Tip 1000 (ponteira com filtro)	Sarstedt	70.762.211
Syringe 3 mL	BD	990174
Syringe 60ml BD LUER LOCK sem agulha	BD	302827
Physiological Solutiona 0,9% 250 mL	Baxter	AZB1322
Fetal Bovine Serum, qualified, Brazil (500 mL)	Gibco	12657029
StemPro™ Osteogenesis Differentiation Kit	Gibco	A1007201
StemPro™ Chondrogenesis Differentiation Kit	Gibco	A1007101
StemPro™ Adipogenesis Differentiation Kit	Gibco	A1007001
TrypLE™ Express Enzyme (1X), no phenol red (100 mL)	Gibco	12604013
Conical tube 15 ml	Sarstedt	62.554.205
Conical tiube 50 ml	Sarstedt	62.547.254

4. Procedure

4.1 Starting Material Processing

The starting material is transported into the Clean Room, and a sample is taken for microbiological testing. The tests performed are:

- Aerobic microorganisms
- Anaerobic microorganisms and fungi
- Special microorganisms
- PCR for mycoplasma

The starting material is filtered using a cell strainer to remove any potential clots that may have formed. The volume of the starting material is recorded before and after filtration. The filtered bone marrow is diluted in PBS (phosphate-buffered saline) at a 1:1 ratio.

The diluted sample is transferred into a 50 mL conical tube pre-filled with 15 mL of Ficoll-Paque Plus (GE Healthcare). This tube is then centrifuged at 300g for 30 minutes at 21°C, allowing the separation of the mononuclear fraction from the bone marrow (Figure 1).

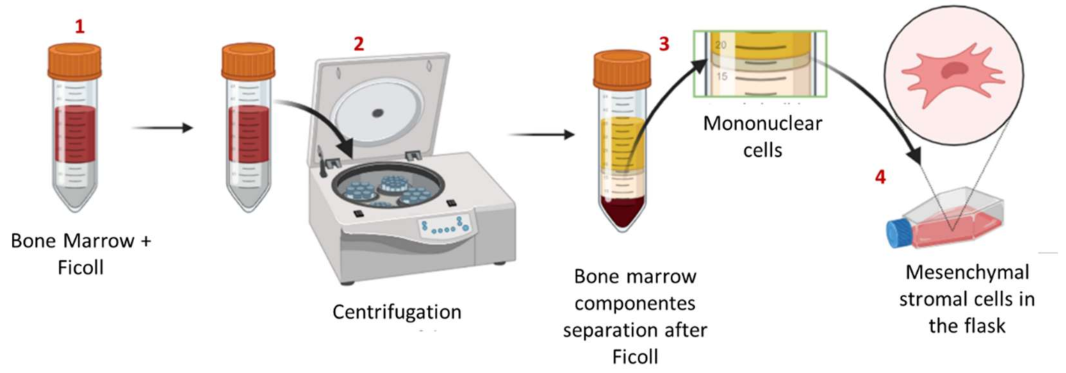

Figure 1. Schematic representation of density gradient centrifugation. (1) A volume of 30 mL of bone marrow is diluted in medium or PBS at a 1:1 ratio and added to a 50 mL conical tube previously filled with 15 mL of Ficoll. (2) This tube is centrifuged for 30 minutes at 300g. (3) The mononuclear cell layer is then collected, washed in PBS, and (4) seeded into a culture flask.

The mononuclear cells are washed and centrifuged again at 300g for 10 minutes at 21°C. Cell counting is performed using a Neubauer chamber with Trypan Blue according to the formula below:

Calculation:

___________ /_____ ___________ X ___________ X ____________ X ________ = _____ X 10^4 / 100 = _____ X 10^6

(Counted cells) (Numer of Squares Counted) (Dilution Factor) (Trypan Dilution Factor) (Initial Volume)

All processing is conducted under a Class A2 biological safety cabinet, where a settling plate is placed inside the airflow and later sent to an external company for microbiological analysis. In addition to the settling plate, the operator performs monthly fingertip and palm microbiological testing.

4.2 Initial Cell Seeding (Passage P0)

Cells should be seeded at a density of 5–6 × 10^3 cells/cm² into two T175 culture flasks. The culture medium used must be DMEM low glucose supplemented with 10% fetal

bovine serum (FBS), previously sterilized through a 0.22 µm PES membrane filter to ensure the removal of potential contaminants. Prior to use, all flasks must be clearly labeled with the donor's initials, passage number, bag number, flask number, product designation, processing date, and the name of the responsible operator, ensuring full traceability throughout the process.

4.3 Cell Culture Maintenance

Cultures must be maintained in a CO_2 incubator set at 37°C with 5% CO_2 until reaching 70–80% confluence. The culture medium should be replaced every two days under a biological safety cabinet (BSC) to ensure aseptic handling. During the first medium change, a sample of the culture must be collected and sent for microbiological testing, including assays for aerobic and anaerobic microorganisms, fungi, special microorganisms, and mycoplasma detection.

4.4 Trypsinization and Expansion

To passage the culture, first remove the medium and wash the cells with PBS to eliminate residual serum proteins. Add TrypLE Express to the flask and incubate for 5–8 minutes at 37°C, 5% CO_2, until the cells detach from the surface. Neutralize the enzymatic activity by adding culture medium containing FBS, then transfer the cell suspension to a 50 mL conical tube and centrifuge at 500g for 10 minutes at 21°C. Following centrifugation, count the viable cells and reseed at a density of 5–6 × 10^3 cells/cm^2. Continue the expansion process through successive passages up to Passage P5, with the goal of obtaining approximately 9–10 × 10^6 cells per final serynge.

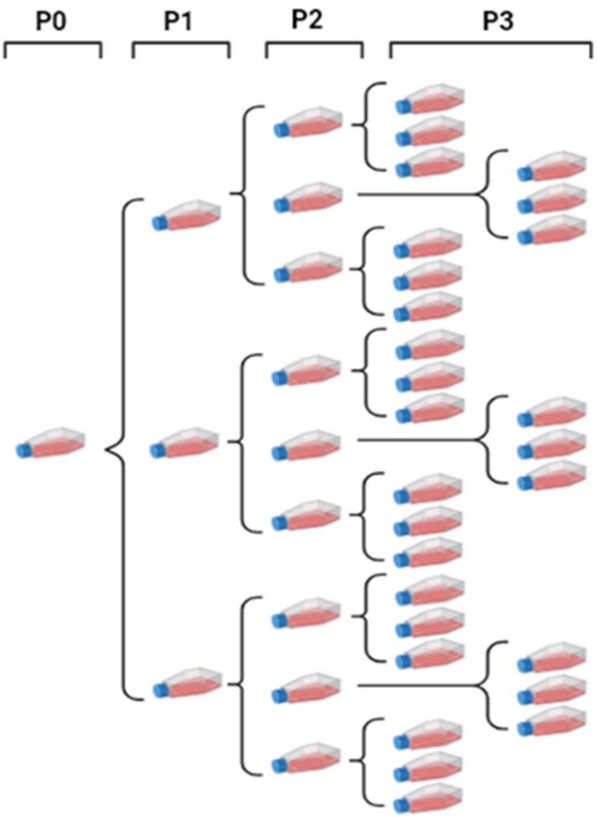

Figure 2. Schematic of mesenchymal stromal cell (MSC) expansion from bone marrow. MSCs are typically isolated by their ability to adhere to plastic surfaces, allowing their expansion by increasing the number of plastic vessels (thus increasing the surface area for adherence). Culture flasks are easy to handle and allow for gas exchange through a filter-cap in the CO_2 incubator.

5. Quality Control

- **Microbiological testing** at two critical points in the beginning of the process: upon receipt of the starting material and after the first medium change. Testing must include:
  - Aerobic microorganisms
  - Anaerobic microorganisms and fungi
  - Special microorganisms
  - Mycoplasma detection by validated PCR assay
- **Viral safety testing** including screening for human viral markers for donor eligibility.

- **In-process control documentation**, including:
    - Culture conditions (medium composition, supplements, CO_2 and temperature settings)
    - Passage numbers and seeding densities
    - Dates of all manipulations
    - Operator identification for each step
    - Yields at each passage and total final cell count
- **Final product testing** before release for clinical use, including:
    - Identity (immunophenotyping by flow cytometry)
    - Purity (percentage of target cell population, absence of undesired cells)
    - Viability (trypan blue exclusion or flow cytometry viability dyes)
    - Potency assay relevant to the product's mechanism of action (MSC differentiation into adipocytes, chondrocytes and osteocytes)
    - Mycoplasma (PCR)
    - Endotoxins (LAL assay)
    - Aerobic microorganisms
    - Anaerobic microorganisms and fungi
    - Special microorganisms
