# Supplementary material for: Stem cell therapy for female stress urinary incontinence: Results, limitations and lessons learned from a pilot clinical study
Source: PLoS One. 2026 Feb 27;21(2):e0342452. doi: 10.1371/journal.pone.0342452 (PMC12948050; doi:10.1371/journal.pone.0342452)
Supplement: S3 File — (PDF) [file pone.0342452.s003.pdf]

charles river
accelerating drug development. exactly.

Endotoxin

ASSAY DATA			
Assay Date/Time:	26-Feb-20 9:43:46 AM	File Name:	ng16320019202002260943346.pltx
Lab Name:	HOSPITAL ALBERT EINSTEIN-RADIOFA	Operator:	Marcela A
Serial Number:	PTS16320019	Temperature (°C):	37.0:37.0

SAMPLE DATA			
Product Name:	5168	Product Lot Number:	5168
		Conc./Dil.:	1:100 mL/mL

CURVE DATA			
Cartridge Lot Number:	8596127	Calibration Code:	513242756911
Standard Curve (EU/mL)	5 - 0.05	Seconds:	132 - 827
Y-Intercept:	+2.39	Slope:	-0.398

RESULTS							
Sample				Spike			
Reaction Time(Sec):		>827	>827	Reaction Time(Sec):		272	286
CV%:	0.0	Limit:	25	CV%:	3.5	Limit:	25
Sample Value (EU/mL):		<5.00		Spike Value (EU/mL):		0.714 EU/mL	
Endotoxin Limit (EU/mL):		N/A		Recov. %:	80	Range %:	50-200

CONCLUSION				
SAMPLE CV	Endotoxin (EU/mL)	Endotoxin Limit	Recovery	SPIKE CV
VALID	<5.00	N/A	VALID	VALID

Comments:

Analyst:	Reviewer:
Signature: ______________	Signature: ______________
Date: ______________	Date: ______________
