# Supplementary material for: Stem cell therapy for female stress urinary incontinence: Results, limitations and lessons learned from a pilot clinical study
Source: PLoS One. 2026 Feb 27;21(2):e0342452. doi: 10.1371/journal.pone.0342452 (PMC12948050; doi:10.1371/journal.pone.0342452)
Supplement: S1 Appendix — (ZIP) [file pone.0342452.s004.zip › Supporting Information Files/Addendum_Project_Final_R1_English.docx]

**Addendum: Methodology**

**Title:** Use of Human Stem Cells in the Treatment of Women with Stress Urinary Incontinence

**Outcomes and Measurements**

**Primary Outcome**

To develop optimized autologous cellular products composed of undifferentiated SkM-MSCs and BM-MSCs, duly validated through quality control tests in accordance with national and international standards, to be safely used as cell therapy in women with SUI.
The quality control tests will include: identity, genetic stability, viability and cell counting, potency, endotoxin detection, and microbiological testing.

**Secondary Outcomes**

To evaluate the feasibility of periurethral injection of autologous SkM-MSCs and BM-MSCs for the treatment of both groups of women with SUI.

To determine the efficacy and safety of cell therapy over a 12-month period.
Clinical findings will be assessed through a cough test, pad test, and quality-of-life questionnaire.

**Methodology**

**Study type, population, and sample**

Prospective, randomized clinical trial involving 45 patients with SUI who will receive periurethral injection of 5 mL of a solution containing 100 million autologous stem cells (derived from muscle, bone marrow, or adipose tissue).

The total number of patients for this pilot clinical study was estimated for convenience.
The sample size for evaluating the primary endpoint of developing protocols for stem cell extraction, culture, and quality control was estimated based on the researchers’ experience. Accordingly, 10 samples per study group were planned for this purpose.

**Quality Control Tests for Cellular Products**

Comprehensive quality and safety assessments of the cellular products intended for clinical use will be carried out. These tests will include identity verification, genetic stability analysis, viability assessment, potency evaluation, endotoxin detection, and microbiological screening. Only cellular samples that meet all specified quality control criteria will be approved and released for clinical application. Samples failing to meet these standards will be discarded.

**Identity Test**

MSCs will be characterized according to the guidelines of the International Society for Cellular Therapy (ISCT) (1). The release criteria for MSCs will require a specific cell surface expression profile: positive markers CD105, CD73, and CD90; negative markers CD14, CD34, CD45, CD19, and HLA-DR.

The following antibodies will be used for immunophenotyping (BD Pharmingen): CD14-APC, CD19-PECy5, CD29-APC, CD31-FITC, CD45-FITC, CD73-PE, CD90-PE, CD105-FITC, CD166-PerCP-Cy5, KDR (CD309)-PE, HLA-DR-PECy5. Cells at passage 3 (P3) will be resuspended in a staining solution containing PBS supplemented with 1% FBS and 0.05% sodium azide, and incubated for 30 min at room temperature in the dark. After incubation, the cells will be washed with PBS (Gibco) and centrifuged at 500×g for 5 minutes (Eppendorf). The cell pellet will be resuspended in 200 µL of PBS (Gibco) for analysis.
Flow cytometry will be performed using a FACS Canto II cytometer (BD Biosciences, San Jose, CA, USA). Data acquisition and analysis will be conducted using Kaluza software (Beckman-Coulter). A minimum of 10,000 events will be acquired for each sample. Cells that do not meet the established MSC immunophenotypic profile will be excluded from the study.

**Genetic Stability**

To ensure genetic integrity, cell samples at the infusion passage will be analyzed for clonal abnormalities using karyotyping of 20 metaphases, in accordance with the International Standing Committee on Human Cytogenomic Nomenclature (ISCN) 2020 criteria (2).
Clonal abnormalities will be defined as: at least three metaphases lacking the same chromosome, or two metaphases with the same additional chromosome, or the same structural alteration.

Standard G-band karyotyping will involve culturing MSCs in T-25 flasks until 70–80% confluence. Cell synchronization into the G1 phase will be achieved by replacing the culture medium with FBS-free medium for 20 hours, followed by an additional 30 hours of culture in FBS-containing medium for mitotic arrest. Metaphase arrest will be induced using KaryoMAX™ Colcemid™ (Gibco). The cells will then be harvested using TrypLE (Gibco), subjected to hypotonic treatment with 0.075 M KCl, and fixed with a 3:1 methanol:acetic acid solution. Fixed cells will be dropped onto glass slides, air-dried, and stained with Giemsa to generate the characteristic chromosome banding patterns.

Twenty metaphases per sample will be analyzed under a light microscope with a 100× oil immersion objective. Chromosomes will be arranged in pairs (22 pairs of autosomes + 1 pair of sex chromosomes) and analyzed for abnormalities.

A karyotype will be considered abnormal when two or more metaphases present clonal chromosomal aberrations (≥10% of metaphases). Non-clonal alterations (<10%) will be assessed individually, and complementary analysis by Fluorescence In Situ Hybridization (FISH) may be performed as needed.

FISH will be used as a complementary technique to confirm or further characterize karyotyping findings, employing fluorescently labeled DNA probes for specific chromosomal sequences. Evidence of genetic instability will preclude the clinical use of the cellular product.

**Viability and Cell Count**

Immediately before infusion, cell viability and total count will be assessed using the Trypan Blue exclusion method. A cell suspension will be mixed with 0.4% Trypan Blue dye at a 1:1 ratio and loaded into a Neubauer chamber for counting under an EVOS microscope (ThermoScientific). Viable and non-viable cells will be counted in the four corner squares. Viability will be calculated as the percentage of live cells relative to the total. Only samples with viability greater than 80% will be approved for clinical application.

**Potency Test**

During culture, MSC cultures will be subjected to multilineage differentiation assays for adipocytes, osteoblasts, and chondrocytes.

- **Adipogenesis:** MSCs will be seeded in 12-well plates with adipogenic induction medium (StemPro®); the negative control will be maintained in basal medium. After 21 days, fixation and Oil Red O staining for lipids will be performed.
- **Osteogenesis:** MSCs will be seeded and induced with osteogenic medium (StemPro®). After 21 days, they will be stained with Alizarin Red S for calcium deposits.
- **Chondrogenesis:** MSCs will be cultured as micromass or monolayer, induced with chondrogenic medium (StemPro®). After 21 days, they will be stained with Alcian Blue or Safranin O for proteoglycans.

All assays will be analyzed using an EVOS M5000 inverted microscope (Invitrogen).

**Endotoxin Test**

Endotoxin levels will be quantified using the Endosafe nexgen-PTS system (Charles River) with LAL cartridges. Samples <5.0 EU/mL will be considered suitable for clinical application.

**Microbiological Control**

Screening will be performed using the BD Bactec FX system, incubating samples from the culture medium under aerobic and anaerobic conditions for 5 days, with microbial growth monitored by CO₂ fluorescence detection. Mycoplasma testing will be performed by real-time RT-PCR with primers specific for *Mycoplasma pneumoniae*. Contaminated cultures will be excluded from the study.

**References**

1. Dominici M, Le Blanc K, Mueller I, Slaper-Cortenbach I, Marini F, Krause D, et al. Minimal criteria for defining multipotent mesenchymal stromal cells. The International Society for Cellular Therapy position statement. Cytotherapy. 2006;8(4):315-7.
2. International Standing Committee on Human Cytogenomic Nomenclature. ISCN 2020: An International System for Human Cytogenomic Nomenclature. Karger, 2020. DOI: 10.1159/isbn.978-3-318-06867-2
