# Supplementary material for: Stem cell therapy for female stress urinary incontinence: Results, limitations and lessons learned from a pilot clinical study
Source: PLoS One. 2026 Feb 27;21(2):e0342452. doi: 10.1371/journal.pone.0342452 (PMC12948050; doi:10.1371/journal.pone.0342452)
Supplement: S1 Appendix — (ZIP) [file pone.0342452.s004.zip › Supporting Information Files/Addendum_Projeto_Final_R1.docx]

**Adendo: Metodologia**

**Título: Uso de células-tronco humanas no tratamento de mulheres com incontinência urinária de esforço.**

**Desfechos e Medidas**

**Desfecho Primário**

Desenvolver produtos celulares autólogos otimizados, compostos por SkM-MSCs e BM-MSCs indiferenciadas, devidamente validados por meio de testes de controle de qualidade, em conformidade com padrões nacionais e internacionais, para serem utilizados com segurança como terapia celular em mulheres com IUE. Os testes de controle de qualidade incluem: identidade, estabilidade genética, viabilidade e contagem celular, potência, detecção de endotoxinas e testes microbiológicos.

**Desfechos Secundários**

Avaliar a viabilidade da injeção periuretral de SkM-MSCs e BM-MSCs autólogas para o tratamento de ambos os grupos de mulheres com IUE. Determinar a eficácia e a segurança da terapia celular ao longo de um período de 12 meses. Os achados clínicos foram avaliados por meio de teste de tosse, teste do absorvente e questionário de qualidade de vida.

**Metodologia**

**Tipo de estudo, população e amostra**

Estudo clínico, randomizado prospectivo envolvendo 45 pacientes com IUE que receberão injeção peri-uretral de 5ml de solução contendo 100 milhões de CTA autólogas (derivadas de músculo, medula óssea ou tecido adiposo). O número total de pacientes para o estudo clínico piloto fora estimado por conveniência.

The sample size for evaluating the primary endpoint of developing protocols for stem cell extraction, culture, and quality control was estimated based on the researchers’ experience. Accordingly, 10 samples per study group were planned for this purpose.

**Testes de Controle de Qualidade para Produtos Celulares**

Serão realizadas avaliações abrangentes de qualidade e segurança dos produtos celulares destinados ao uso clínico. Esses testes incluirão verificação de identidade, análise de estabilidade genética, avaliação de viabilidade, teste de potência, detecção de endotoxinas e triagem microbiológica. Somente amostras celulares que atenderem a todos os critérios especificados de controle de qualidade serão aprovadas e liberadas para aplicação clínica. Amostras que não atenderem a esses padrões serão descartadas.

**Teste de Identidade**

A caracterização das MSCs seguirá as diretrizes da *International Society for Cellular Therapy* (ISCT) (1). Os critérios de liberação para MSC exigirão um perfil específico de expressão de marcadores de superfície celular: marcadores positivos CD105, CD73 e CD90; marcadores negativos CD14, CD34, CD45, CD19 e HLA-DR. Serão utilizados os seguintes anticorpos para imunofenotipagem (BD Pharmingen): CD14-APC, CD19-PECy5, CD29-APC, CD31-FITC, CD45-FITC, CD73-PE, CD90-PE, CD105-FITC, CD166-PerCP-Cy5, KDR (CD309)-PE, HLA-DR-PECy5. Células na passagem 3 (P3) serão ressuspensas em solução de coloração contendo PBS suplementado com 1% FBS e 0,05% de azida sódica, e incubadas por 30 min à temperatura ambiente no escuro. Após a incubação, as células serão lavadas com PBS (Gibco) e centrifugadas a 500×g por 5 minutos (Eppendorf). O pellet celular será ressuspenso em 200 µL de PBS (Gibco) para análise. A citometria de fluxo será realizada utilizando um citômetro FACS Canto II (BD Biosciences, San Jose, CA, EUA). A aquisição e análise dos dados serão feitas com o software Kaluza (Beckman-Coulter). Um mínimo de 10.000 eventos será adquirido para cada amostra. Células que não apresentarem o perfil imunofenotípico estabelecido para MSC serão excluídas do estudo.

**Estabilidade Genética**

Para garantir a integridade genética, amostras de células na passagem de infusão serão analisadas quanto a anormalidades clonais por cariotipagem de 20 metáfases, de acordo com os critérios do *International Standing Committee on Human Cytogenomic Nomenclature* (ISCN) 2020 (2). As anormalidades clonais serão definidas como: pelo menos três metáfases com ausência do mesmo cromossomo, ou duas metáfases com o mesmo cromossomo adicional, ou a mesma alteração estrutural.

O cariótipo padrão com bandeamento G envolverá o cultivo das MSCs em frascos T-25 até 70–80% de confluência. A sincronização celular na fase G1 será obtida substituindo o meio de cultura por meio sem FBS por 20 horas, seguida de mais 30 horas de cultivo em meio com FBS para bloqueio mitótico. O bloqueio em metáfase será induzido usando KaryoMAX™ Colcemid™ (Gibco). As células serão então coletadas com TrypLE (Gibco), submetidas a tratamento hipotônico com 0,075 M KCl e fixadas com solução metanol:ácido acético (3:1). As células fixadas serão gotejadas em lâminas, secas ao ar e coradas com Giemsa para geração dos padrões de bandeamento. Serão analisadas 20 metáfases por amostra sob microscópio óptico com objetiva de imersão em óleo 100x. Os cromossomos serão organizados em pares (22 pares de autossomos + 1 par sexual) e analisados quanto a anomalias. O cariótipo será considerado anormal quando duas ou mais metáfases apresentarem alterações cromossômicas clonais (≥10% das metáfases). Alterações não clonais (<10%) serão avaliadas individualmente, podendo ser realizadas análises complementares por *Fluorescence In Situ Hybridization* (FISH).

O FISH será utilizado como técnica complementar para confirmar ou caracterizar achados da cariotipagem, usando sondas de DNA fluorescentes específicas para sequências cromossômicas. Evidência de instabilidade genética excluirá o uso clínico do produto celular.

**Viabilidade e Contagem Celular**

Imediatamente antes da infusão, a viabilidade e a contagem total serão avaliadas pelo método de exclusão com Azul de Tripano. Uma suspensão celular será misturada ao corante a 0,4% na proporção 1:1 e carregada em câmara de Neubauer para contagem no microscópio EVOS (ThermoScientific). Células viáveis e não viáveis serão contadas nos quatro quadrantes. A viabilidade será calculada como a porcentagem de células vivas em relação ao total. Apenas amostras com viabilidade >80% serão aprovadas para aplicação clínica.

**Teste de Potência**

Durante o cultivo, as culturas de MSC serão submetidas a ensaios de diferenciação multilinhagem para adipócitos, osteoblastos e condrócitos.

- Adipogênese: MSCs serão semeadas em placas de 12 poços com meio de indução adipogênica (StemPro®), controle negativo mantido em meio basal. Após 21 dias, ocorrerá fixação e coloração com Oil Red O para lipídios.
- Osteogênese: MSCs serão semeadas e induzidas com meio osteogênico (StemPro®). Após 21 dias, serão coradas com Alizarin Red S para depósitos de cálcio.
- Condrogênese: MSCs serão cultivadas em micromassa ou monolayer, induzidas com meio condrogênico (StemPro®). Após 21 dias, serão coradas com Azul de Alcian ou Safranina O para proteoglicanos.

Todos os ensaios serão analisados no microscópio invertido EVOS M5000 (Invitrogen).

**Teste de Endotoxina**

Os níveis serão quantificados com o sistema Endosafe nexgen-PTS (Charles River) usando cartuchos LAL. Amostras <5,0 EU/mL serão consideradas adequadas para aplicação clínica.

**Controle Microbiológico**

A triagem será realizada no sistema BD Bactec FX, incubando amostras do meio de cultura em condições aeróbias e anaeróbias por 5 dias, monitorando crescimento microbiano via detecção de CO₂ por fluorescência. O teste de micoplasma será feito por RT-PCR em tempo real com *primers* específicos para *Mycoplasma pneumoniae*. Culturas com contaminação serão excluídas do estudo.

**Referências Bibliográficas**

1. Dominici M, Le Blanc K, Mueller I, Slaper-Cortenbach I, Marini F, Krause D, et al. Minimal criteria for defining multipotent mesenchymal stromal cells. The International Society for Cellular Therapy position statement. Cytotherapy. 2006;8(4):315-7.
2. International Standing Committee on Human Cytogenomic Nomenclature. ISCN 2020: An International System for Human Cytogenomic Nomenclature. Karger, 2020. DOI: 10.1159/isbn.978-3-318-06867-2
