# Supplementary material for: Stem cell therapy for female stress urinary incontinence: Results, limitations and lessons learned from a pilot clinical study
Source: PLoS One. 2026 Feb 27;21(2):e0342452. doi: 10.1371/journal.pone.0342452 (PMC12948050; doi:10.1371/journal.pone.0342452)
Supplement: S1 Appendix — (ZIP) [file pone.0342452.s004.zip › Supporting Information Files/Emenda1_PB_PARECER_CONSUBSTANCIADO_CEP_2579812_E1_Ocultado.pdf]

PARECER CONSUBSTANCIADO DO CEP

DADOS DA EMENDA

Título da Pesquisa: Uso de Células-Tronco Adultas no tratamento de mulheres com incontinência urinária de esforço.

Pesquisador: RODRIGO CERQUEIRA DE SOUZA

Área Temática:

Versão: 5

CAAE: 18150613.7.1001.5505

Instituição Proponente: Universidade Federal de São Paulo

Patrocinador Principal: FUNDACAO DE AMPARO A PESQUISA DO ESTADO DE SAO PAULO

DADOS DO PARECER

Número do Parecer: 2.579.812

Apresentação do Projeto:

Trata-e de emenda ao protocolo

A incontinência urinária de esforço (IUE) é a perda de urina involuntária decorrente de algum esforço físico como pular, correr e tossir. IUE afeta 1535% das mulheres, interferindo na sua vida social, psicológica e sexual. O parto vaginal e o envelhecimento tecidual são os principais fatores de risco para o desenvolvimento da IUE por afetar nervos, músculos, vasos e o tecido conectivo do assoalho pélvico, estruturas responsáveis pela manutenção da continência. Há evidências de que os danos principalmente nos músculos estriado e liso da uretra são componentes-chave na patogênese da IUE. Neste cenário, a terapia celular tem sido considerada como uma alternativa para o tratamento da IUE com base na capacidade de restaurar o esfíncter uretral lesionado.

Objetivo da Pesquisa:

Objetivo Primário: Melhora da qualidade de vida de mulheres com incontinência urinária de esforço através de terapia com células-tronco adultas, avaliada com questionário específico validado em língua portuguesa (IQol).

Avaliação dos Riscos e Benefícios:

Riscos: No local de retirada das amostras teciduais e sangue periférico, pode haver dor local de

leve a moderada, e mais raramente pode haver equimoses ou hematomas, que tendem a desaparecer com o tempo. Mais raramente pode ocorrer infecção e inflamação secundária ao procedimento.Esperase que no dia da injeção periuretral o local tenha um pouco de dor, ardência ou desconforto, mas bastante leves. Eventualmente pode haver sangramento na urina, mas também muito leve, e de duração curta. Com o tempo, não deve haver maiores problemas. Não é esperada a formação de tumores, por uso exclusivo de células-tronco adultas. As pacientes receberão anestésicos locais ou sedação na ocasião da realização das biópsias.

Benefícios: Melhora da qualidade de vida de pacientes com IUE. Procedimento minimamente invasivo, utilizando material biológico autólogo, evitando-se os riscos inerentes ao uso de material sintético padrão para correção da IUE. Baixo custo.

Comentários e Considerações sobre a Pesquisa:

Ensaio clínico randomizado, realizado na Escola Paulista de Medicina - Universidade Federal de São Paulo. Durante a avaliação das candidatas ao estudo, serão realizados testes diagnósticos que confirmarão a elegibilidade ao estudo (exame físico, estudo urodinâmico e teste do absorvente) no ambulatório de Uroginecologia, EPM-UNIFESP. Após a entrevista e exame clínico, e a assinatura do termo de consentimento informado, as participantes serão alocadas em um dos 3 grupos do estudo: participantes que receberão células-tronco derivadas de músculo, tecido adiposo ou medula óssea. A randomização será feita por meio de envelopes opacos lacrados numerados de 1 a 45, e para cada paciente será sorteado um envelope no momento da inclusão no estudo. A paciente será encaminhada para realizar o procedimento de coleta do material biológico (biópsias e coleta de sangue periférico), e a seguir o mesmo será enviado ao laboratório. O procedimento de biópsia muscular, ou a biópsia de tecido adiposo que ocorrerão no centro cirúrgico do Hospital São Paulo, ou de biópsia de medula óssea a ser realizada no Setor de Hematologia e Hemoterapia do Hospital Israelita Albert Einstein, São Paulo. A medula óssea será coletada das cristas ilíacas do paciente sob analgesia e anestesia local com a utilização de agulha de biópsia 35G e seringas de 10mL, de acordo com o procedimento padrão utilizado pelos médicos hematologistas. Devem ser realizadas tantas punções quanto forem necessárias até perfazer o volume de 100 mL. O volume obtido por punção não deve ser maior do que 5 mL a fim de evitar a hemodiluição da medula óssea coletada. Os procedimentos de coleta de medula, isolamento e cultivos celulares serão realizados no laboratório de terapia celular do Setor de Hematologia e Hemoterapia do Hospital Israelita Albert Einstein, São Paulo.Amostra de lipoaspirado será coletada após assepsia e antissepsia seguida da infiltração de 50 cc de solução de lidocaína 0,25% com adrenalina 1:

500.000 na região de parede abdominal. O material será aspirado por meio de seringas com cânulas de 3 mm de diâmetro. Os procedimentos de isolamento e cultivos celulares a partir de adipócitos serão realizados no laboratório de terapia celular da Stemcorp, São Paulo.As amostras de músculo: sob efeito de anestesia local, será coletada pequena biópsia muscular (aproximadamente 0,3 cm3) do bíceps do membro superior. Os procedimentos de isolamento e cultivos celulares a partir de adipócitos serão realizados no laboratório de terapia celular da Stemcorp, São Paulo.Após o cultivo das células-tronco, o laboratório enviará ao pesquisador a preparação que conterá 10ml de solução amarelada translúcida.Injeção peri-uretral das diferentes preparações. As preparações dos diferentes grupos de estudo virão em seringas transparentes contendo 5 ml de solução que será injetada na região da uretra média, altura do esfíncter uretral. Sob visão uretroscópica, uma agulha é introduzida pelo aparelho até o local apropriado, e introduzida aproximadamente 3 mm na mucosa. Serão injetados 5 ml na posição 3h e 5ml em 9h, sem obliteração da uretra. O procedimento é ambulatorial, será realizado no Setor de Uroginecologia da UNIFESP - EPM, e não haverá necessidade de anestesia ou analgesia para o procedimento. Sequência: obtenção TCLE, testes pré-aplicação, amostras de tecido, obtenção do soro humano, injeção periuretral, avaliações após 7, 30, 60, 180 dias. Avaliação dos desfechos após 1 ano.

Considerações sobre os Termos de apresentação obrigatória:

Justificativa da Emenda:

Inclusão de centro coparticipante Hospital Santa Marcelina, São Paulo.

Conclusões ou Pendências e Lista de Inadequações:

Trata-se de emenda para inclusão de centro co -participante.

Considerações Finais a critério do CEP:

emenda aprovada

O presente projeto, seguiu nesta data para análise da CONEP e só tem o seu início autorizado após a aprovação pela mesma.

Este parecer foi elaborado baseado nos documentos abaixo relacionados:

Tipo Documento	Arquivo	Postagem	Autor	Situação
Informações Básicas do Projeto	PB_INFORMAÇÕES_BÁSICAS_1054932_E1.pdf	10/03/2018 12:42:39		Aceito
Declaração de	Emenda1_CT.doc	10/03/2018	Maria Augusta	Aceito

Pesquisadores	Emenda1_CT.doc	12:41:32	Tezelli Bortolini	Aceito
Declaração de Pesquisadores	Emenda1_CT.pdf	10/03/2018 12:41:08	Maria Augusta Tezelli Bortolini	Aceito
TCLE / Termos de Assentimento / Justificativa de Ausência	TCLE_SM_CTfinal.docx	10/03/2018 12:14:39	Maria Augusta Tezelli Bortolini	Aceito
Outros	Autorizacao_Armazenamento_MaterialBiologico_HSM.docx	10/03/2018 12:13:37	Maria Augusta Tezelli Bortolini	Aceito
Outros	Declaracao_recrutamento_TCLE.docx	10/03/2018 12:07:43	Maria Augusta Tezelli Bortolini	Aceito
Outros	Coep_CT.pdf	19/01/2017 13:16:54	Maria Augusta Tezelli Bortolini	Aceito
Declaração de Instituição e Infraestrutura	termo_infraestrutura_HIAE.pdf	13/01/2017 17:46:05	Maria Augusta Tezelli Bortolini	Aceito
Declaração de Instituição e Infraestrutura	Declaracao_InfraEstrutura_StemCorp.jpeg	06/12/2016 20:57:54	Maria Augusta Tezelli Bortolini	Aceito
Declaração de Instituição e Infraestrutura	infraestrutura_UNIFESP.jpg	15/11/2016 12:46:23	Maria Augusta Tezelli Bortolini	Aceito
Projeto Detalhado / Brochura Investigador	351lula_tronco_humanos_Castro_v4.pdf	13/10/2016 10:35:55	Maria Augusta Tezelli Bortolini	Aceito
Declaração do Patrocinador	comprovante_Fapesp.pdf	11/10/2016 15:59:45	Maria Augusta Tezelli Bortolini	Aceito
TCLE / Termos de Assentimento / Justificativa de Ausência	TCLE_PF_CT_v4.pdf	11/10/2016 15:56:25	Maria Augusta Tezelli Bortolini	Aceito
Folha de Rosto	FOLHA_ROSTO_CTA_V4.pdf	11/10/2016 15:55:35	Maria Augusta Tezelli Bortolini	Aceito
Outros	Documento_RodrigoCastro2.jpg	24/05/2016 15:07:45	RODRIGO CERQUEIRA DE SOUZA	Aceito
Outros	Documento_RodrigoCastro1.jpg	24/05/2016 15:07:13	RODRIGO CERQUEIRA DE SOUZA	Aceito

Situação do Parecer:
Aprovado

Necessita Apreciação da CONEP:
Sim

SAO PAULO, 04 de Abril de 2018

Assinado por:
Miguel Roberto Jorge
(Coordenador)

Endereço: Rua Francisco de Castro, 55
Bairro: VILA CLEMENTINO **CEP:** 04.020-050
UF: SP **Município:** SAO PAULO
Telefone: (11)5571-1062 **Fax:** (11)5539-7162 **E-mail:** cep@unifesp.edu.br
