# Supplementary material for: Stem cell therapy for female stress urinary incontinence: Results, limitations and lessons learned from a pilot clinical study
Source: PLoS One. 2026 Feb 27;21(2):e0342452. doi: 10.1371/journal.pone.0342452 (PMC12948050; doi:10.1371/journal.pone.0342452)
Supplement: S1 Appendix — (ZIP) [file pone.0342452.s004.zip › Supporting Information Files/Emenda2_PB_PARECER_CONSUBSTANCIADO_CEP_3114724_E2_Ocultado.pdf]

[redacted]

UNIFESP - HOSPITAL SÃO PAULO - HOSPITAL UNIVERSITÁRIO DA

[redacted]

PARECER CONSUBSTANCIADO DO CEP

DADOS DA EMENDA

Título da Pesquisa: Uso de Células-Tronco Adultas no tratamento de mulheres com incontinência urinária de esforço.

Pesquisador: RODRIGO CERQUEIRA DE SOUZA

Área Temática:

Versão: 8

CAAE: 18150613.7.1001.5505

Instituição Proponente: Universidade Federal de São Paulo

Patrocinador Principal: FUNDACAO DE AMPARO A PESQUISA DO ESTADO DE SAO PAULO

DADOS DO PARECER

Número do Parecer: 3.114.724

Apresentação do Projeto:

Trata-de de EMENDA E2 ao protocolo

Breve introdução do estudo, de acordo com o arquivo PB_INFORMAÇÕES_BÁSICAS_1272519_E2.pdf Versão do Projeto: 8, Data de Submissão do Projeto: 06/12/2018

A incontinência urinária de esforço (IUE) é a perda de urina involuntária decorrente de algum esforço físico como pular, correr e tossir. IUE afeta 15- 35% das mulheres, interferindo na sua vida social, psicológica e sexual. O parto vaginal e o envelhecimento tecidual são os principais fatores de risco para o desenvolvimento da IUE por afetar nervos, músculos, vasos e o tecido conectivo do assoalho pélvico, estruturas responsáveis pela manutenção da continência. Há evidências de que os danos principalmente nos músculos estriado e liso da uretra são componentes-chave na patogênese da IUE. Neste cenário, a terapia celular tem sido considerada como uma alternativa para o tratamento da IUE com base na capacidade de restaurar o esfíncter uretral lesionado.

Objetivo da Pesquisa:

Objetivo Primário:

Melhora da qualidade de vida de mulheres com incontinência urinária de esforço através de terapia

com células-tronco adultas, avaliada com questionário específico validado em língua portuguesa (IQol).

Objetivo Secundário:
Melhora da incontinência urinária de esforço medida por testes objetivos (exame físico, teste do absorvente e estudo urodinâmico).

Avaliação dos Riscos e Benefícios:
descritos no parecer de aprovação

Comentários e Considerações sobre a Pesquisa:
Trata-de de EMENDA E2 ao protocolo
Justificativa da Emenda:
Esta emenda visa acrescendar documentos referentes à inclusão da casa de Saude Santa Marcelina de SP como centro coparticipante deste projeto de pesquisa. Complemento da emenda 1.

A PESQUISA SERÁ FINANCIADA PARCIALMENTE PELA FUNDAÇÃO DE AMPARO À PESQUISA DO ESTADO DE SÃO PAULO - FAPESP.
ESTA EMENDA VISA A INCLUSÃO DA CASA DE SAÚDE SANTA MARCELINA COMO CO-PARTICIPANTE DESTA PESQUISA, SENDO O DR. RODRIGO CERQUEIRA DE SOUZA O PESQUISADOR LOCAL. ADICIONAMOS OS DOCUMENTOS SOLICITADOS PELO HOSPITAL SANTA MARCELINA. NA CASA DE SAÚDE SANTA MARCELINA OCORRERÁ AS FASES DE RECRUTAMENTO, SELEÇÃO, BIÓPSIAS, TRATAMENTO E SEGUIMENTO DAS PARTICIPANTES DO ESTUDO.

Considerações sobre os Termos de apresentação obrigatória:
documentos obrigatórios apresentados adequadamente para a respectiva emenda 2

Recomendações:
Nada consta

Conclusões ou Pendências e Lista de Inadequações:
Emenda 2 aprovada

Considerações Finais a critério do CEP:
Parecer acatado pelo colegiado.

O presente projeto, seguiu nesta data para análise da CONEP e só tem o seu início autorizado após a aprovação pela mesma.

Este parecer foi elaborado baseado nos documentos abaixo relacionados:

Tipo Documento	Arquivo	Postagem	Autor	Situação
Informações Básicas do Projeto	PB_INFORMAÇÕES_BÁSICAS_1272519_E2.pdf	06/12/2018 18:41:17		Aceito
Declaração de Pesquisadores	RecrutamentoTCLE.pdf	06/12/2018 18:39:25	Maria Augusta Tezelli Bortolini	Aceito
TCLE / Termos de Assentimento / Justificativa de Ausência	TCLE.pdf	06/12/2018 18:38:35	Maria Augusta Tezelli Bortolini	Aceito
Declaração de Instituição e Infraestrutura	termoHSM.pdf	06/12/2018 18:02:30	Maria Augusta Tezelli Bortolini	Aceito
Declaração de Pesquisadores	Emenda1Adendo.pdf	06/12/2018 17:53:36	Maria Augusta Tezelli Bortolini	Aceito
Declaração de Instituição e Infraestrutura	FolhaRostoSM.pdf	06/12/2018 17:51:35	Maria Augusta Tezelli Bortolini	Aceito
Declaração de Instituição e Infraestrutura	TermoResponsabilidadeSM.pdf	06/12/2018 17:50:52	Maria Augusta Tezelli Bortolini	Aceito
Declaração de Pesquisadores	Pendencia_Emenda1_CEPUNIFESP_CT.doc	19/06/2018 12:26:44	Maria Augusta Tezelli Bortolini	Aceito
Declaração de Pesquisadores	Pendencia_Emenda1_CEPUNIFESP_CT.pdf	19/06/2018 12:25:55	Maria Augusta Tezelli Bortolini	Aceito
Declaração de Pesquisadores	Pendencia_Emenda1_CT.doc	22/05/2018 15:18:50	Maria Augusta Tezelli Bortolini	Aceito
Declaração de Pesquisadores	Pendencia_Emenda1_CT.pdf	22/05/2018 15:18:12	Maria Augusta Tezelli Bortolini	Aceito
Declaração de Pesquisadores	Emenda1_CT.doc	10/03/2018 12:41:32	Maria Augusta Tezelli Bortolini	Aceito
Declaração de Pesquisadores	Emenda1_CT.pdf	10/03/2018 12:41:08	Maria Augusta Tezelli Bortolini	Aceito
Outros	Coep_CT.pdf	19/01/2017 13:16:54	Maria Augusta Tezelli Bortolini	Aceito
Declaração de Instituição e Infraestrutura	termo_infraestrutura_HIAE.pdf	13/01/2017 17:46:05	Maria Augusta Tezelli Bortolini	Aceito
Declaração de Instituição e Infraestrutura	Declaracao_InfraEstrutura_StemCorp.jpeg	06/12/2016 20:57:54	Maria Augusta Tezelli Bortolini	Aceito
Declaração de Instituição e Infraestrutura	infraestrutura_UNIFESP.jpg	15/11/2016 12:46:23	Maria Augusta Tezelli Bortolini	Aceito
Projeto Detalhado / Brochura Investigador	351lula_tronco_humanos_Castro_v4.pdf	13/10/2016 10:35:55	Maria Augusta Tezelli Bortolini	Aceito
Declaração do	comprovante_Fapesp.pdf	11/10/2016	Maria Augusta	Aceito

Patrocinador	comprovante_Fapesp.pdf	15:59:45	Tezelli Bortolini	Aceito
TCLE / Termos de Assentimento / Justificativa de Ausência	TCLE_PF_CT_v4.pdf	11/10/2016 15:56:25	Maria Augusta Tezelli Bortolini	Aceito
Folha de Rosto	FOLHA_ROSTO_CTA_V4.pdf	11/10/2016 15:55:35	Maria Augusta Tezelli Bortolini	Aceito
Outros	Documento_RodrigoCastro2.jpg	24/05/2016 15:07:45	RODRIGO CERQUEIRA DE SOUZA	Aceito
Outros	Documento_RodrigoCastro1.jpg	24/05/2016 15:07:13	RODRIGO CERQUEIRA DE SOUZA	Aceito

Situação do Parecer:
Aprovado

Necessita Apreciação da CONEP:
Sim

SAO PAULO, 17 de Janeiro de 2019

Assinado por:
Miguel Roberto Jorge
(Coordenador(a))

Endereço: Rua Francisco de Castro, 55
Bairro: VILA CLEMENTINO **CEP:** 04.020-050
UF: SP **Município:** SAO PAULO
Telefone: (11)5571-1062 **Fax:** (11)5539-7162 **E-mail:** cep@unifesp.edu.br
