# Supplementary material for: Stem cell therapy for female stress urinary incontinence: Results, limitations and lessons learned from a pilot clinical study
Source: PLoS One. 2026 Feb 27;21(2):e0342452. doi: 10.1371/journal.pone.0342452 (PMC12948050; doi:10.1371/journal.pone.0342452)
Supplement: S1 Appendix — (ZIP) [file pone.0342452.s004.zip › Supporting Information Files/Emenda3_PB_PARECER_CONSUBSTANCIADO_CEP_3817706_E3_Ocultado.pdf]

PARECER CONSUBSTANCIADO DO CEP

DADOS DA EMENDA

Título da Pesquisa: Uso de Células-Tronco Adultas no tratamento de mulheres com incontinência urinária de esforço.

Pesquisador: RODRIGO CERQUEIRA DE SOUZA

Área Temática:

Versão: 10

CAAE: 18150613.7.1001.5505

Instituição Proponente: Universidade Federal de São Paulo

Patrocinador Principal: FUNDACAO DE AMPARO A PESQUISA DO ESTADO DE SAO PAULO

DADOS DO PARECER

Número do Parecer: 3.817.706

Apresentação do Projeto:

Projeto CEP/UNIFESP

Trata-se de emenda (E3) ao projeto:

As informações elencadas nos campos “Apresentação do Projeto”, “Objetivo da Pesquisa” e “Avaliação dos Riscos e Benefícios” foram retiradas do arquivo Informações Básicas da Pesquisa (PB_INFORMAÇÕES_BÁSICAS_1309795_E3.pdf, de 06/03/2019).

BREVE APRESENTAÇÃO DO PROJETO:

A incontinência urinária de esforço (IUE) é a perda de urina involuntária decorrente de algum esforço físico como pular, correr e tossir. IUE afeta 15- 35% das mulheres, interferindo na sua vida social, psicológica e sexual. O parto vaginal e o envelhecimento tecidual são os principais fatores de risco para o desenvolvimento da IUE por afetar nervos, músculos, vasos e o tecido conectivo do assoalho pélvico, estruturas responsáveis pela manutenção da continência. Há evidências de que os danos principalmente nos músculos estriado e liso da uretra são componentes-chave na patogênese da IUE. Neste cenário, a terapia celular tem sido considerada como uma alternativa para o tratamento da IUE com base na capacidade de restaurar o esfíncter uretral lesionado.

Objetivo da Pesquisa:

Objetivo Primário: Melhora da qualidade de vida de mulheres com incontinência urinária de esforço através de terapia com células-tronco adultas, avaliada com questionário específico validado em língua portuguesa (IQol).

Objetivo Secundário: Melhora da incontinência urinária de esforço medida por testes objetivos (exame físico, teste do absorvente e estudo urodinâmico).

Avaliação dos Riscos e Benefícios:

Riscos: No local de retirada das amostras teciduais e sangue periférico, pode haver dor local de leve a moderada, e mais raramente pode haver equimoses ou hematomas, que tendem a desaparecer com o tempo. Mais raramente pode ocorrer infecção e inflamação secundária ao procedimento. Espera-se que no dia da injeção periuretral o local tenha um pouco de dor, ardência ou desconforto, mas bastante leves. Eventualmente pode haver sangramento na urina, mas também muito leve, e de duração curta. Com o tempo, não deve haver maiores problemas. Não é esperada a formação de tumores, por uso exclusivo de células-tronco adultas. As pacientes receberão anestésicos locais ou sedação na ocasião da realização das biópsias.

Benefícios: Melhora da qualidade de vida de pacientes com IUE. Procedimento minimamente invasivo, utilizando material biológico autólogo, evitando-se os riscos inerentes ao uso de material sintético padrão para correção da IUE. Baixo custo.

Comentários e Considerações sobre a Pesquisa:

Trata-se de emenda (E3) ao projeto.

Justificativa para a emenda:

Esta emenda visa regularizar a participação do centro “Hospital Israelita Albert Einstein” como cooparticipante neste projeto de pesquisa no sistema da Plataforma Brasil. O centro HIAE já é coparticipante desde o início desta pesquisa por meio de notificação prévia, uma vez que o centro erroneamente “recusou” o convite do centro colaborador em participar da pesquisa. No entanto, o sistema da Plataforma Brasil nos permite atualmente a re-inclusão do centro via emenda tradicional.

Além deste, esta emenda visa incluir uma versão revisada do TCLE, que inclui a informação da necessidade de coleta de sangue periférico das participantes do estudo que se submeterão à terapia celular com células-tronco derivadas de medula óssea (o sangue autólogo é utilizado para obtenção do cultivo celular), algumas informações adicionais quanto aos efeitos adversos decorrentes da biópsia de tecidos e da anestesia utilizada, bem como a necessidade de preenchimento de diário miccional durante o seguimento do estudo. Foram incluídos na versão revisada do TCLE os seguintes trechos grifados: “Para a retirada das suas células-tronco, será usada uma punção-biópsia com uma seringa ou uma agulha grossa, ou ainda uma pequena incisão de pele da sua coxa de cerca de 3 cm, e a seguir serão levadas para o laboratório. Usaremos anestesia local para esta punção de modo a diminuir o desconforto tal como dor e hematoma (“roxos”). Há pequeno risco de inflamação e infecção no local. No caso da senhora ser sorteada para as células-tronco serem retiradas da gordura ou da medula óssea do seu corpo, há a possibilidade de haver necessidade de fazermos uma sedação leve juntamente com a anestesia localizada, uma vez que o processo usará uma cânula (um tubo mais grosso) para sugar as células de gordura ou da medula. Isto deverá ser feito no hospital, e a senhora terá alta no mesmo dia.
No caso da Sra ser sorteada para o tratamento com células-tronco derivadas de medula óssea, será também necessária a retirada de cerca de 300-500 ml de seu sangue por meio de punção de veia do seu braço, em apropriado banco de sangue humano, e o soro do seu sangue será utilizado para cultivar as células-tronco a serem utilizadas no seu tratamento.”...
... Além destes, a Sra. será convidada a preencher diários miccionais periodicamente na sua casa, onde a sra. registrará os hábitos de sua bexiga, seus hábitos de ingesta líquida, e eventos relacionados com o fucnionamento de sua bexiga.”

Esta emenda visa regularizar a participação do centro “Hospital Israelita Albert Einstein” como cooparticipante neste projeto de pesquisa no sistema da Plataforma Brasil. O centro HIAE já é coparticipante desde o início desta pesquisa por meio de notificação prévia, uma vez que o centro erroneamente “recusou” o convite do centro colaborador em participar da pesquisa. No entanto, o sistema da Plataforma Brasil nos permite atualmente a re-inclusão do centro via emenda tradicional. Além deste, esta emenda visa incluir uma versão revisada do TCLE, que inclui a informação da necessidade de coleta de sangue periférico das participantes do estudo que se

submeterão à terapia celular com células-tronco derivadas de medula óssea (o sangue autólogo é utilizado para obtenção do cultivo celular), algumas informações adicionais quanto aos efeitos adversos decorrentes da biópsia de tecidos e da anestesia utilizada, bem como a necessidade de preenchimento de diário miccional durante o seguimento do estudo.

Considerações sobre os Termos de apresentação obrigatória:

-Documentos apresentados para a emenda:

1- carta justificativa da emenda (Emenda3_CEPUNIFESP_CT.doc);

2- TCLE (TCLE_PF_CT_v4_revisado_Fev2019.docx)

3- TCLE (TCLE_PF_CT_v4_revisado_Fev2019.pdf)

4- DIÁRIO MICCIONAL (DiarioMiccional_CT.pdf)

5- (Projeto_CT_Plataforma_V5_Jan2020.docx)

Recomendações:

Sem recomendações

Conclusões ou Pendências e Lista de Inadequações:

Emenda aprovada

Considerações Finais a critério do CEP:

O presente projeto, seguiu nesta data para análise da CONEP e só tem o seu início autorizado após a aprovação pela mesma.

Este parecer foi elaborado baseado nos documentos abaixo relacionados:

Tipo Documento	Arquivo	Postagem	Autor	Situação
Informações Básicas do Projeto	PB_INFORMAÇÕES_BÁSICAS_1309795_E3.pdf	12/01/2020 15:36:24		Aceito
Outros	DiarioMiccional_CT_V5.docx	12/01/2020 15:33:57	Maria Augusta Tezelli Bortolini	Aceito
Projeto Detalhado / Brochura Investigador	Projeto_CT_Plataforma_V5_Jan2020.docx	12/01/2020 15:32:48	Maria Augusta Tezelli Bortolini	Aceito
Projeto Detalhado	projeto_CT_Plataforma_V5_Jan2020.	12/01/2020	Maria Augusta	Aceito

/ Brochura Investigador	pdf	15:32:00	Tezelli Bortolini	Aceito
Declaração de Pesquisadores	Resposta_Parecer_Emenda3_CEPUNIFESP_CT_12Jan20.pdf	12/01/2020 15:29:26	Maria Augusta Tezelli Bortolini	Aceito
Declaração de Pesquisadores	Resposta_Parecer_Emenda3_CEPUNIFESP_CT_12Jan20.doc	12/01/2020 15:29:04	Maria Augusta Tezelli Bortolini	Aceito
Declaração de Pesquisadores	Emenda3_CEPUNIFESP_CT.doc	06/03/2019 22:45:06	Maria Augusta Tezelli Bortolini	Aceito
TCLE / Termos de Assentimento / Justificativa de Ausência	TCLE_PF_CT_v4_revisado_Fev2019.docx	06/03/2019 22:44:48	Maria Augusta Tezelli Bortolini	Aceito
Outros	DiarioMiccional_CT.pdf	06/03/2019 22:43:53	Maria Augusta Tezelli Bortolini	Aceito
Declaração de Pesquisadores	Emenda3_CEPUNIFESP_CT.pdf	06/03/2019 22:42:38	Maria Augusta Tezelli Bortolini	Aceito
TCLE / Termos de Assentimento / Justificativa de Ausência	TCLE_PF_CT_v4_revisado_Fev2019.pdf	06/03/2019 22:42:03	Maria Augusta Tezelli Bortolini	Aceito
Declaração de Pesquisadores	RecrutamentoTCLE.pdf	06/12/2018 18:39:25	Maria Augusta Tezelli Bortolini	Aceito
TCLE / Termos de Assentimento / Justificativa de Ausência	TCLE.pdf	06/12/2018 18:38:35	Maria Augusta Tezelli Bortolini	Aceito
Declaração de Instituição e Infraestrutura	termoHSM.pdf	06/12/2018 18:02:30	Maria Augusta Tezelli Bortolini	Aceito
Declaração de Pesquisadores	Emenda1Adendo.pdf	06/12/2018 17:53:36	Maria Augusta Tezelli Bortolini	Aceito
Declaração de Instituição e Infraestrutura	FolhaRostoSM.pdf	06/12/2018 17:51:35	Maria Augusta Tezelli Bortolini	Aceito
Declaração de Instituição e Infraestrutura	TermoResponsabilidadeSM.pdf	06/12/2018 17:50:52	Maria Augusta Tezelli Bortolini	Aceito
Declaração de Pesquisadores	Pendencia_Emenda1_CEPUNIFESP_CT.doc	19/06/2018 12:26:44	Maria Augusta Tezelli Bortolini	Aceito
Declaração de Pesquisadores	Pendencia_Emenda1_CEPUNIFESP_CT.pdf	19/06/2018 12:25:55	Maria Augusta Tezelli Bortolini	Aceito
Declaração de Pesquisadores	Pendencia_Emenda1_CT.doc	22/05/2018 15:18:50	Maria Augusta Tezelli Bortolini	Aceito
Declaração de Pesquisadores	Pendencia_Emenda1_CT.pdf	22/05/2018 15:18:12	Maria Augusta Tezelli Bortolini	Aceito

Declaração de Pesquisadores	Emenda1_CT.doc	10/03/2018 12:41:32	Maria Augusta Tezelli Bortolini	Aceito
Declaração de Pesquisadores	Emenda1_CT.pdf	10/03/2018 12:41:08	Maria Augusta Tezelli Bortolini	Aceito
Outros	Coep_CT.pdf	19/01/2017 13:16:54	Maria Augusta Tezelli Bortolini	Aceito
Declaração de Instituição e Infraestrutura	termo_infraestrutura_HIAE.pdf	13/01/2017 17:46:05	Maria Augusta Tezelli Bortolini	Aceito
Declaração de Instituição e Infraestrutura	Declaracao_InfraEstrutura_StemCorp.jpeg	06/12/2016 20:57:54	Maria Augusta Tezelli Bortolini	Aceito
Declaração de Instituição e Infraestrutura	infraestrutura_UNIFESP.jpg	15/11/2016 12:46:23	Maria Augusta Tezelli Bortolini	Aceito
Projeto Detalhado / Brochura Investigador	351lula_tronco_humanos_Castro_v4.pdf	13/10/2016 10:35:55	Maria Augusta Tezelli Bortolini	Aceito
Declaração do Patrocinador	comprovante_Fapesp.pdf	11/10/2016 15:59:45	Maria Augusta Tezelli Bortolini	Aceito
TCLE / Termos de Assentimento / Justificativa de Ausência	TCLE_PF_CT_v4.pdf	11/10/2016 15:56:25	Maria Augusta Tezelli Bortolini	Aceito
Folha de Rosto	FOLHA_ROSTO_CTA_V4.pdf	11/10/2016 15:55:35	Maria Augusta Tezelli Bortolini	Aceito
Outros	Documento_RodrigoCastro2.jpg	24/05/2016 15:07:45	RODRIGO CERQUEIRA DE SOUZA	Aceito
Outros	Documento_RodrigoCastro1.jpg	24/05/2016 15:07:13	RODRIGO CERQUEIRA DE SOUZA	Aceito

Situação do Parecer:
Aprovado

Necessita Apreciação da CONEP:
Sim

SAO PAULO, 31 de Janeiro de 2020

Assinado por:
Miguel Roberto Jorge
(Coordenador(a))
