# Supplementary material for: Stem cell therapy for female stress urinary incontinence: Results, limitations and lessons learned from a pilot clinical study
Source: PLoS One. 2026 Feb 27;21(2):e0342452. doi: 10.1371/journal.pone.0342452 (PMC12948050; doi:10.1371/journal.pone.0342452)
Supplement: S1 Appendix — (ZIP) [file pone.0342452.s004.zip › Supporting Information Files/HIAE_PB_PARECER_CONSUBSTANCIADO_CEP_3907497_Ocultado.pdf]

[redacted] HOSPITAL ISRAELITA ALBERT EINSTEIN-SP [redacted]

PARECER CONSUBSTANCIADO DO CEP

Elaborado pela Instituição Coparticipante

DADOS DO PROJETO DE PESQUISA

Título da Pesquisa: Uso de Células-Tronco Adultas no tratamento de mulheres com incontinência urinária de esforço.

Pesquisador: RODRIGO CERQUEIRA DE SOUZA

Área Temática:

Versão: 1

CAAE: 18150613.7.3005.0071

Instituição Proponente: SOCIEDADE BENEF ISRAELITABRAS HOSPITAL ALBERT EINSTEIN

Patrocinador Principal: FUNDACAO DE AMPARO A PESQUISA DO ESTADO DE SAO PAULO

DADOS DO PARECER

Número do Parecer: 3.907.497

Apresentação do Projeto:

A incontinência urinária de esforço (IUE) é a perda de urina involuntária decorrente de algum esforço físico como pular, correr e tossir. IUE afeta 15-35% das mulheres, interferindo na sua vida social, psicológica e sexual. O parto vaginal e o envelhecimento tecidual são os principais fatores de risco para o desenvolvimento da IUE por afetar nervos, músculos, vasos e o tecido conectivo do assoalho pélvico, estruturas responsáveis pela manutenção da continência. Há evidências de que os danos principalmente nos músculos estriado e liso da uretra são componentes-chave na patogênese da IUE. Neste cenário, a terapia celular tem sido considerada como uma alternativa para o tratamento da IUE com base na capacidade de restaurar o esfíncter uretral lesionado.

Objetivo da Pesquisa:

Objetivo Primário:

Melhora da qualidade de vida de mulheres com incontinência urinária de esforço através de terapia com células-tronco adultas, avaliada com questionário específico validado em língua portuguesa (IQol).

Objetivo Secundário:

Melhora da incontinência urinária de esforço medida por testes objetivos (exame físico, teste do absorvente e estudo urodinâmico).

Avaliação dos Riscos e Benefícios:

Riscos:

No local de retirada das amostras teciduais e sangue periférico, pode haver dor local de leve a moderada, e mais raramente pode haver equimoses ou hematomas, que tendem a desaparecer com o tempo. Mais raramente pode ocorrer infecção e inflamação secundária ao procedimento. Espera-se que no dia da injeção periuretral o local tenha um pouco de dor, ardência ou desconforto, mas bastante leves. Eventualmente pode haver sangramento na urina, mas também muito leve, e de duração curta. Com o tempo, não deve haver maiores problemas. Não é esperada a formação de tumores, por uso exclusivo de células-tronco adultas. As pacientes receberão anestésicos locais ou sedação na ocasião da realização das biópsias.

Benefícios:

Melhora da qualidade de vida de pacientes com IUE. Procedimento minimamente invasivo, utilizando material biológico autólogo, evitando-se os riscos inerentes ao uso de material sintético padrão para correção da IUE. Baixo custo.

Comentários e Considerações sobre a Pesquisa:

Pesquisador incluiu o HIAE como centro coparticipante ao estudo supracitado com a seguinte justificativa:

"Esta emenda visa regularizar a participação do centro “Hospital Israelita Albert Einstein” como coparticipante neste projeto de pesquisa no sistema da Plataforma Brasil. O centro HIAE já é coparticipante desde o início desta pesquisa por meio de notificação prévia, uma vez que o centro erroneamente “recusou” o convite do centro colaborador em participar da pesquisa. No entanto, o sistema da Plataforma Brasil nos permite atualmente a re-inclusão do centro via emenda tradicional. Além deste, esta emenda visa incluir uma versão revisada do TCLE, que inclui a informação da necessidade de coleta

de sangue periférico das participantes do estudo que se submeterão à terapia celular com células-tronco derivadas de medula óssea (o sangue autólogo é utilizado para obtenção do cultivo celular), algumas informações adicionais quanto aos efeitos adversos decorrentes da biópsia de tecidos e da anestesia utilizada, bem como a necessidade de preenchimento de diário miccional durante o seguimento do estudo".

Anteriormente, o HIAE já havia recebido pedido de coparticipação, o projeto havia sido avaliado e aprovado em reunião colegiada, mas por engano o projeto foi reprovado e não foi possível que a a coparticipação fosse refeita, enquanto não houvesse mudança na Plataforma Brasil. A Plataforma

ajustou esta questão, o que permitiu a modificação e assim, pode-se aprovar a coparticipação do estudo no HIAE.

Considerações sobre os Termos de apresentação obrigatória:

Os Termos de apresentação obrigatória encontram-se de acordo com as Resoluções vigentes.

Recomendações:

É atribuição do CEP “acompanhar o desenvolvimento dos projetos, por meio de relatórios semestrais dos pesquisadores e de outras estratégias de monitoramento, de acordo com o risco inerente à pesquisa”. Por isso o/a pesquisador/a responsável deverá encaminhar para o CEP Einstein os Relatórios Parciais a cada seis meses e o Relatório Final de seu projeto, até 30 dias após o seu término.

Relatório Parcial, Final ou de Suspensão de Estudo:
https://www.einstein.br/pesquisa/servicos/comite-etica-em-pesquisa/relatorio-pesquisas-aprovadas

Segundo a Resolução CNS 466/2012 o pesquisador responsável deve prever procedimentos que assegurem a confidencialidade e a privacidade, a proteção da imagem e a não estigmatização dos participantes da pesquisa, garantindo a não utilização das informações em prejuízo das pessoas e/ou das comunidades, inclusive em termos de autoestima, de prestígio e/ou de aspectos econômico-financeiros.

Por favor, se ocorrerem eventos adversos graves, considerar as orientações presentes no link: http://apps.einstein.br/forms/pesquisa/form-adve.html

Se ocorrer um evento relacionado ao procedimento do estudo ou medicação em uso, por favor, preencher o Formulário de Evento Adverso Sério Próprio do CONEP: http://conselho.saude.gov.br/web_comissoes/conep/aquivos/FORMULARIO_EAS_CONEP_2011.doc

Conclusões ou Pendências e Lista de Inadequações:

Após análise, os seguintes documentos foram aprovados:

1-Projeto de Pesquisa (denominado Projeto_CT_Plataforma_V5_Jan2020.docx) - Versão 5 datada de 12 de Janeiro de 2020;

Endereço: Av. Albert Einstein 627 - 2ss
Bairro: Morumbi **CEP:** 05.652-000
UF: SP **Município:** SAO PAULO
Telefone: (11)2151-3729 **Fax:** (11)2151-0273 **E-mail:** cep@einstein.br

2-Termo de Consentimento Livre e Esclarecido (denominado TCLE_PF_CT_v4_revisado_Fev2019.docx)- Versão submetida para análise em 06/03/2019.

Considerações Finais a critério do CEP:

DOCUMENTAÇÃO APROVADA PELO CEP DO HOSPITAL ISRAELITA ALBERT EINSTEIN EM REUNIÃO REALIZADA EM 18/02/2020.

Este parecer foi elaborado baseado nos documentos abaixo relacionados:

Tipo Documento	Arquivo	Postagem	Autor	Situação
Outros	DiarioMiccional_CT_V5.docx	12/01/2020 15:33:57	Maria Augusta Tezelli Bortolini	Aceito
Projeto Detalhado / Brochura Investigador	Projeto_CT_Plataforma_V5_Jan2020.docx	12/01/2020 15:32:48	Maria Augusta Tezelli Bortolini	Aceito
Projeto Detalhado / Brochura Investigador	projeto_CT_Plataforma_V5_Jan2020.pdf	12/01/2020 15:32:00	Maria Augusta Tezelli Bortolini	Aceito
TCLE / Termos de Assentimento / Justificativa de Ausência	TCLE_PF_CT_v4_revisado_Fev2019.docx	06/03/2019 22:44:48	Maria Augusta Tezelli Bortolini	Aceito
Outros	DiarioMiccional_CT.pdf	06/03/2019 22:43:53	Maria Augusta Tezelli Bortolini	Aceito
TCLE / Termos de Assentimento / Justificativa de Ausência	TCLE_PF_CT_v4_revisado_Fev2019.pdf	06/03/2019 22:42:03	Maria Augusta Tezelli Bortolini	Aceito
TCLE / Termos de Assentimento / Justificativa de Ausência	TCLE.pdf	06/12/2018 18:38:35	Maria Augusta Tezelli Bortolini	Aceito
Outros	Coep_CT.pdf	19/01/2017 13:16:54	Maria Augusta Tezelli Bortolini	Aceito
Projeto Detalhado / Brochura Investigador	351lula_tronco_humanos_Castro_v4.pdf	13/10/2016 10:35:55	Maria Augusta Tezelli Bortolini	Aceito
Declaração do Patrocinador	comprovante_Fapesp.pdf	11/10/2016 15:59:45	Maria Augusta Tezelli Bortolini	Aceito
TCLE / Termos de Assentimento / Justificativa de Ausência	TCLE_PF_CT_v4.pdf	11/10/2016 15:56:25	Maria Augusta Tezelli Bortolini	Aceito
Outros	Documento_RodrigoCastro2.jpg	24/05/2016	RODRIGO	Aceito

Outros	Documento_RodrigoCastro2.jpg	15:07:45	CERQUEIRA DE SOUZA	Aceito
Outros	Documento_RodrigoCastro1.jpg	24/05/2016 15:07:13	RODRIGO CERQUEIRA DE SOUZA	Aceito

Situação do Parecer:
Aprovado

Necessita Apreciação da CONEP:
Não

SAO PAULO, 11 de Março de 2020

Assinado por:
Fabio Pires de Souza Santos
(Coordenador(a))

Endereço: Av. Albert Einstein 627 - 2ss
Bairro: Morumbi **CEP:** 05.652-000
UF: SP **Município:** SAO PAULO
Telefone: (11)2151-3729 **Fax:** (11)2151-0273 **E-mail:** cep@einstein.br
