# Supplementary material for: Stem cell therapy for female stress urinary incontinence: Results, limitations and lessons learned from a pilot clinical study
Source: PLoS One. 2026 Feb 27;21(2):e0342452. doi: 10.1371/journal.pone.0342452 (PMC12948050; doi:10.1371/journal.pone.0342452)
Supplement: S1 Appendix — (ZIP) [file pone.0342452.s004.zip › Supporting Information Files/Project_English.pdf]

TITLE:
Use of Human Stem Cells in the Treatment of Women with Stress Urinary Incontinence

ABSTRACT
Stress urinary incontinence (SUI) results from urethral damage, and cell therapy aims to restore the injured urethral sphincter. This study will treat patients with SUI through periurethral injection of autologous bone marrow-derived stem cells, evaluated by clinical examination, urodynamic study, pad test, and quality of life questionnaire after one year of follow-up. We will assess the feasibility, efficacy, and adverse effects of this therapy.

Stress urinary incontinence (SUI) is defined as the involuntary loss of urine during physical exertion such as jumping, running, or coughing. SUI affects 15–35% of women, impacting their social, psychological, and sexual well-being.

Vaginal delivery and tissue aging are the main risk factors for developing SUI, as they affect the nerves, muscles, blood vessels, and connective tissues of the pelvic floor—structures responsible for maintaining urinary continence. There is evidence that damage to the striated and smooth muscles of the urethra plays a key role in the pathogenesis of SUI. In this context, cell therapy using adult stem cells has been considered a potential alternative for treating SUI, based on their ability to regenerate the injured urethral sphincter.

This study aims to treat women with SUI through periurethral injection of autologous striated muscle-derived stem cells. Cure and improvement of incontinence will be assessed through clinical examination, urodynamic testing, and the pad test, as well as with the application of a disease-specific quality of life questionnaire. Pre-treatment data will be compared with those obtained after one year of post-treatment follow-up.

We aim to evaluate the feasibility, efficacy, and possible adverse effects of this therapy in the treatment of SUI in women.

1. Problem Statement

1.1 Stress Urinary Incontinence (SUI)

SUI is defined as any involuntary loss of urine resulting from physical exertion such as jumping, running, sneezing, or coughing[1]. SUI seriously impairs women's quality of life, affecting social, emotional, and economic aspects. The prevalence of this dysfunction in women varies from 12% to 55%[2].

It is believed that SUI is caused by a combination of risk factors, with the most important being the number of pregnancies and the mode of delivery. Vaginal delivery can cause damage to the support and suspension structures of the pelvic floor and urethra, such as the vaginal mucosa, muscles, endopelvic fascia, and ligaments. These structures are mainly composed of smooth and striated muscles, and connective tissue[3],[4]. Some studies describe biochemical and molecular alterations in the tissues of women with SUI compared to continent women, such as a significant reduction in the amount of type I and III collagen around the urethra and in the pubocervical fascia[5]–[7]; a decrease in the ratio of striated muscle to connective tissue, as well as histological reduction of striated muscle fibers; and partial nerve lesions in the urethral striated sphincter[8],[9]. Thus, it is believed that damage to the components of the urethra, with consequent

reduction of intra-urethral pressure, is associated with the pathophysiology of urinary incontinence[10].

Regenerative medicine is a new area of medicine that incorporates various fields of tissue engineering. Among these, the use of stem cells stands out for the development of biological substitutes to restore and maintain the original functions of organs and tissues[11]. As an alternative to surgical treatment, cell therapy using stem cells has been considered promising for the management of SUI due to its potential ability to restore the urethral sphincter.

1.2 Adult Stem Cells (ASCs)

Stem cells (SCs) are undifferentiated cells with the potential to differentiate into specialized cells that are more complex both structurally and functionally when stimulated by an appropriate tissue microenvironment. They serve as sources for repair of tissues and organs in a virtually unlimited way throughout life[12]. They can be of embryonic origin (ESCs), adult origin (ASCs), or induced pluripotent stem cells (iPSCs).

Adult stem cells (ASCs) are found in fully developed tissues or organs: bone marrow, peripheral blood, brain, spinal cord, dental pulp, blood vessels, skin and digestive tract epithelia, cornea, retina, liver, pancreas, and skeletal muscle. Their main functions in a living organism are to maintain tissue homeostasis, that is, to renew it whenever necessary and to replace cells damaged by trauma or disease. This distinguishes them from embryonic stem cells (ESCs), since ASCs seem to differentiate only into more specific cell types restricted to the tissues they belong to. However, some types of ASCs have greater plasticity, meaning the cells can differentiate into cell types that do not share the same embryonic origin[13].

ASCs involve less complex manipulation and greater safety in therapeutic applications, since there is a very low chance of these cells causing tumors compared to ESCs[13].

Experimental and clinical research has been conducted to evaluate the viability, safety, and effects of using ASCs in the repair of damaged urethral tissues. Among the ASCs, the most commonly used and studied in urogynecology are muscle-derived stem cells (MDSCs), adipocyte-derived stem cells (ADSCs), and bone marrow-derived stem cells (BMSCs).

1.3 Use of Muscle-Derived Stem Cells in Urethral Regeneration

Muscle-derived stem cells (MDSCs) mainly differentiate into mesodermal cells (striated muscle, fat, cartilage, and bone). They are obtained by purifying samples of striated muscle and cultured in vitro[14]. The cultured cells can then be injected directly into the area to be treated (damaged tissue) or into the bloodstream, depending on the type of regeneration intended.

Chermansky et al. demonstrated that after periurethral injection, MDSCs integrated into the striated sphincter muscle layers of rat urethras four weeks after urethral injury by cauterization. Moreover, there was regeneration of innervation in the group injected with MDSCs compared to the placebo group that received saline solution, suggesting the multipotency of MDSCs. In the same study, the leak point pressure (intravesical pressure at which urinary leakage occurs during the Valsalva maneuver) was significantly higher in the MDSC group compared to the control group, indicating better urethral function in the treated group. There was no significant difference in leak point pressure in the treated group after 4 and 6 weeks post-injury compared to the uninjured group, suggesting that the urethral function ensuring urinary continence in the group that received MDSCs was similar to that of normal rats, and thus restored[15].

A recent study demonstrated the beneficial effects of cell therapy with MDSCs in monkeys with stress urinary incontinence caused by transection of the pudendal nerves. The authors performed histological analyses and functional urodynamic studies to evaluate the therapy after one year. They observed that the therapy increased the maximum urethral closure pressure in the monkeys, showed a higher proportion of striated muscle area, and a smaller collagen fiber area in the urethras of treated animals compared to untreated controls. Three months after injection, muscle markers (desmin, connexin-43, and α-smooth muscle actin) and Von Willebrand factor were detected, marked with GFP fluorescent protein from the stem cells. Thus, differentiation of MDSCs into muscle tissue and blood vessels was confirmed. The study demonstrates the long-term beneficial outcomes of MDSC therapy for stress urinary incontinence[16].

Carr and colleagues recently published a clinical study involving 38 women with stress urinary incontinence refractory to one year of conventional clinical treatment. In phase 1, participants were randomized into five groups of 4, according to the number of cells they received: 1, 2, 4, 8, and 16 million MDSCs. In phase 2, three groups of 3 participants received 32, 64, and 128 million cells, and in phase 3, another three groups of 3 participants received 16, 32, and 64 million cells injected periurethrally via cystoscopy. Patients could opt for a second injection after 3 months. Evaluation was performed through periodic analysis of voiding diaries, pad tests, and quality of life questionnaires during 12 months of follow-up. Therapy outcomes improved proportionally to the number of cells used. Compared to low-dose groups, a higher proportion of participants in the high-dose groups showed at least a 50% reduction in pad weight during the pad test (88.9%, 8 of 9 vs. 61.5%, 8 of 13), at least a 50% reduction in stress urinary leakage recorded in voiding diaries (77.8%, 7 of 9 vs. 53.3%, 8 of 15), and had up to one leakage episode over 3 days (88.9%, 8 of 9 vs. 33.3%, 5 of 15) at the final one-year follow-up. The reported side effects were few: pain at the muscle biopsy site and injection site, mild and temporary urinary retention, and urinary tract infections[17].

Consistent results regarding the safety and efficacy of cell therapy with local periurethral injections of MDSCs for the treatment of female stress urinary incontinence were observed by Peters and collaborators (2014). Using a similar methodology, the authors evaluated 80 women who received injections into the external urethral sphincter of 10 (n=16), 50 (n=16), 100 (n=24), or 200 (n=24) million autologous MDSCs obtained from quadriceps muscle biopsies. All groups had statistically significant improvement in scores of specific questionnaires evaluating urinary incontinence (UDI-6 and IIQ-7) at 6 and 12 months of follow-up compared to pre-treatment status. Overall, clinical improvements were dose-dependent. Eighty-five percent of patients who received 100 million cells and 77% who received 200 million stem cells showed at least a 50% reduction in stress-related urinary leakage according to voiding diary analysis, an instrument that differentiates types of urinary incontinence. Both doses improved pad test values, with significance reached in the 200 million cell group. However, the authors highlighted that about 50% of patients evaluated had mixed urinary incontinence. Thus, pad test values would not differentiate stress or urgency-related leakage, raising questions about conclusions based solely on the pad test. The authors did not observe serious or persistent adverse effects with therapy at any dose administered[18].

A single randomized study compared autologous MDSC urethral injection with three different surgical techniques for correcting stress urinary incontinence ("Burch, TVT sling, and TOT sling"). The study involved 8 women who received cell therapy, 11 in the Burch group, 26 TVT sling cases, and 41 TOT sling cases. The authors reported slight but not significant improvement in functional parameters in the urodynamic study after MDSC therapy. The results were inferior to those

obtained with surgical procedures, indicating the need for a larger sample size to draw conclusions about the effects of cell therapy[19].

1.4 The Use of Adipose-Derived Stem Cells in Urethral Regeneration

Adipose-derived stem cells (ADSCs) are obtained from white adipose tissue (the most predominant in adults), which is composed of 40-60% mature adipocytes and a stromal fraction, this fraction consisting of fibroblasts, macrophages, mast cells, endothelial cells, hematopoietic cells, and pre-adipocytes. Pre-adipocytes are the precursors known as ADSCs[20]. It has been demonstrated that these cells can differentiate in vitro into adipogenic, myogenic, and osteogenic cells[21]. They may have a role in the treatment of urinary incontinence. Jack et al.[22] demonstrated smooth muscle formation from ADSCs in the detrusor muscle, with contractile and relaxation capacity; meanwhile, Zeng et al.[23] achieved positive results in animal models with rats, improving leak point pressure (LPP) and urethral function after injection of ADSCs into injured urethras.

In clinical practice, there is a report of two cases of patients who received ADSC injections after radical prostatectomy[24]. These patients had moderate stress urinary incontinence (SUI) after surgery for at least 2 years, were not undergoing treatment for SUI, and had no disease recurrence. About 250 ml of adipose tissue was harvested from the anterior abdominal wall, and ADSCs were subsequently isolated. Then, the cells were injected into the paraurethral tissue in the external urethral sphincter region via cystoscopy. One milliliter of pure ADSC solution was applied to the rhabdosphincter (at a depth of 5 mm), and 20 ml of a solution containing intact adipose tissue and ADSCs was injected into the submucosa as a sealing agent. The results were encouraging: progressive improvement of SUI (measured by 24-hour pad test and questionnaire), increased functional urethral length, and increased mean urethral closure pressure. Ultrasound showed that the material injected into the paraurethral region did not disappear after 12 weeks, and Doppler imaging detected blood flow in the area where ADSCs were applied. No side effects were reported.

1.5 The Use of Bone Marrow-Derived Stem Cells in Urethral Regeneration

Bone marrow is a very important source of stem cells. The mesenchymal lineage (mesenchymal stem cells - MSCs) is the most relevant in this field, as it can develop into bone, cartilage, fat, and connective tissue[25–27]. Most studies with these cells have focused on bladder regeneration in animal models, using acellular matrices seeded with MSCs[29–32]. The cells were cultured in vitro and seeded onto acellular matrices (intestinal submucosa), which were implanted into the animals' bladders, resulting in the development of functional smooth muscle from the MSCs.

In addition, there is a study by Kinebuchi et al.[33] that analyzed urethral sphincter regeneration in rats. Seven days after surgically induced injury, the rats were re-operated and received injections of 200,000 to 500,000 MSCs suspended in culture medium into the periurethral tissue. These were compared with animals injected only with culture medium and with animals without urethral injury. Leak point pressure (LPP) values were evaluated for each group before injury and at 1, 4, 6, 8, and 12 weeks after injection (cells or pure culture medium). Rats without injury showed no difference in LPP measurements. Injured rats treated with injections of only culture medium had significantly reduced LPP after 1 week and did not recover up to the 12th week. Rats treated with MSCs showed gradual recovery of LPP, but this was not significantly greater than in the culture medium-only group. Histologically, skeletal muscle proliferation was observed in the

MSC group, but no smooth muscle proliferation. Additionally, increased innervation was observed in the MSC group.

1.4 UNIFESP-EPM Experience

The Urogynecology and Vaginal Surgery Sector of the Department of Gynecology at UNIFESP-EPM has an ongoing research line focused on stem cell therapy (SCT). This research project initially received support from the National Council for Scientific and Technological Development (CNPq): CT-Health/MS/SCTIE/Decit/MCT/CNPq Call No. 17/2008.

We analyzed the effect of administering muscle-derived, adipocyte-derived, and bone marrow-derived stem cells in an animal model of urethral trauma induced by vaginal distension (VD), which mimics urethral injuries resulting from vaginal delivery. We studied and compared the urethras of three groups of rats: control, VD group, and VD group treated with stem cells. The VD technique was developed and standardized in our laboratory, in which a Foley catheter was inserted into the vagina of rats with the balloon maintained inflated with 3 ml of water for intermittent periods of 12 hours. Urethral injuries were confirmed by histological analyses.

Stem cells were obtained from mutated SD-Tg(GFP)2BalRrrrc rats that express the "green fluorescent protein" (GFP) marker in their cells, which are easily detected due to the green color they reflect under fluorescent light (Figure 1).

The cells were cultured and expanded in an appropriate medium. We confirmed the authenticity of the MSCs through a combination of cell differentiation assays, immunophenotyping, and cell cycle phase determination. MSCs, for example, differentiated in vitro into adipocytes, chondroblasts, and osteoblasts, characterizing their potential to mature into cells of the three germ layers (Figure 2).

After completing the cell culture phase, we treated the rats 72 hours after VD injury by injecting the GFP-labeled stem cells into the animals' tail vein. Subsequently, identification of GFP-labeled cells and tissue morphology of the urethra were evaluated on days 7, 14, 21, and 28 post-therapy using histochemical analysis and electron microscopy

We observed that the rats subjected to trauma by vaginal distension (VD) showed disorganization in the histological layers of the urethra, characterized by narrowing and rupture of the smooth and striated muscle fibers.

At 28 days post-trauma, we observed the presence of connective tissue partially replacing the smooth muscle fibers, suggesting a scarring process (Figure 3).

We observed the early presence (7 days after injection) of GFP-labeled cells in all layers of the urethra, demonstrating their migration, integration, and survival within the urethral tissue. Cell therapy improved the structural organization of the urethras and tissue remodeling compared to untreated traumatized rats. After 4 weeks of treatment, we observed equal proportions of smooth muscle and connective tissue in the urethra, as well as extensive recovery of striated muscle, both similar to the structural pattern of control rats (Figure 3).

Our results with the use of bone marrow-derived stem cells (BMSC), muscle-derived stem cells (MDSC), and adipose-derived stem cells (ADSC), and their effects on the urethra were very similar.

Immunohistochemical analyses using desmin marker (a marker of smooth and striated muscle) demonstrated improved structural organization and thickness of the urethral muscle layers in

rats treated with MDSC compared to untreated rats after trauma (Figure 4). These findings suggest the beneficial effect of MDSC therapy on urethral regeneration.

In order to understand the effects of cell therapy with muscle-derived stem cells (MDSC) at the molecular level and associate them with histological findings, we performed gene and protein expression studies of the urethras from rats. We compared the control group without DV trauma, the DV group without cell therapy 28 days after trauma, and the group that underwent DV and was treated with MDSC. Markers of smooth and striated muscle, cell proliferation markers, and growth factors were studied.

Regarding gene expression, we observed an increase in the cell proliferation gene Ki67 and collagen genes 1 and 3 in the group treated with MDSC; meanwhile, the heavy chain genes of smooth and striated muscle, as well as the neural growth factor NGF, were elevated in the trauma groups and practically returned to baseline levels after treatment. The vascular growth factor VEGF did not show alteration at different time points (Figure 5).

We focused our protein analysis on muscle components: heavy chain myosins of smooth muscle (MHY11) and striated muscle (MHY1), and observed a significant increase in protein expression in the groups of rats that received therapy with MDSC, demonstrating the regenerative effects of the urethral therapy (Figure 6).

In addition, this study demonstrates the safety of therapies with adult stem cells (ASCs). We did not observe any cases of rejection, tumor formation, or other complications in our trials.

Note: see Figures in the original version of the protocol.

2. Expected Results

Given the experimental findings that suggest significant recovery of urethral components and structure, cell therapy with ASCs could be viable and useful for treating women with stress urinary incontinence (SUI), as it would aid the regeneration process of damaged urethras, such as those injured by vaginal birth trauma.

However, the effects of cell therapy in clinical practice have not yet been sufficiently demonstrated, and additional clinical studies are needed to confirm its viability and potential benefits for incontinent women. Therefore, we are interested in continuing research in this field.

We expect to demonstrate that therapy with ASCs derived from skeletal muscle (MDSCs), bone marrow (BMSCs), and adipocytes (ADSCs) is viable for treating SUI and improves quality of life and urinary leakage in affected women.

3. Scientific and Technological Challenges

3.1 General Objective:

The objective of this study is to evaluate the viability and effects of urethral injection therapy with bone marrow-derived stem cells (BMSCs), muscle-derived stem cells (MDSCs), and adipose-derived stem cells (ADSCs) in women with stress urinary incontinence (SUI).

3.2 Specific Objectives:

- Isolation and in vitro cultivation of autologous MDSCs, BMSCs, and ADSCs obtained from urogynecological patients with SUI;

- Analysis of the viability of intra-urethral cell therapy in women with SUI regarding methodology and potential adverse effects;
- Evaluation of women after cell therapy using objective parameters to assess urinary leakage: stress test, pad test, and urethral closure pressure assessment by urodynamic study;
- Evaluation of women after cell therapy using subjective parameters through the application of a quality of life questionnaire specific for urinary incontinence (I-QOL).

4. Methodology

4.1 Study Design and Population:

Prospective randomized clinical study involving 45 patients with SUI who will receive peri-urethral injection of 5 ml solution containing 100 million autologous ASCs (derived from muscle, bone marrow, or adipose tissue).

4.2 Study Location:

This is a joint study between the Urogynecology Sector, General Gynecology Discipline, Department of Gynecology at the Federal University of São Paulo (UNIFESP), and the Hematology and Hemotherapy Sector of the Hospital Israelita Albert Einstein Hospital (HIAE), São Paulo, SP, and the private company Stemcorp, São Paulo, SP.

4.3 Participants

Inclusion Criteria

1. Patients with moderate stress urinary incontinence (SUI), primary and refractory to previous clinical treatments (behavioral therapy and physiotherapy), confirmed by physical examination (stress test), I-QOL questionnaire, cystometry (urodynamic study), and pad test (between 10 and 50 grams).

Exclusion Criteria

1. Patients with anterior vaginal wall or uterine prolapse greater than stage 2 (POP-Q classification);
2. Patients previously submitted to surgical procedures for SUI correction;
3. Patients with genital malformations that prevent injection in the mid-urethral site;
4. Patients with previous pelvic radiotherapy;
5. Patients who during clinical and/or urodynamic evaluation present other diagnoses besides SUI (mixed incontinence, urge incontinence, detrusor overactivity, paradoxical incontinence, neurogenic bladder, urinary obstruction);
6. Patients who do not consent to participate in any stage of the study or who are unable to read, write, and understand the study;
7. Patients under 18 years of age.

4.4 Location of Procedures

Patients will be recruited and evaluated (pre- and post-treatment) at the Urogynecology Outpatient Clinic of UNIFESP. Procedures for bone marrow biopsy, cell isolation, and cultivation will be performed at the human cell therapy laboratory in the Hematology and Hemotherapy Sector of Hospital Israelita Albert Einstein, São Paulo. Procedures for adipose tissue and biceps muscle biopsy will be performed in the surgical center of the Hospital São Paulo, and the cell isolation and culture procedures will be carried out at the Stemcorp Cell Therapy Laboratory, São Paulo.

4.5 Pre- and Post-Therapy Diagnostic Tests

a) Clinical Examination – Stress Test:

To diagnose urinary incontinence, a stress test will be performed in which urine leakage is evidenced when the patient performs strain maneuvers with a comfortably full bladder.

b) Pad Test:

The pad test will be used to quantify urine loss. With an empty bladder, 250 ml of distilled water will be instilled into the bladder. A pre-weighed pad will then be placed in the perineal region, and the patient will perform the following stress maneuvers: coughing, jumping, squatting, and abdominal muscle contraction (Valsalva), ten times each. Additionally, they will climb five steps ten consecutive times, wash their hands for one minute, and walk for twenty minutes. The total test duration will not exceed one hour. Afterwards, the pad will be weighed; a weight difference greater than 2 grams will be considered a positive test. [34]

c) Cystometry:

The urodynamic test will be conducted using the Urosystem PL-2400 Polimed four-channel device. Cystometry will be performed with the patient in a semi-seated position using a two-way urethral catheter—one channel for bladder filling and the other for intravesical pressure measurement. Room-temperature distilled water will be infused at a rate of 40 ml/min. Abdominal pressure will be monitored using a rectal balloon. During the filling phase, when 200 ml have been infused, the patient will perform a Valsalva maneuver to determine the intravesical pressure at the moment of urinary leakage through the urethra. The test will be considered positive whenever urine leakage occurs, and the leak point pressure (LPP) will be recorded.

d) Quality of Life Questionnaires:

We will use the “Incontinence Quality of Life Questionnaire (I-QoL)” to assess quality of life. The I-QoL consists of 22 questions organized into three domains that assess behavioral limitations, psychosocial impact, and social embarrassment. All responses are assigned values ranging from 1 to 5, therefore allowing for quality of life to be measured on a scale from 0 to 100. The lower the score, the worse the quality of life. [35]

e) Safety Assessment of Cell Therapy:

Participants will have unrestricted access to the research center and investigators during and after the study, in order to maintain close safety monitoring. During postoperative follow-up visits, the presence of adverse effects will be assessed, such as: irritative symptoms (increased urinary frequency, dysuria, suprapubic pain, urgency, and urge incontinence); signs suggestive of urinary tract infection; obstructive symptoms (incomplete bladder emptying sensation, decreased urinary stream, need to strain during urination); infection or fluid collection at the puncture site; vulvovaginal itching or infection; hematuria. If a urinary tract infection (UTI) is suspected,

urinalysis and urine culture will be performed for diagnostic confirmation. If partial or total urinary obstruction is suspected, free uroflowmetry and post-void residual measurement via ultrasound will be performed. Partial obstruction will be defined as post-void residual > 150 ml; in such cases, expectant management with behavioral measures to aid bladder emptying will be adopted. In cases of total obstruction after periurethral injection, the patient will be managed with indwelling catheterization and weekly follow-up visits to assess her voiding pattern. The catheter will be removed when the post-void residual is < 150 ml.

We do not anticipate serious adverse effects from adult stem cell therapy, which has been proven not to carry a risk of tumor formation. Furthermore, previous studies on cell therapy for SUI have demonstrated the safety of this treatment. [16–18] As described in the following sections, appropriate methods for the identification and characterization of stem cells will be employed.

f) Management of Study Step Failures:

In cases where it is not possible to carry out the laboratory procedures, or the procedures for harvesting or injecting the stem cells due to technical difficulties or patient discomfort, the participant's involvement in the study will be discontinued. In the event of adverse effects, the patient will be promptly assisted by the research team and will have full access to the outpatient and hospital structure (Hospital São Paulo), if necessary.

4.6 Method of Preparation of BM-MSCs (Bone Marrow-Derived Mesenchymal Stem Cells)
I – Isolation of Bone Marrow-Derived Stem Cells from Primary Culture of Bone Marrow Biopsy

Collection:
Using appropriate sterile material and aseptic technique, and under local anesthesia with 2% lidocaine, bone marrow aspiration will be performed from the anterior and posterior iliac crests of the patients, depending on individual assessment. Approximately 50 ml of bone marrow solution will be collected.

Processing and Cell Culture:

All processing steps will be carried out under sterile conditions in a specific laminar flow hood. The bone marrow material will be cultured in Dulbecco's Modified Eagle Medium (DMEM). Using Ficoll-Paque, the solution will be centrifuged at 500 rpm for 30 minutes at 22°C. The cell layer at the Ficoll-solution interface will then be collected, resuspended in phosphate-buffered saline (PBS), and washed three times to remove any Ficoll residue. The cells will be suspended in DMEM supplemented with fetal bovine serum, penicillin, and streptomycin, and placed in an incubator for 24 hours to allow adherence to the plastic surface.

The DMEM solution with the resuspended cells will be distributed in 75 cm^2 culture flasks and maintained in a humidified incubator at 37°C with 5% CO_2. Under sterile conditions, after 24 hours of isolation and cell adhesion to the culture flask, the medium will be removed by aspiration using a sterile fine-tipped Pasteur pipette. The flask will then be washed three times with PBS containing penicillin and streptomycin at 37°C, and fresh DMEM will be added. To maintain the culture, the medium will be renewed every 48 hours.

When the cells adhered to the flask reach approximately 80% confluence, they will be passaged through trypsinization. This procedure aims to detach the cells from the flask and transfer them to another vessel and/or use them in subsequent procedures (cells will be used between the second and third passages). For trypsinization, the culture medium will be removed by aspiration and the flask washed with PBS supplemented with 0.5M EDTA. After removing the PBS solution

by aspiration, a solution of 0.10% trypsin and 0.02% EDTA will be added in a volume equal to that of the previous medium, and incubated for 3 minutes. Trypsin will then be neutralized using DMEM. The resulting cell suspension will be centrifuged at 300 rpm for 6 minutes, the supernatant was removed by aspiration, and the cells were resuspended in DMEM. The cells were maintained in a humidified incubator at 37°C and 5% CO_2 until they once again reached approximately 80% confluence.

II – Isolation of Myoblasts and Fibroblasts from Primary Culture of Muscle Biopsy Collection: Under local anesthesia, samples of the *quadriceps femoris muscle* (approximately 0.3 cm^3) will be collected by percutaneous needle biopsy and transported to the laboratory in transport medium containing 15 mL of 1X PBS with 10 µg/mL amphotericin B and 4% penicillin/streptomycin (10,000 IU, 10 mg/mL). The transport will be conducted in a thermal container with ice at a temperature of 4±2°C. Processing must occur within 24 hours of collection.

Processing: All processing steps will be performed under sterile conditions in a laminar flow hood. In summary, the tissue fragment will undergo enzymatic digestion using type II and IV collagenases to obtain the two cell types. The material will be centrifuged at 400g for 5 minutes, and the resulting cell pellet will be resuspended in the appropriate culture media.

Cell Culture: If cell expansion or increased yield is necessary, the cells will be cultured in flasks treated with Cellstart-CTSTM (Invitrogen). Fibroblasts will be cultured in DMEM:F12 medium with 20% fetal bovine serum (FBS), while myocytes will be cultured in a chemically defined MCDB 153 medium. Both media will be supplemented with 1% glutamine, 1% amphotericin B (250 µg/mL solution), and 1% penicillin/streptomycin (10,000 IU, 10 mg/mL). The cells will be either cryopreserved or used between the 3rd and 5th passages.

III – Isolation of Adipocytes from Primary Culture of Adipose Tissue Biopsy Collection: A lipoaspirate sample will be collected after asepsis and antisepsis, followed by infiltration of 50 cc of 0.25% lidocaine solution with epinephrine 1:500,000 into the abdominal wall region. The material will be aspirated using syringes equipped with 3 mm diameter cannulas. The samples will be immediately transferred to a sterile 100 mL culture flask containing 50 mL of Hank's Balanced Salt Solution (HBSS) supplemented with 100 U/mL penicillin and 100 µg/mL streptomycin.

Processing: In a sterile environment, under a laminar flow hood, the tissue fragments will be washed in six beakers containing 20 mL of HBSS solution using sterile forceps and the fragments will be transferred to a 100 mm^2 Petri dish, and the adipose tissue will be dissected and cut into small fragments of approximately 0.5 mm^3 using iris scissors. The resulting fragments will be transferred to a sterile 100 mL glass flask and incubated with type II collagenase (Sigma Chemical Company, St. Louis, MO, USA), at a concentration of 1 mg/mL in HBSS, using a ratio of 5 parts collagenase to 1 part tissue. The mixture will be agitated for 10 minutes at 37°C. The resulting solution will be filtered through a 250 µm nylon mesh. The filtrate will be transferred into sterile 50 mL conical tubes and centrifuged for 10 minutes at 300 g at room temperature.

Cell Culture: The pellet will be transferred to a 50 mL conical tube and washed with 20 mL of Dulbecco's Modified Eagle Medium (DMEM)/Ham's F-12 Nutrient Mixture (Sigma Chemical Company, St. Louis, MO, USA) supplemented with 10% fetal bovine serum (FBS), 0.1% bovine serum albumin (BSA), and 100 U/mL of penicillin and streptomycin.

IV – Characterization, Quality Control, and Biosafety of Cell Populations
To ensure the quality of the samples and cultured cells, the following will be performed: flow cytometry for immunophenotyping and cell cycle analysis, as well as differentiation assays (osteogenic, chondrogenic, and adipogenic) to characterize stem cells. For biosafety control of the cultures, karyotyping and both aerobic and anaerobic blood cultures will be performed.

The cell populations obtained from each patient's samples will be characterized using the following markers: CD29, CD44, CD73, CD90, CD105, CD166, CD146, CD14, CD19, CD45, HLA-DR, CD34, CD11B, CD31, CD106, CD4, CD5, CD9, CD10, CD13, CD36, CD38, CD45, CD49, CD56, CD133, STRO-1, OCT4, SOX-2, Nanog, SSEA-4, HLA-ABC, HLA-DR, CXCR-4, LIN, MHC-I, MHC-II, vimentin, smooth muscle actin, Flk-1, Sca-1, BCL, and vWf.

4.7 Logistics, Physical and Laboratory Infrastructure, Biorepository

At UNIFESP, the participants will undergo clinical evaluations and pre-treatment testing. Subsequently, they will be referred to the Hematology and Hemotherapy Department at HIAE, where bone marrow biopsies and peripheral blood collection will be performed. The extraction and cultivation of stem cells will be conducted in the human cell therapy laboratory within the Hematology and Hemotherapy Department at HIAE. At the time of treatment, the injection of autologous stem cells will be carried out at the Urogynecology Department of UNIFESP.

Similarly, the skeletal muscle and adipose tissue biopsies obtained at UNIFESP (Hospital São Paulo) will be sent to the Stemcorp cell therapy laboratory for stem cell isolation and cultivation.

The transport of biological material between research centers will comply with Anvisa (Brazilian Health Regulatory Agency) regulations, ensuring proper packaging of the samples.

This research involves the creation of a biorepository located at HIAE and at Stemcorp, specifically tied to this project. The biorepository will be deactivated upon the completion of the study. Biological products derived from these samples during and after the study will be incinerated in accordance with biospecimen disposal regulations.

4.8 Database

The participants' identification, along with their clinical, epidemiological, and diagnostic test data, will be assigned sequential codes. This information will be compiled in a virtual database accessible to the research centers, where laboratory analysis results will also be added. These data will also be stored in a repository at UNIFESP, with access and updates managed by the primary research center.

4.9 Periurethral Injection of the Cell Preparation

Transparent syringes will contain 5 mL of solution (stem cells + human serum) to be injected into the mid-urethral region, at the level of the urethral sphincter. Under urethroscopic guidance, a needle will be introduced through the device to the appropriate location and inserted approximately 3 mm into the mucosa. A total of 2.5 mL will be injected at the 3 o’clock position and 2.5 mL at the 9 o’clock position, without urethral obstruction. The procedure will be performed on an outpatient basis, and there will be no need for anesthesia or analgesia.

5. Post-Procedure Evaluation

The patient will be observed for two hours following the procedure. Follow-up visits will occur at 7, 30, 60, and 180 days for clinical evaluation after treatment.

One year after the procedure, patients will complete another round of questionnaires, pad test, and cystometry with leak point pressure evaluation. These data will be disclosed only after the final assessment of the entire study population.

The **primary outcome** is improvement in quality of life, which will be assessed using the I-QoL questionnaire before and after treatment. Patients scoring between 0 (complete cure) and 20 (inclusive) on the post-treatment I-QoL will be considered improved. Patients with scores above 20 will be considered not improved.

In addition, patients will be considered cured if they show no signs of urinary leakage during the clinical examination, cystometry, or pad test.

6. Statistical Method

Data processing and analysis: The SAS software version 8.2 will be used to verify data consistency and perform statistical analysis.

Descriptive analysis: Descriptive analysis will be used to characterize the study sample. For categorical variables, absolute and relative frequency distributions will be used. For continuous variables, measures of central tendency (mean and median) and variability (standard deviation, upper and lower limits) will be applied.

Inferential analysis: Comparisons between pre- and post-treatment parameters will be analyzed using the *Wilcoxon rank-sum test* and *Student's t-test* for non-parametric and parametric variables, respectively. The *Chi-square test* and *Fisher's exact test* will be used to assess the homogeneity of categorical variables (race, hormonal status, and previous surgeries for stress urinary incontinence), as well as the absence of urinary leakage on urodynamic testing after the intervention. The *Mann-Whitney test* will be applied to assess the homogeneity and possible differences in medians of continuous variables between pre- and post-treatment groups that measure treatment success measured by the "I-QoL" questionnaire and pad test) will be assessed. For all statistical tests, a significance level of 0.05 or 5% ($\alpha \leq 0.05$) will be adopted as the threshold for rejecting the null hypothesis.

7 Timeline

The estimated duration of the study is 2 years.

8. Evaluation and Dissemination

The results obtained will be presented at national and international conferences in the fields of Gynecology, Urogynecology, Cellular and Molecular Biology, and Regenerative Medicine. We aim to publish the manuscripts in journals with an impact factor greater than 3.0.

9. Other Support

This study project receives partial support from FAPESP.

10. References

1. Haylen BT, Ridder D, Freeman RM, Swift SE, Berghmans B, Lee J et al. An International Urogynecological Association (IUGA)/International Continence Society (ICS) Joint Report on the Terminology for Female Pelvic Floor Dysfunction. Neurourol Urodyn 2010;29:4–20

2. Diokno AC, Brock BM, Brown HB, Herzog AR. Prevalence of urinary incontinence and other urologic symptoms in the non-institutionalized elderly. J Urol 1986;136:1022-5

3. Rortveit G, Daltveit AK, Hannestad YS, Hunskaar S. Norwegian EPINCONT Study. Urinary incontinence after vaginal delivery or cesarean section. N Engl J Med 2003;6: 348:900-7

4. DeLancey JO. Structural support of the urethra as it relates to stress urinary incontinence: the hammock hypothesis. Am J Obst Gynecol 1994;170:1713-23

5. Liapis A, Bakas P, Pafiti A, Hassiakos D, Frangos-Plemenos M, Creatsas G. Changes in the quantity of collagen type I in women with genuine stress incontinence. Urol Res 2000;28(5):323-6

6. Liapis A, Bakas P, Pafiti A, Frangos-Plemenos M, Arnoyannaki N, Creatsas G. Changes of collagen type III in female patients with genuine stress incontinence and pelvic floor prolapse. Eur J Obstet Gynecol Reprod Biol 2001;97(1):76-9

7. Goepel C, Hefler L, Methfessel HD, Koelbl H. Periurethral connective tissue status of postmenopausal women with genital prolapse with and without stress incontinence. Acta Obstet Gynecol Scand 2003;82(7):659-64

8. Hale DS, Benson JT, Brubaker L, Heidkamp MC, Russell B. Histologic analysis of needle biopsy of urethral sphincter from women with normal and stress incontinence with comparison of electromyographic findings. Am J Obstet Gynecol 1999;180(2 Pt 1):342-8

9. Smith AR, Hosker GL, Warrell DW. The role of pudendal nerve damage in the aetiology of genuine stress incontinence in women. Br J Obstet Gynaecol 1989;96(1):29-32

10. Rud T, Andersson KE, Asmussen M, Hunting A, Ulmsten U. Factors maintaining the intraurethral pressure in women. Invest Urol 1980;17(4):343-7

11. Mason C, Dunnill P. A brief definition of regenerative medicine. Regen Med 2008;3(1):1-5

12. Ramalho-Santos M, Yoon S, Matsuzaki Y, Mulligan RC, Melton DA. "Stemness": transcriptional profiling of embryonic and adult stem cells. Science. 2002; 18;298(5593):597-600

13. Hart ML, Neumayer KM, Vaegler M, Daum L, Amend B, Sievert KD, Di

Giovanni S, Kraushaar U, Guenther E, Stenzl A, Aicher WK. Cellbased therapy for the deficient urinary sphincter. Curr Urol Rep 2013;14(5):476-87

14. Williams JT. Cells isolated from adult human skeletal muscle capable of differentiating into multiple mesodermal phenotypes. Am Surg 1999; 65: 22–26

15. Chermansky CJ, Tarin T, Kwon DD, Jankowski RJ, Cannon TW, de Groat WC, Huard J, Chancellor MB. Intraurethral muscle-derived cell injections increase leak point pressure in a rat model of intrinsic sphincter deficiency. Urology 2004; 63:780–785

16. Badra S, Andersson KE, Dean A, Mourad S, Williams JK. Long-term structural and functional effects of autologous muscle precursor cell therapy in a nonhuman primate model of urinary sphincter deficiency. J Urol 2013;190(5):1938-45

17. Carr LK, Robert M, Kultgen PL, Herschorn S, Birch C, Murphy M, Chancellor MB. Autologous muscle derived cell therapy for stress urinary incontinence: a prospective, dose ranging study. J Urol 2013;189(2):595-601

18. Peters KM, Dmochowski RR, Carr LK, Robert M, Kaufman MR, Sirls LT et al. Autologous Muscle Derived Cells for Treatment of Stress Urinary Incontinence in Women. J Urol 2014;pii: S0022-5347(14)00302-4

19. Surcel C, Savu C, Chibelean C, Iordache A, Mirvald C, Sinescu I. Comparative analysis of different surgical procedures for female stress urinary incontinence. Is stem cell implantation the future? Rom J Morphol Embryol 2012;53(1):151-4

20. Roche R, Festy F, Fritel X. Stem cells for stress urinary incontinence: the adipose promise. 2010; J Cell Mol Med. 14 (1-2): 135-42.

21. Zuk PA, Zhu M, Mizuno H, Huang J, Futrell JW, Katz AJ . Multilineage cells from human adipose tissue: implications for cell based therapies. Tissue Eng. 2001; 7: 211-28.

22. Jack GS, Zhang R, Lee M. Urinary bladder smooth muscle engineered from adipose stem cell and a three dimensional synthetic composite. Biomaterials. 2009; 30: 3259-70.

23. Zeng X, Jack GS, Zhang R, et al. Treatment of SUI using adipose derived stem cells: restoration of urethral function. J Urol. 2006; 175:291.

24. Yamamoto T, Gotoh M, Hattori R, Toriyama K, Kamei Y, Iwaguro H, et al. Periurethral injection of autologous adipose derived stem cells for the treatment of stress urinary incontinence in patients undergoing radical prostatectomy: Report of two initial cases. Int J Urol. 2010; 17:75–82.

25. Becker AJ, McCullough EA, Till JE. Cytological demonstration of the clonal nature of spleen colonies derived from transplanted mouse narrow cells. Nature. 1963; 197: 452-4.

26. Friedenstein AJ, Chailakhjan RK, Lalykina KS. The development of fibroblast colonies in monolayer cultures of guinea-pig bone marrow and spleen cells. Cell Tissue Kinet. 1970; 3: 393-403.

27. Owen M. Marrow derived stromal stem cells. J Cell Science Supp. 1988; 10: 63-76.

28. Chung SY, Krivorov NP, Rausei V, Thomas L, Frantzen M, Landsittel D. Bladder reconstitution with bone marrow derived stem cells seeded on small intestinal submucosa improves morphological and molecular composition. J Urol. 2005; 174:353–359.

29. Zhang Y, Lin HK, Frimberger D, Epstein RB, Kropp BP. Growth of bone marrow stromal cells on small intestinal submucosa: an alternative cell source for tissue engineered bladder. BJU Int. 2005; 96:1120–1125

30. Zhang Y, Frimberger D, Cheng EY, Lin HK, Kropp BP. Challenges in a larger bladder replacement with cell-seeed and unseeded small intestinal submucosa grafts in a subtotal cystectomy model. BJU Int. 2006; 98:1100–1105.

31. Shukla D, Box GN, Edwards EA, Tyson DR. Bone marrow stem cells for urologic tissue engineering. World J Urol. 2008; 26:341–349.

32. Kinebuchi Y, Aizawa N, Imamura T, Ishizuka O, Igawa Y, Nishizawa O. Autologous bone marrow derived mesenchymal stem cell transplantation into injured rat urethral sphincter. Int J Urol. 2010; 17(4): 359-68.

33. Lose G, Rosenkilde P, Gammelgaard J, Schroeder T. Padweighing test performed with standardized bladder volume. Urology 1988;32(1):78-80

34. Patrick DL, Martin ML, Bushnell DM, Marquis P, Andrejasich CM, Buesching DP. Cultural adaptation of a quality-of-life measure for urinary incontinence. Eur Urol 1999;36(5):427-35
