# Supplementary material for: Stem cell therapy for female stress urinary incontinence: Results, limitations and lessons learned from a pilot clinical study
Source: PLoS One. 2026 Feb 27;21(2):e0342452. doi: 10.1371/journal.pone.0342452 (PMC12948050; doi:10.1371/journal.pone.0342452)
Supplement: S1 Appendix — (ZIP) [file pone.0342452.s004.zip › Supporting Information Files/Project_final.pdf]

TÍTULO: Uso de células-tronco humanas no tratamento de mulheres com incontinência urinária de esforço.

RESUMO

A incontinência urinária de esforço (IUE) é consequência de danos à uretra, sendo que a terapia celular almeja restaurar o esfíncter uretral lesado. O estudo tratará pacientes com IUE com injeção peri-uretral de células-tronco autólogas derivadas de medula óssea, avaliadas por exame clínico, estudo urodinâmico, teste do absorvente e questionário de qualidade de vida, após 1 ano de seguimento. Avaliaremos a viabilidade, eficácia e efeitos adversos desta terapêutica.

A incontinência urinária de esforço (IUE) é a perda de urina involuntária decorrente de algum esforço físico como pular, correr e tossir. A IUE afeta 15-35% das mulheres, interferindo na sua vida social, psicológica e sexual.

O parto vaginal e o envelhecimento tecidual são os principais fatores de risco para o desenvolvimento da IUE por afetar nervos, músculos, vasos e o tecido conectivo do assoalho pélvico, estruturas responsáveis pela manutenção da continência urinária. Há evidências de que os danos principalmente nos músculos estriado e liso da uretra são componentes-chave na patogênese da IUE. Neste cenário, a terapia celular com células-tronco adultas tem sido considerada como uma potencial alternativa para o tratamento da IUE com base na capacidade de restaurar o esfíncter uretral lesionado.

Este estudo visa tratar as pacientes com IUE com injeção peri-uretral de células-tronco autólogas derivadas de músculo estriado. A cura e melhora da incontinência serão avaliados por exame clínico, estudo urodinâmico e teste do absorvente, bem como com a aplicação de questionário de qualidade de vida específico. Os dados pré-tratamento serão comparados aos obtidos após um ano de seguimento pós-terapia.

Esperamos avaliar a viabilidade, eficácia e eventuais efeitos adversos desta terapêutica no tratamento da IUE nas mulheres.

TITLE: Use of human stem cells in the treatment of female stress urinary incontinence.

ABSTRACT

Stress urinary incontinence (SUI) is the involuntary loss of urine due to physical exertion such as jumping, running and coughing. SUI affects 15-35 % of women, impacting their social, psychological and sexual life.

Vaginal delivery and tissue aging are the main risk factors for the development of SUI by affecting nerves, muscles, vessels and connective tissue of the pelvic floor structures responsible for maintaining urinary continence. There is evidence that the damage mainly in striated and smooth muscles of the urethra are key components in the pathogenesis of SUI. In this scenario, cell therapy with adult stem cells has been considered as a potential alternative for the treatment of SUI based on the ability to restore the injured urethral sphincter.

This study aims to treat patients with SUI with periurethral injection of autologous stem cells derived from striated muscle. The cure and improvement of incontinence will be evaluated by clinical examination, urodynamics and pad tests, as well as the implementation of specific quality of life questionnaire. Baseline data will be compared with those obtained after therapy in one year of follow-up.

We hope to evaluate the feasibility, efficacy and possible adverse effects of the therapy in the treatment of women with SUI.

1. Enunciado do problema

1.1 Incontinência urinária de esforço (IUE)

A IUE é definida como toda perda de urina decorrente de algum esforço físico como pular, correr, espirrar e tossir[1]. A IUE traz sérios prejuízos à qualidade de vida da mulher, afetando os aspectos sociais, emocionais e econômicos. A prevalência da disfunção nas mulheres varia de 12% a 55%[2].

Acredita-se que a IUE seja causada pela associação de fatores de risco, sendo os mais importantes, o número de gestações e a via de parto. O parto vaginal pode provocar danos nas estruturas de suporte e sustentação do assoalho pélvico e da uretra, tais como a mucosa vaginal, músculos, fáscia endopélvica e ligamentos. Estas estruturas são formadas principalmente pelas musculaturas lisa e estriada, e pelo tecido conjuntivo[3,4]. Alguns estudos descrevem alterações bioquímicas e moleculares nos tecidos de mulheres com IUE quando comparadas às mulheres continentes, tais como: a significativa redução na quantidade de colágeno tipos I e III ao redor da uretra e na fáscia pubocervical[5-7]; diminuição na relação músculo estriado/tecido conjuntivo, bem como a redução histológica das fibras musculares estriadas; e lesões nervosas parciais no esfíncter estriado uretral[8,9]. Desta forma, acredita-se que danos aos componentes da uretra, com conseqüente redução da pressão intra-uretral, estão associados à fisiopatologia da IU[10].

A medicina regenerativa é uma nova área da Medicina que agrega diversos campos da engenharia tecidual. Dentre estes, destaca-se a utilização de células-tronco para o desenvolvimento de substitutos biológicos para restaurar e manter as funções originais de órgãos e tecidos[11]. Em substituição ao tratamento cirúrgico, a terapia celular com o uso de células-tronco tem sido considerada promissora para o manejo da IUE devido à capacidade potencial de restaurar o esfíncter uretral.

1.2 Células –Tronco Adultas (CTA)

As células-tronco (CT) são as indiferenciadas com potencial para se diferenciar em células especializadas mais complexas, estrutural e funcionalmente, quando estimuladas por adequado microambiente tecidual. São fontes de reparo de tecidos e órgãos de maneira praticamente ilimitada, durante toda a vida[12]. Podem ser de origem embrionária (CTE), adulta (CTA) ou com pluripotência induzida (CTPI).

As CTA são encontradas em tecidos ou órgãos completamente formados: medula óssea, sangue periférico, cérebro, medula espinhal, polpa do dente, vasos sangüíneos, epitélios da pele e do sistema digestório, córnea, retina, fígado, pâncreas e músculo esquelético. Suas principais funções em um organismo vivo são manter a homeostase do tecido, isto é, renová-lo sempre que necessário e repor as células lesadas seja por trauma ou doença. Isto as difere das CTE, uma vez que as CTA parecem se diferenciar somente em tipos celulares mais específicos restritos aos tecidos a que pertencem. Entretanto, alguns tipos de CTA têm maior plasticidade, isto é, as células podem se diferenciar em tipos celulares que não têm a mesma origem embrionária[13].

As CTA apresentam manipulação menos trabalhosa e maior segurança em aplicações terapêuticas, uma vez que há pouquíssimas chances destas células originarem tumores quando comparadas com as CTE[13].

Pesquisas experimentais e clínicas têm sido realizadas para avaliar a viabilidade, segurança e efeitos do uso de CTA na recuperação dos tecidos uretrais lesados.

Dentre as CTA, as mais utilizadas e estudadas em Uroginecologia são as células-tronco derivadas de músculo (CTDM), as células-tronco derivadas de adipócitos (CTDA) e as células-tronco derivadas de medula óssea (CTMO).

1.3 O uso das células-tronco derivadas de músculo na regeneração uretral

As células-tronco derivadas de músculo (CTDM) diferenciam-se principalmente em células mesodérmicas (músculo estriado, gordura, cartilagem e osso). São obtidas por meio da purificação de amostras de músculo estriado e cultivadas in vitro[14]. As células cultivadas podem então ser injetadas diretamente no local a ser tratado (tecido lesado) ou na circulação sanguínea, dependendo do tipo de regeneração que se pretende.

Chermansky et al demonstraram que após injeção periuretral, as CTDM se integraram às camadas de músculo estriado esfincteriano de uretras de ratas após 4 semanas da lesão uretral por cauterização. Além disso, houve regeneração da inervação no grupo injetado com CTDM quando comparado com o grupo placebo que recebeu solução salina, o que sugere a multipotência das CTDM. Neste mesmo estudo, a pressão de perda urinária (pressão intravesical na qual ocorre perda urinária durante a manobra de Valsalva) foi significativamente maior no grupo com CTDM em comparação com o grupo controle, sugerindo a melhor função uretral no grupo tratado. Não houve

diferença significativa da pressão de perda do grupo tratado após 4 e 6 semanas da lesão quando comparado ao grupo não-lesado, sugerindo que a função uretral que garante a continência urinária do grupo que recebeu CTDM é semelhante ao grupo de ratas normais, portanto, re-estabelecida[15].

Estudo recente demonstrou os efeitos benéficos da terapia celular com CTDM em macacas que apresentavam IUE causada por meio da transecção dos nervos pudendos das mesmas. Os autores realizaram análises histológicas e estudo urodinâmico funcional para avaliação da terapia após um ano. Observaram que a terapia aumentou a pressão máxima de fechamento uretral das macacas, maior proporção da área muscular estriada e menor área ocupada pelas fibras colágenas nas uretras de animais tratados, quando comparados aos controles não tratados. Após 3 meses da injeção, foram detectados marcadores musculares (desmina, conexina-43 e α-actina de músculo liso) e fator de Von Willebrand marcados com a proteína GFP fluorescente das células-tronco. Deste modo, comprovou-se a diferenciação das CTDM em tecido muscular e vasos sanguíneos. O estudo demonstra os resultados benéficos da terapia com CTDM para IUE em longo prazo[16].

Carr e colaboradores publicaram recentemente estudo clínico envolvendo 38 mulheres com IUE refratárias a um ano de tratamento clínico convencional. Na fase 1, as participantes foram randomizadas em cinco grupos de 4, de acordo com a quantidade de células que receberam: 1, 2, 4, 8 e 16 milhões de CTDM. Na fase 2, 3 grupos de 3 participantes receberam 32, 64 e 128 milhões de células, e, na fase 3, outros 3 grupos de 3 participantes receberam 16, 32 e 64 milhões de células injetadas na região peri-uretral por cistoscopia. As pacientes poderiam optar por uma segunda injeção após 3 meses. A avaliação deu-se pela análise periódica do diário miccional, do teste do absorvente e do questionário de qualidade de vida durante 12 meses de seguimento. Os resultados da terapia foram melhores proporcionalmente ao número de células utilizadas. Quando comparado com os grupos de baixas doses, maior número de participantes dos grupos que receberam altas doses de células apresentaram redução de pelo menos 50% do peso do absorvente durante o teste do absorvente (88,9%, 8 de 9 vs 61,5%, 8 de 13), apresentaram redução de pelo menos 50% das perdas urinárias aos esforços no diário miccional (77,8%, 7 de 9 vs 53,3%, 8 de 15); e tiveram até um episódio de perda durante 3 dias (88,9%, 8 dos 9 vs 33,3%, 5 dos 15) no seguimento final de um ano. Os

efeitos colaterais relatados foram poucos: dor na região da biópsia muscular e do sítio de injeção, retenções urinárias leves e temporárias, e infecções urinárias[17].

Resultados concordantes com relação à segurança e eficácia da terapia celular com injeções locais peri-uretrais de CTDM para o tratamento da IUE feminina foram observados por Peters e colaboradores (2014). Por meio de semelhante metodologia, os autores avaliaram 80 mulheres que receberam injeções no esfíncter uretral externo de 10 (n=16), 50 (n=16), 100 (n=24), or 200 (n=24) milhões de CTDM autólogas obtidas de biópsias do músculo quadríceps femoral. Todos os grupos tiveram melhora estatisticamente significativa na pontuação dos questionários específicos para avaliar incontinência urinária (UDI-6 e IIQ-7) após 6 e 12 meses de seguimento, quando comparados com o estado pré-tratamento. De modo geral, a melhora nos par6ametros clínicos fora dependente da dose utilizada. 85% das pacientes que receberam 100 milhões de células e 77% que receberam 200 milhões de células-tronco apresentaram redução de pelo menos 50% das perdas por esforço pela análise do diário miccional, instrumento que diferencia os tipos de incontinência urinária. Ambas as doses utilizadas melhoraram os valores do teste de absorvente, sendo significante no grupo de 200 milhões de células. No entanto, os autores ressaltam que cerca de 50% das pacientes avaliadas apresentavam incontinência urinária mista. Sendo assim, os valores do teste do absorvente não diferenciariam as perdas por esforço ou por urgência, colocando em dúvida as conclusões baseadas somente no teste do absorvente. Os autores não observaram efeitos adversos sérios ou persistentes com a terapêutica em qualquer dose administrada[18].

Estudo randomizado único comparou a injeção uretral de CTDM autóloga com 3 diferentes técnicas cirúrgicas para correção de IUE (“Burch, sling TVT e sling TOT”). O estudo envolveu 8 mulheres que receberam a terapia celular, 11 no grupo Burch, 26 casos de sling TVT, e 41 casos de sling TOT. Os autores reportaram discreta, mas não significante, melhora nos parâmetros funcionais do estudo urodinâmico após a terapia com CTDM. Os resultados foram inferiores aos obtidos com os procedimentos cirúrgicos, com a necessidade de aumento da amostra para concluir sobre os efeitos da terapia celular[19].

1.4 O uso das células-tronco derivadas de adipócitos na regeneração uretral

As CTDA são obtidas da gordura branca (a mais predominante em adultos), sendo que o tecido adiposo é composto por 40-60% de adipócitos maduros, e por uma fração estromal, esta composta por fibroblastos, macrófagos, mastócitos, células endoteliais, células hematopoiéticas e pré-adipócitos. Os pré-adipócitos são as precursoras denominadas CTDA[20]. Foi demonstrado que estas podem se diferenciar *in vitro* em células adipogênicas, miogênicas e osteogênicas[21]. Podem ter um papel no tratamento da incontinência urinária. Jack et al[22] demonstraram formação de músculo liso a partir das CTDA em detrusor, e com capacidade de contração e relaxamento; enquanto Zeng et al[23] conseguiram bons resultados em modelos animais com ratas, melhorando a LPP e a função uretral após a injeção das CTDA em uretras lesadas.

Na prática clínica, há um relato de dois casos de pacientes que receberam injeção de CTDA após prostatectomia radical[24]. Estes tinham incontinência urinária de esforço (IUE) moderada após a cirurgia há pelo menos 2 anos, não faziam tratamento para a IUE e não apresentavam recidiva da doença. Foram retirados 250 ml de tecido adiposo da parede abdominal anterior, e as CTDA foram posteriormente isoladas. A seguir, as células foram injetadas no tecido parauretral na região do esfíncter externo da uretra por via cistoscópica. Foram aplicados 1 ml de solução com CTDA puras no rabdoesfíncter (profundidade de 5mm), e 20 ml de solução de tecido adiposo íntegro e CTDA na submucosa, como agente selante. Os resultados foram animadores: melhora progressiva da IUE (medida por "pad test" de 24 horas e questionário), aumento do comprimento funcional da uretra e da pressão média de fechamento uretral. A ultrassonografia mostrou que o material injetado na região parauretral não desapareceu após 12 semanas, além de haver fluxo sanguíneo ao Doppler na área em que as CTDAs foram aplicadas. Não houve relatos de efeitos colaterais.

1.5 O uso das células-tronco derivadas de medula óssea na regeneração uretral

A medula óssea é fonte muito importante de células-tronco. A linhagem derivada do mesênquima (célula-tronco mesenquimal - CTM) é a mais importante neste campo, pois pode desenvolver osso, cartilagem, gordura, tecido conjuntivo[25-27]. A maioria dos trabalhos com estas células estudou a regeneração vesical em modelos animais, com a colocação de matrizes acelulares semeadas com as CTM[29-32]. As células foram cultivadas *in vitro* e semeadas nas matrizes acelulares (submucosa intestinal), que

foram implantadas nas bexigas dos animais, e houve desenvolvimento de músculo liso funcional a partir das CTM. Além destes, há um estudo de Kinebuchi et al[33], que analisa a regeneração do esfíncter uretral de ratas. Após 7 dias de lesão realizada cirurgicamente, as ratas foram operadas novamente e receberam injeção de 200 a 500.000 CTMs suspensas em meio de cultura no tecido periuretral, e comparadas com animais que receberam injeção somente do meio de cultura das células e com animais sem lesão uretral. Os valores de LPP de cada grupo foram avaliados antes da lesão uretral, e 1, 4, 6, 8 e 12 semanas após a injeção (células ou meio de cultura puro). As ratas sem lesão não apresentaram diferença nas medidas de LPP. As ratas lesadas e tratadas com injeção somente do meio de cultura tiveram o LPP reduzido significativamente após 1 semana, e não se recuperaram até a 12ª semana. Já as ratas tratadas com CTM tiveram recuperação gradual do LPP, mas não significativamente maior que no grupo com injeção do meio de cultura puro. Histologicamente, houve proliferação de musculatura esquelética no grupo com CTM, mas não de músculo liso. Adicionalmente, observou-se aumento de inervação no grupo com CTM.

1.4 Experiência da UNIFESP-EPM

O Setor de Uroginecologia e Cirurgia Vaginal do Departamento de Ginecologia da UNIFESP-EPM tem sua linha de pesquisa desenvolvida com CTA. Este projeto de pesquisa teve apoio inicial do Conselho Nacional de Desenvolvimento Científico e Tecnológico: Edital CT-Saúde/MS/SCTIE/Decit/MCT/CNPq Nº 17/2008.

Analisamos o efeito da administração das células-tronco derivadas de músculo, de adipócitos e de medula óssea em modelo animal de trauma uretral por distensão vaginal (DV), que mimetiza as lesões uretrais decorrentes do parto vaginal. Estudamos e comparamos as uretras de três grupos de ratas: controle, grupo de DV e grupo de DV tratado com células-tronco. A técnica de DV fora desenvolvida e padronizada em nosso laboratório, na qual uma sonda de Foley fora inserida no interior da vagina de ratas com o balão mantido insuflado com 3 ml de água por período de 12 horas intermitentes. As lesões uretrais foram confirmadas em análises histológicas.

As células-tronco foram obtidas de ratas mutadas SD-Tg(GFP)2BalRrrrc, que expressam o marcador “green fluorescent protein” ou GFP em suas células, facilmente detectadas devido à cor verde que refletem sob a luz fluorescente (Figura 1).

As células foram cultivadas e expandidas em meio apropriado. Conferimos a autenticidade das CT por meio da combinação de ensaios de diferenciação celular, imunofenotipagem e determinação das fases do ciclo celular. As CTMO, por exemplo, diferenciaram-se in vitro em adipócitos, condroblastos e osteoblastos, caracterizando seu potencial de maturação em células dos três folhetos germinativos (Figura 2).

Finalizada a etapa do cultivo das células, realizamos o tratamento das ratas após 72 horas da lesão por DV, injetando as CT na veia caudal dos animais. A seguir, a identificação das CT-GFP e a morfologia dos tecidos uretrais foram avaliadas nos dias 7, 14, 21 e 28 após a terapia, utilizando-se análise histoquímica e microscopia eletrônica.

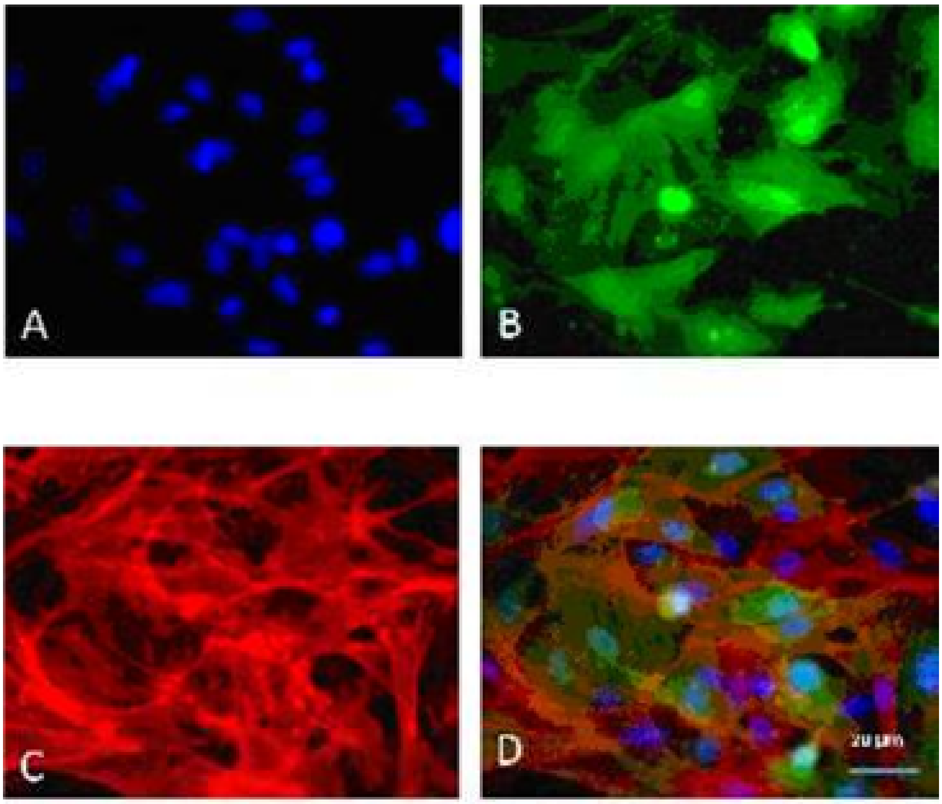

Figura 1- Microscopia de Fluorescência: Imuno-localização das CTMO-GFP. A: Núcleo corado em DAPI (azul); B: CTMO-GFP (verde); C: Citoesqueleto corado em rodamina (vermelho); D: Co-localização.

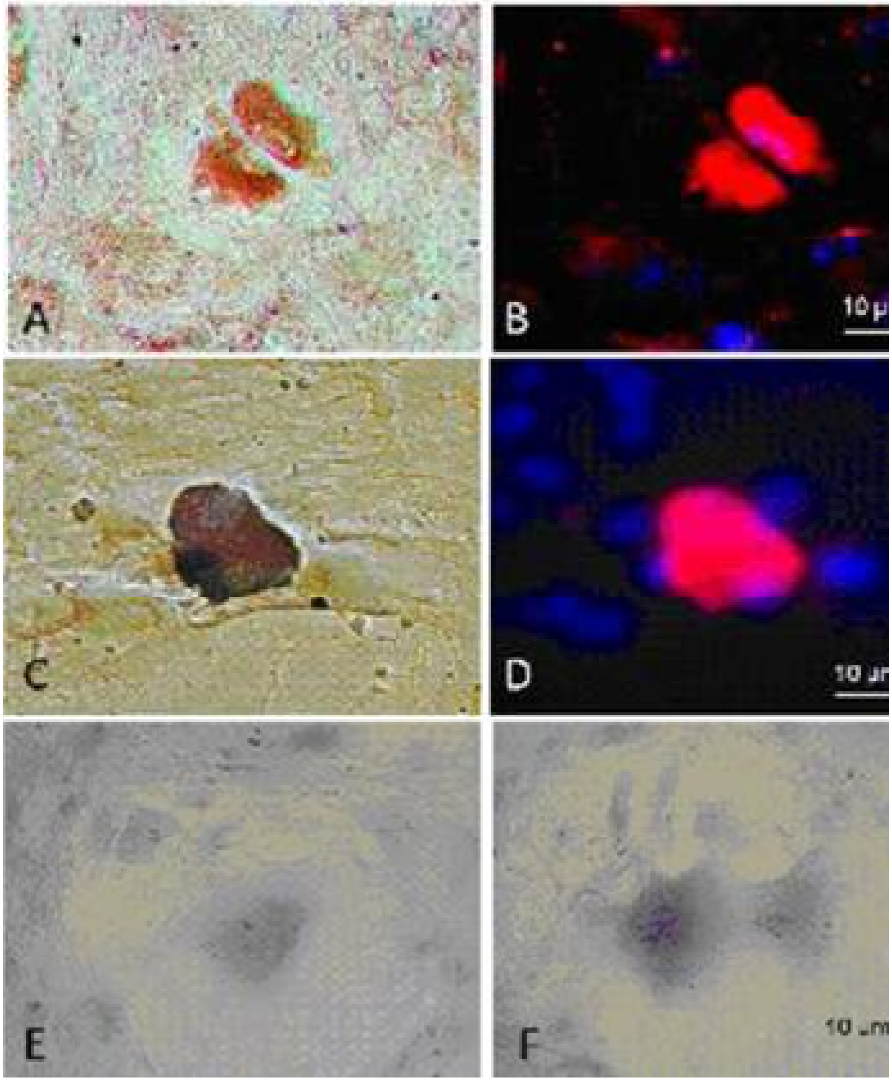

Figura 2 - Fotomicrografia demostrando a plasticidade das CTMO. Diferenciação das CTMO: A,B - adipócitos (oil red O); C,D - osteoblastos (vermelho de alizarina); E,F - condroblastos (azul de toluidina). Bar: 10µm

Observamos que as ratas submetidas ao trauma por DV apresentaram desorganização nas camadas histológicas da uretra, caracterizada por estreitamento e rotura das fibras musculares lisas e estriadas.

Ao final de 28 dias pós-trauma, observamos a presença de tecido conjuntivo em substituição parcial às fibras musculares lisas, sugerindo processo cicatricial (Figura 3).

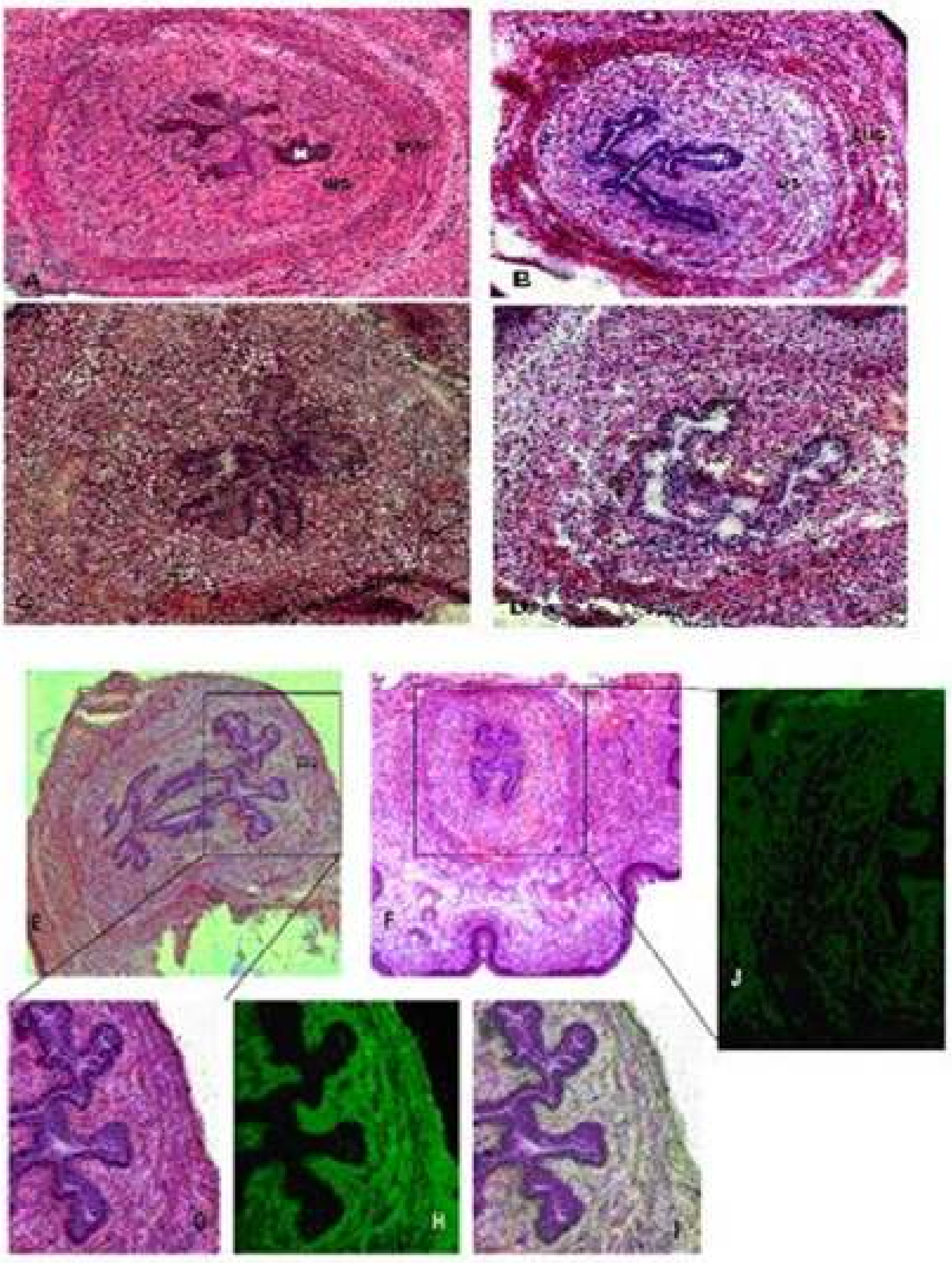

Figura 3 – A-D: Análise histológica (HE) demonstrando os efeitos da DV na uretra das ratas. A,B: controle; C,D: grupo DV 7 e 28 dias pós-trauma, respectivamente. E-J: Análise histológica (HE) e detecção das CTMO-GFP: E,F: Grupos DV e terapia celular 7 e 28 dias pós-trauma, respectivamente. G,H,I: Figura E no detalhe com HE, GFP e co-localização, respectivamente. J: Figura F no detalhe mostrando as células GFP 28 dias pós-trauma. M: mucosa; IUS: esfíncter uretral interno; EUS: esfíncter uretral externo. Magnificação 40x. Bar: 50µm

Notamos a presença precoce (em 7 dias após a injeção) das células GFP em todas as camadas uretrais, demonstrando a migração, integração e sobrevivência das mesmas no tecido uretral. A terapia com CT melhorou a organização estrutural das uretras e a remodelação tecidual em comparação com as ratas traumatizadas não tratadas. Após 4 semanas do tratamento, observamos iguais proporções de músculo liso e tecido conjuntivo na uretra, bem como a ampla recuperação da musculatura estriada, ambos semelhantes ao padrão estrutural das ratas controle (Figura 3).

Nossos resultados com o uso de CTMO, CTDM e CTDA, e efeitos na uretra foram muito semelhantes.

Análises imunohistoquímicas utilizando-se de marcador desmina (marcador de musculatura lisa e estriada), demonstraram a melhora da organização estrutural e na espessura das camadas musculares uretrais das ratas que foram tratadas com CTDM, se comparada com as ratas não tratadas após o trauma (Figura 4). Estes achados são sugestivos do efeito benéfico da terapia com CTDM na regeneração uretral.

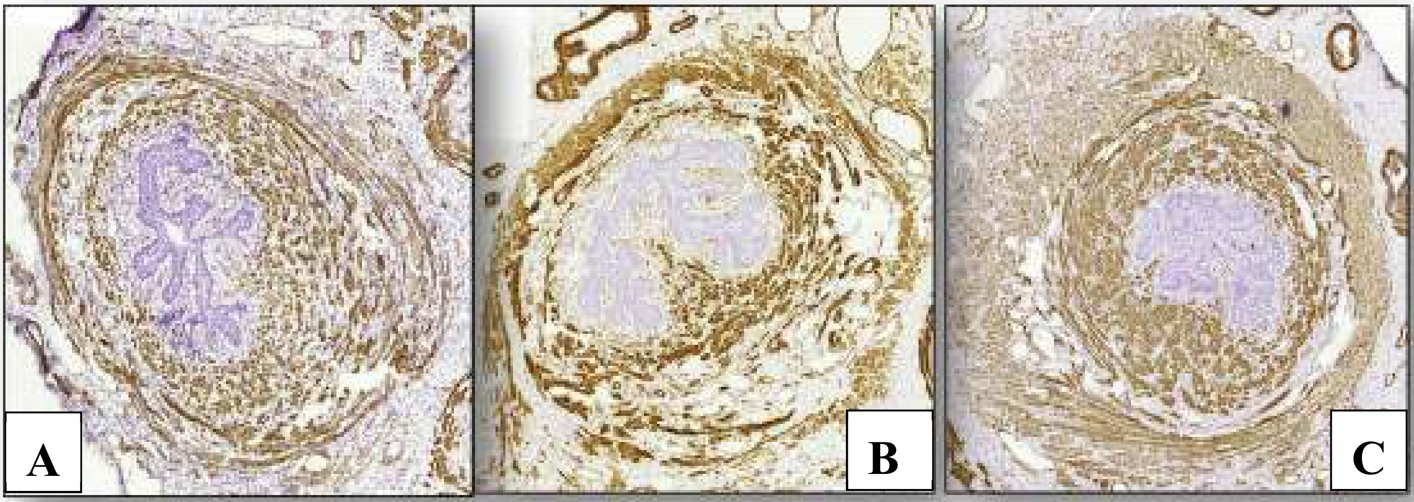

Figura 4: Imagens dos cortes uretrais de reação imunohistoquímica com marcador desmina (corado em marrom); A. Grupo Controle; B. Grupo 28 dias pós-trauma sem terapia; C. Grupo 28 dias após terapia com CTDM. Notar o aumento da área de musculatura lisa e estriada no grupo tratado.

A fim de entender os efeitos da terapia celular com CTDM em nível molecular, e associarmos com os achados histológicos, realizamos estudos de expressão gênica e protéica das uretras de ratas. Comparamos os grupos controle sem trauma por DV, o grupo DV sem terapia celular após 28 dias do trauma, e o grupo que sofreu DV e fora

tratado com CTDM. Foram estudados marcadores de musculaturas lisa e estriada, marcadores de proliferação celular e fatores de crescimento.

Com relação às expressões gênicas, observamos aumento do gene de proliferação celular Ki67, dos genes de colágenos 1 e 3 no grupo que recebeu CTDM; enquanto que os genes de cadeia pesada dos músculos liso e estriado, bem como o fator de crescimento neural NGF encontravam-se aumentados nos grupos de trauma, e praticamente retornaram a seus níveis basais após o tratamento. O fator de crescimento vascular VEGF não mostrou-se alterado nos diferentes momentos (Figura 5).

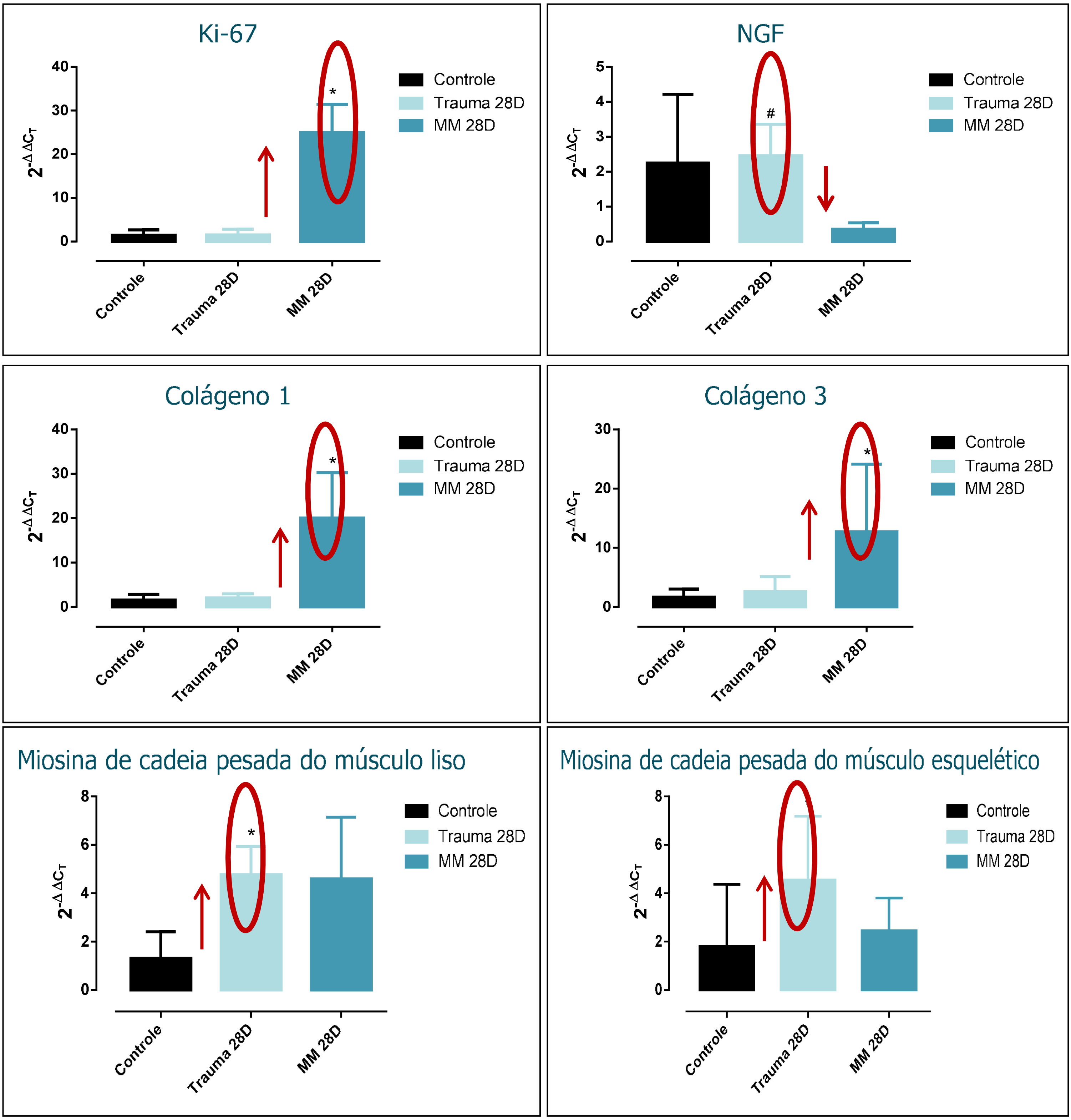

Figura 5: Representação esquemática das expressões gênicas entre os grupos. * p <0.05.

Focamos nossa análise das proteínas nos componentes musculares: miosinas de cadeia pesada de músculos liso (MHY11) e estriada (MHY1), e observamos aumento significativo das expressões protéicas nos grupos de ratas que receberam terapia com CTDM, demonstrando os efeitos regenerativos da terapêutica uretral (Figura6).

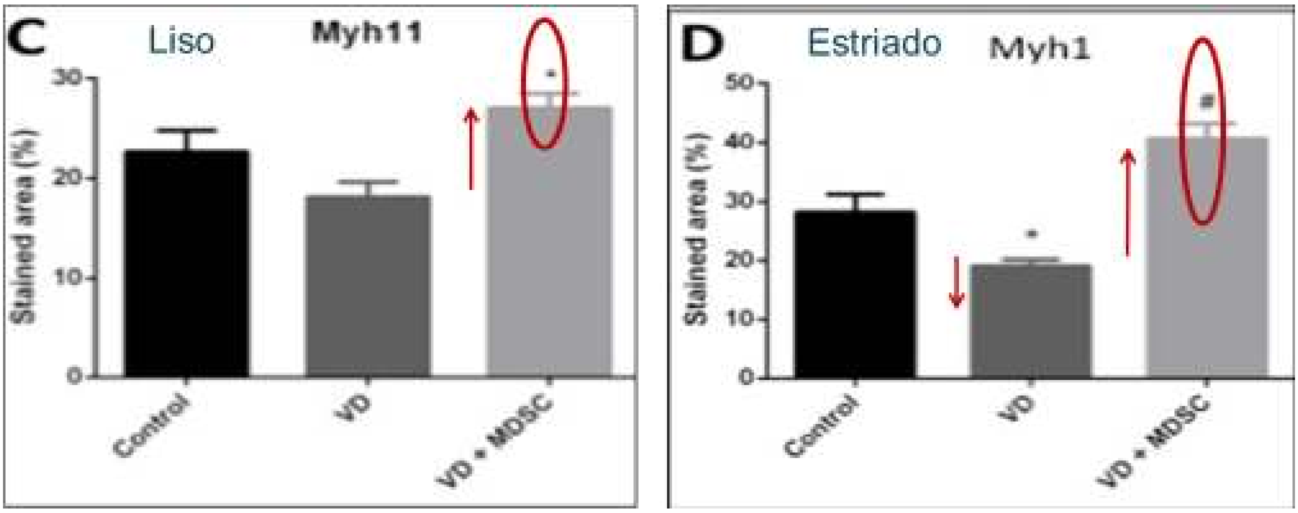

Figura 6: Representação esquemática dos níveis protéicos entre os grupos. * p <0.05.

As imagens imunohistoquímicas confirmam os efeitos da terapia nas camadas musculares da uretra (Figuras 7 e 8).

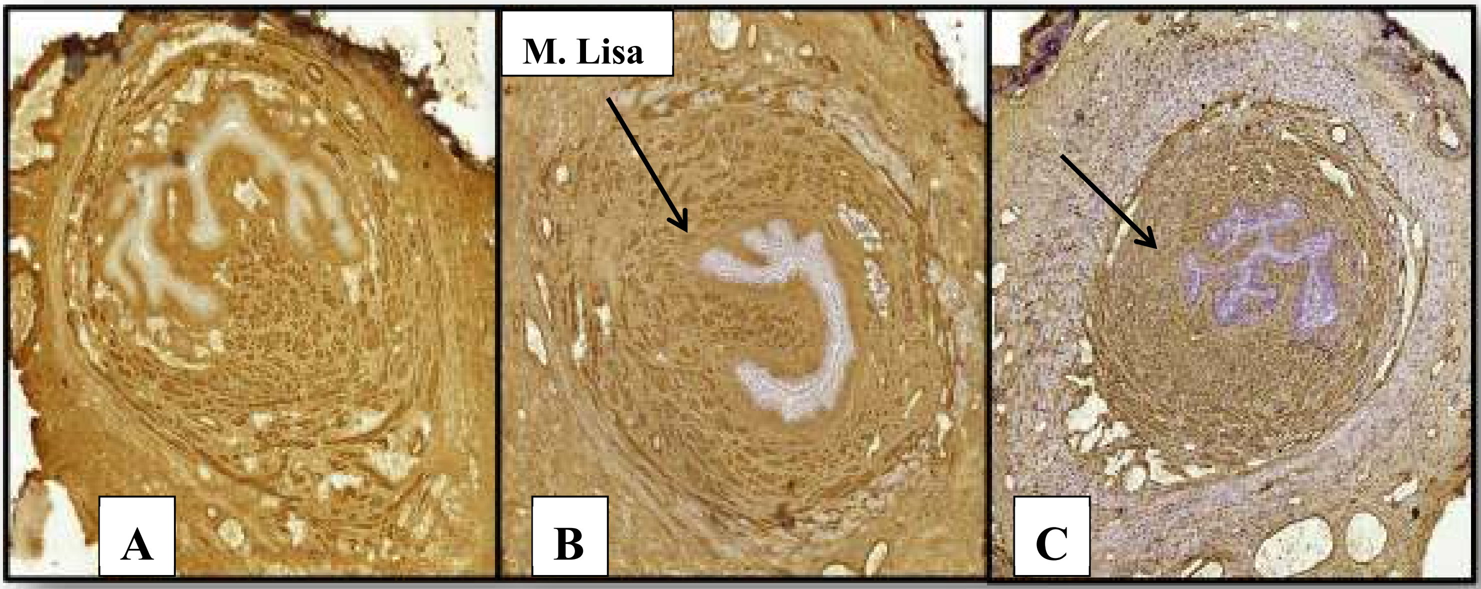

Figura 7: Imagens dos cortes uretrais de reação imunohistoquímica com marcador MHY1 (corado em marrom); A. Grupo Controle; B. Grupo 28 dias pós-trauma sem terapia; C. Grupo 28 dias após terapia com CTDM. Notar o aumento da área de musculatura lisa no grupo tratado.

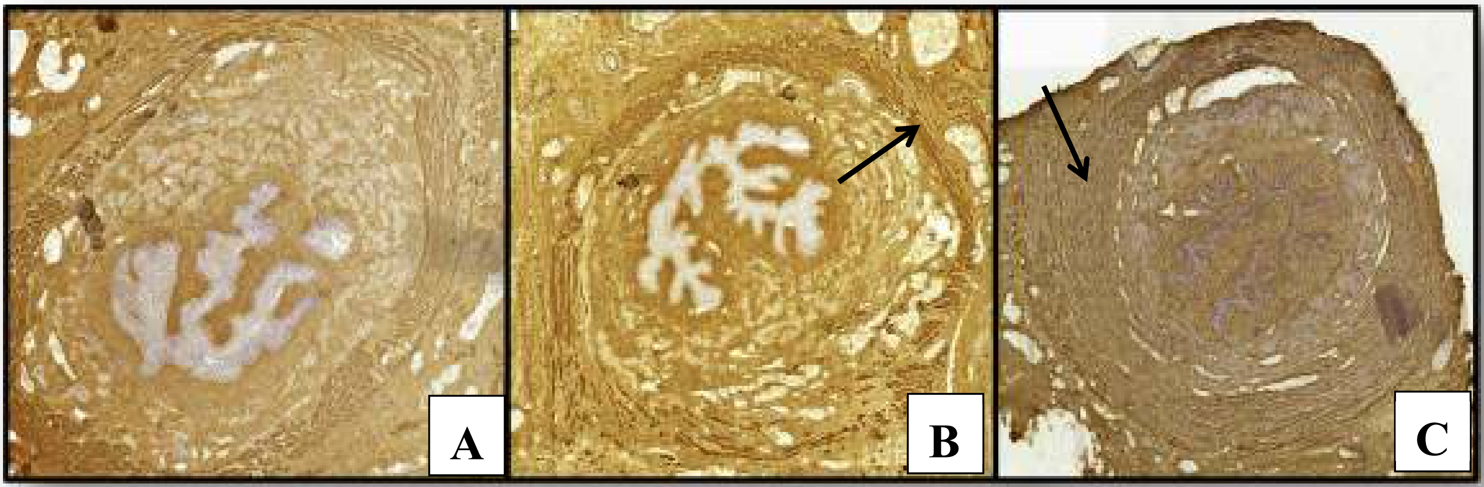

Figura 8: Imagens dos cortes uretrais de reação imunohistoquímica com marcador MHY11 (corado em marrom); A. Grupo Controle; B. Grupo 28 dias pós-trauma sem terapia; C. Grupo 28 dias após terapia com CTDM. Notar o aumento da área de musculatura estriada no grupo tratado.

Além destes, este estudo demonstra a segurança das terapias com CTA. Não observamos casos de rejeição, formação de tumores ou outras complicações em nossos ensaios.

2. Resultados esperados

Diante dos achados experimentais que sugerem significativa recuperação dos componentes e estrutura das uretras, a terapia celular com CTA poderia ser viável e útil no tratamento das mulheres com IUE, uma vez que ajudaria o processo de regeneração das uretras lesadas, como ocorre no trauma de parto vaginal.

No entanto, os efeitos da terapia celular na prática clínica ainda não foram suficientemente demonstrados, havendo necessidade de adicionais estudos clínicos para

comprovar sua viabilidade e potencial benefício para as mulheres incontinentes. Com isto, interessou-nos a continuidade neste campo de pesquisa.

Esperamos demonstrar que a terapia com CTA derivadas de músculo esquelético (CTDM), de medula óssea (CTMO) e de adipócitos (CTDA), são viáveis no tratamento da IUE e melhoram a qualidade de vida e as perdas urinárias nas mulheres acometidas.

3. Desafios científicos e tecnológicos

3.1 Objetivo geral:

O objetivo deste trabalho é avaliar a viabilidade e efeitos da terapia com injeção uretral de CTMO, CTDM e CTDA em mulheres com incontinência urinária de esforço.

3.2 Objetivos específicos:

- Isolamento e cultivo *in vitro* das CTDM, CTMO e CTDA autólogas provenientes de pacientes uroginecológicas com IUE;

- Análise da viabilidade da terapia celular intra-uretral em mulheres com IUE quanto à metodologia e potenciais efeitos adversos;

- Avaliação de mulheres após a terapia celular utilizando parâmetros objetivos que avaliam as perdas urinárias: teste de esforço, teste do absorvente e a avaliação da pressão de perda uretral por meio do estudo urodinâmico;

- Avaliação de mulheres após terapia celular utilizando parâmetros subjetivos por meio da aplicação de questionário de qualidade de vida específico para IU (I-Qol).

4. Metodologia

4.1 Tipo de estudo e população: Estudo clínico, randomizado prospectivo envolvendo 45 pacientes com IUE que receberão injeção peri-uretral de 5ml de solução contendo 100 milhões de CTA autólogas (derivadas de músculo, medula óssea ou tecido adiposo).

4.2 Local do estudo: Este é um estudo conjunto entre o Setor de Uroginecologia, Disciplina de Ginecologia Geral do Departamento de Ginecologia da Universidade Federal de São Paulo (UNIFESP), o Setor de Hematologia e Hemoterapia do Hospital

Israelita Albert Einstein (HIAE), São Paulo, SP, e a empresa privada Stemcorp, São Paulo, SP.

4.3 Participantes

Critérios de inclusão

1. Pacientes com IUE moderada, primária e refratária a tratamentos clínicos prévios (terapia comportamental e fisioterapia), confirmada por exame físico (teste de esforço), pelo questionário I-Qol, pela cistometria (estudo urodinâmico) e pelo teste do absorvente (entre 10 e 50 gr).

Critérios de exclusão

1. Pacientes com prolapso de parede vaginal anterior ou uterino maior que estádio 2 (classificação POP-Q);
2. Pacientes submetidas a procedimentos cirúrgicos para correção de IUE prévios;
3. Pacientes com malformações genitais que impossibilitem a injeção no sítio de terço médio da uretra;
4. Pacientes submetidas à radioterapia pélvica prévia;
5. Pacientes que durante a avaliação clínica e/ou urodinâmica apresentem outros diagnósticos que não somente a IUE (incontinência mista, urge-incontinência, hiperatividade detrusora, incontinência paradoxal, bexiga neurogênica, obstrução urinária);
6. Pacientes não concordantes com a participação no estudo em qualquer etapa do mesmo ou não forem capazes de ler, escrever e compreender o estudo;
7. Pacientes < 18 anos de idade.

4.4 Local da realização dos procedimentos: As pacientes serão recrutadas e avaliadas (pré e pós-tratamento) no Ambulatório de Uroginecologia da UNIFESP. Os procedimentos de biópsias de medula óssea, isolamento e cultivo celulares serão realizados no laboratório de terapia celular em humanos do Setor de Hematologia e Hemoterapia do Hospital Israelita Albert Einstein, São Paulo. Os procedimentos de biópsia de tecido adiposo e de músculo bíceps serão realizados no centro cirúrgico do

Hospital São Paulo, e os isolamento e cultivo celulares serão realizados no laboratório de terapia celular da Stemcorp, São Paulo.

4.5 Testes diagnósticos pré e pós-terapia

a) Exame clínico – teste de esforço: Para o diagnóstico da IU será realizado o teste de esforço, no qual a perda de urina é evidenciada no momento que a paciente realiza manobras de esforço com a bexiga confortavelmente cheia.

b) Teste do absorvente: O teste do absorvente será empregado para quantificar a perda de urina. Com a bexiga vazia, 250 ml de água destilada será infundida na bexiga. Posteriormente, será colocado um absorvente na região perineal com o peso previamente aferido, e as pacientes realizarão as seguintes manobras de esforço: tossir, pular, agachar e contrair os músculos abdominais (Valsalva), 10 vezes cada. Subirão ainda cinco degraus de escada por 10 vezes consecutivas, lavarão as mãos por um minuto e caminharão por 20 minutos. O tempo para a realização do exame não ultrapassa uma hora. A seguir, será aferido o peso do absorvente; quando a diferença for maior que 2 gramas considera-se o teste positivo[34].

c) Cistometria: o aparelho utilizado para o exame urodinâmico será Urosystem PL-2400 Polimed de quatro canais. A cistometria será efetuada em posição semi-sentada, por cateterização uretral com duas vias, uma para o enchimento da bexiga e a outra para a medida da pressão intra-vesical. Utilizar-se-á água destilada à temperatura ambiente, com velocidade de 40ml/min. A pressão abdominal será monitorada por meio de balão retal[1]. Na fase de enchimento, com 200 ml infundidos, as pacientes farão manobra de Valsalva de modo a determinarmos o valor da pressão intra-vesical durante a manobra, em que ocorre a perda de urina pela uretra. Será considerado positivo o teste sempre que houver perda urinária, e a pressão de perda será mensurada.

d) Questionários de qualidade de vida: Usaremos o "Incontinence Quality of Life Questionnaire (I-QoL)" na análise da qualidade de vida. O "I-QoL" é composto por vinte e duas questões organizadas em três domínios, que analisam a limitação do comportamento humano, o impacto psicossocial e o embaraço e o constrangimento social. A todas as respostas são atribuídos valores que variam

de 1 a 5, sendo, portanto, a qualidade de vida mensurada de 0 a 100. Quanto menor o número obtido, pior a qualidade de vida[35].

e) Avaliação de segurança da terapia celular: As participantes do estudo terão acesso irrestrito ao centro de pesquisa e aos investigadores durante e após a finalização do estudo, de forma a mantermos a vigilância quanto à sua segurança.

Nas consultas de seguimento pós-operatório serão avaliadas a presença de efeitos adversos tais como: sintomas irritativos (aumento da frequência miccional, disúria, dor supra-púbica, urgência e urgeincontinência); sintomas sugestivos de infecção urinária; sintomas obstrutivos (sintomas irritativos, sensação de esvaziamento vesical incompleto, diminuição de jato urinário, necessidade de "fazer força" para urinar); infecção ou coleções no sítio da punção; prurido ou infecção vulvovaginal; hematúria. Na suspeita de ITU, urinálise com urocultura serão realizados para confirmação diagnóstica. Na suspeita de obstrução urinária parcial ou total, será realizada fluxometria livre e medida do resíduo pós-miccional por método ultrassonográfico para complementação diagnóstica. Consideraremos obstrução parcial os quadros de resíduo pós-miccional > 150 ml; nestes casos a conduta será expectante, com medidas comportamentais no auxílio do esvaziamento vesical. Diante de quadro de obstrução total após injeção peri-uretral, a paciente será mantida com cateterismo de demora, com visitas semanais de acompanhamento do seu padrão miccional. O cateterismo de demora será finalizado quando a paciente apresentar resíduo < 150 ml.

Não antecipamos efeitos adversos sérios decorrentes da terapia com células-tronco adultas, que comprovadamente não oferece riscos de formação de tumores. Além disto, estudos prévios de terapia celular em IUE demonstraram a segurança do tratamento[16-18]. Conforme descrito nas próximas seções, utilizaremos de métodos apropriados de identificação e caracterização das células-tronco.

f) Conduta nas falhas das etapas do estudo: Nos eventuais casos onde haja impossibilidade de realização dos procedimentos tanto laboratoriais, quanto os procedimentos de obtenção ou injeção das células-tronco, por dificuldades técnicas ou desconforto, as pacientes terão sua participação no estudo interrompida. Na eventualidade de efeitos adversos, a paciente será prontamente assistida pela equipe, e terá toda a estrutura ambulatorial e hospitalar (Hospital São Paulo) disponível caso necessário.

4.6 Método de Preparação das CTMO

I – Isolamento de células-tronco derivadas de medula óssea de cultura primária de biópsia medular

Coleta: Com material e assepsia apropriada, sob anestesia local com xilocaína a 2%, será realizada punção da medula óssea das cristas ilíacas anterior e posterior das pacientes, a depender da avaliação individual, sendo extraída cerca de 50 ml da solução medular.

Processamento e cultivo celular: Todas as etapas do processamento serão realizadas de maneira estéril em capela de fluxo laminar específica para este fim. O material medular será cultivado em meio Eagle modificado por Dulbecco. Com uso de Ficoll-Paque, a solução foi centrifugada a 500rpm por 30 min a 22°C. A seguir, foi coletado o anel de células na interface Ficoll-solução, suspendido em solução tampão fosfato para ser lavado por 3 vezes e retirado qualquer resíduo de Ficoll. As células foram suspensas em meio DMEM, soro fetal bovino, penicilina e estreptomicina e colocadas em estufa por 24 horas para a aderência ao plástico.

A solução de meio DMEM, com as células ressuspendidas, foi distribuída em garrafas de cultura de 75 cm2 e DMEM e mantida em incubadora úmida a 37°C e 5% de CO2. No ambiente estéril, após 24 horas do isolamento, com células-tronco aderidas na garrafa de cultura, o meio foi removido por sucção utilizando uma pipeta estéril tipo Pasteur ponta capilar fina. Depois a garrafa foi lavada três vezes com PBS contendo penicilina e estreptomicina a 37°C e foi adicionado DMEM. Para a manutenção das células, foi renovado o meio de cultura a cada 48 horas.

Quando as células aderidas na garrafa atingiram aproximadamente 80% de confluência, foi realizada a passagem através da tripsinização. Esse procedimento tem por objetivo desprender as células da garrafa e transferi-las para outro recipiente e/ou utilizá-las em procedimentos posteriores (células utilizadas entre segunda passagem e terceira passagem). Para a tripsinização, a garrafa teve seu meio de cultura retirado por sucção e foi lavada, utilizando uma solução de PBS suplementado com 0,5M de EDTA. Em seguida a solução foi retirada por sucção e acrescentada a garrafa uma solução de 0,10% de tripsina e 0,02% de EDTA por 3 min em igual proporção de volume da tripsina. A tripsina foi neutralizada utilizando meio DMEM. A suspensão de células foi

centrifugada a 300rpm por 6 min, o sobrenadante foi retirado por sucção e as células foram ressuspensas em meio DMEM e a células foram mantidas em incubadora úmida a 37°C e 5% de CO2 até atingirem novamente 80% de confluência.

II – Isolamento de mioblastos e fibroblastos de cultura primária de biópsia de músculo

Coleta: Sob anestesia local, serão coletadas amostras do M. Quadríceps Femoral (aproximadamente 0,3 cm^3) por meio de punção-biópsia com agulha percutânea, que será levada para o laboratório em meio de transporte contendo 15 mL de PBS 1X com 10 ug/mL de anfotericina B, 4% de Penicilina/Estreptomicina (10.000 UI, 10 mg/mL). O transporte será realizado em recipiente térmico com gelo e à temperatura de 4±2ºC. O processamento deve ocorrer em até 24 horas após a coleta.

Processamento: Todas as etapas do processamento serão realizadas de maneira estéril em capela de fluxo laminar específica para este fim. Em resumo, o fragmento sofrerá digestão enzimática com colagenases tipos II e IV, para a obtenção dos dois tipos celulares. O material será centrifugado por 400g por 5 minutos e o botão de células formado será re-suspendido nos meios de cultivo apropriados.

Cultivo Celular: Caso seja necessária a expansão ou aumento no número de células, esta será cultivada em garrafas tratadas com Cellstart$^-$ CTS^{TM} (Invitrogen) em meio de cultivo DMEM: F12 20% SFB para fibroblastos, e meio de cultura quimicamente definido, MCDB 153 para miócitos, ambos acrescidos de 1% de glutamina, 1% anfotericina B (solução de 250 ug/mL) e 1% de penicilina/estreptomicina (10.000 UI, 10 mg/mL). As células deverão ser congeladas ou utilizadas entre a 3º e a 5º passagem.

III – Isolamento de adipócitos de cultura primária de biópsia de tecido adiposo

Coleta: Amostra de lipoaspirado será coletada após assepsia e antissepsia seguida da infiltração de 50 cc de solução de lidocaína 0,25% com adrenalina 1:500.000 na região de parede abdominal. O material será aspirado por meio de seringas com cânulas de 3 mm de diâmetro.. As amostras serão imediatamente transportados em frasco de cultura estéril de 100 ml contendo 50 ml de solução salina balanceada Hank's (HBSS), com 100 U/ml de penicilina e 100 µg/ml de estreptomicina.

Processamento: No ambiente estéril, cabine de fluxo laminar, os fragmentos serão lavados em seis béqueres com 20 ml de solução HBSS com auxílio de pinças estéreis e

os fragmentos serão transferidos para uma placa de Petri de 100 mm^2 de diâmetro e dissecado o tecido adiposo, cortado em pequenos fragmentos de aproximadamente 0,5 mm^3 com auxílio de uma tesoura de Íris. Os fragmentos resultantes serão transferidos para um frasco de vidro de 100 ml estéril e acrescentado colagenase tipo II (Sigma Chemical Company, St Louis, MO, EUA), 1 mg/ml em HBSS contendo na proporção 5 volumes de colagenase para 1 volume do tecido obtido, submetidos à agitação por 10 minutos a 37°C. A solução obtida será filtrada em malha de nylon de 250 µm. O filtrado obtido será colocado em tubos cônicos estéreis de 50 ml e centrifugado por 10 minutos a 300 g em temperatura ambiente.

Cultivo Celular: O precipitado será transferido para um tubo cônico de 50 ml e lavado com uma solução de 20 ml de meio de cultura Eagle modificado por Dulbecco (DMEM)/Mistura de Nutrientes F-12 (HAM) obtido da Sigma Chemical Company, St Louis, MO, EUA, suplementado com 10% de soro fetal bovino (SFB), e 0,1% de albumina sérica bovina (BSA), 100 U/ml de penicilina e estreptomicina.

IV – Caracterização, controle de qualidade e biosegurança das populações celulares

A fim de garantir a qualidade das amostras e das células cultivadas, serão realizados: citometria de fluxo para imunofenotipagem e determinação do ciclo celular, e ensaios de diferenciação celular (osteogênica, condrogênica e adipogênica) para caracterização das células-tronco. Para controle de biossegurança dos cultivos, serão realizadas cariotipagem celular e hemocultura aeróbia e anaeróbia.

As populações celulares presentes nas amostras de cada paciente serão caracterizadas com os seguintes marcadores: CD29, CD44, CD73, CD90, CD105, CD166, CD146, CD14, CD19, CD45, HLA-DR, CD34, CD11B, CD31, CD106, CD4, CD5, CD9, CD10, CD13, CD36, CD38, CD45, CD49, CD56, CD133, STRO-I, OCT4, SOX-2, Nanog, SSEA-4, HLA-ABC, HLA-DR, CXCR-4, LIN, MHC-I, MHC-II, Vimentina, Actina de músculo liso, Flk-1, Sca-1, BCL, vWf.

4.7 Logística, Estrutura Física e Laboratorial, Biorrepositório

As pacientes serão provenientes do Ambulatório de Uroginecologia da UNIFESP, onde serão avaliadas clinicamente e realizarão os testes pré-tratamento. A seguir, serão encaminhadas para o Setor de Hematologia e Hemoterapia do HIAE, onde serão realizadas a biópsias de medula óssea e a coleta de sangue periférico. A extração e cultivo das células-tronco serão realizados no laboratório de terapia celular em humanos, Setor de Hematologia e Hemoterapia do HIAE. Na ocasião da terapêutica, a injeção de células-tronco autólogas será realizada nas dependências do Setor de Uroginecologia da UNIFESP.

Da mesma forma, as biópsias obtidas do musculo esquelético e do tecido adiposo na UNIFESP, Hospital São Paulo, serão encaminhadas para o laborat;orio de terapia celular da Stemcorp isolamento e cultivo das células-tronco

O transporte de material biológico entre os centros de pesquisa seguirá as normas da Anvisa com o acondicionamente adequado das amostras.

A pesquisa em questão envolve a criação de um biorrepositório, localizado no HIAE e na Stemcorp, atrelado a este projeto específico, sendo o mesmo desativado após o fim desta pesquisa. Os produtos biológicos provenientes destas amostras durante as fases do estudo e após o estudo serão automaticamente incinerados de acordo com as normas de descarte de materiais biológicos.

4.8 Banco de dados

A identificação das participantes, os dados clínicos, epidemiológicos e provenientes dos testes diagnósticos das mesmas receberão numeração sequencial. As informações serão tabuladas em banco de dados virtual de acesso aos centros de estudo, onde serão acrescidas as informações originadas das análises laboratoriais. Estas dados também serão armazenados em repositório da UNIFESP, sendo acessados e atualizados pelo centro principal.

4.9 Injeção peri-uretral do preparado celular

Seringas transparentes conterão 5 ml de solução (céulas-tronco + soro humano) a ser injetada na região da uretra média, altura do esfíncter uretral. Sob visão uretroscópica, uma agulha é introduzida pelo aparelho até o local apropriado, e introduzida aproximadamente 3 mm na mucosa. Serão injetados 2,5ml na posição 3h e

2,5ml em 9h, sem obliteração da uretra. O procedimento é ambulatorial e não haverá necessidade de anestesia ou analgesia para o procedimento.

5 Avaliação após o procedimento

A paciente será mantida em observação por 2 horas após o procedimento. Serão realizados retornos com 7, 30, 60 e 180 dias para avaliação clínica pós-tratamento.

Após 1 ano do procedimento, será realizada nova aplicação dos questionários, teste do absorvente e cistometria com avaliação de pressão de perda. Estes dados serão revelados ao fim da avaliação final de toda a população do estudo.

O desfecho primário é a melhora de qualidade de vida, que será avaliada pela avaliação do I-Qol no pré e pós-tratamento. Serão consideradas melhoradas as pacientes que tiverem escore do I-Qol de 0 (cura total) até 20 (inclusive). Pacientes com escores acima de 20 serão consideradas não melhoradas.

Além disso, serão consideradas curadas pacientes que não apresentarem perdas urinárias ao exame clínico, na cistometria, e no teste do absorvente.

6 Método Estatístico

Processamento e análise dos dados: usaremos o programa SAS versão 8.2 para a verificação de sua consistência e análise dos dados.

Análise Descritiva: a análise descritiva será utilizada para caracterizar a casuística estudada. Para as variáveis categóricas, a distribuição de freqüências absoluta e relativa será adotada e, para as variáveis contínuas, as medidas de tendência central (média e mediana) e de variabilidade (desvio padrão, limite superior e inferior) serão aplicadas.

Análise Inferencial: as comparações entre os parâmetros pré- e pós-tratamento serão analisados pelos testes de "Wilcoxon rank-sum" e "t Student" no caso de variáveis não-paramétricas e paramétricas, respectivamente. Utilizaremos o teste de freqüências do Chi-quadrado e exato de Fisher para verificar a homogeneidade entre as variáveis categóricas (raça, estado hormonal e cirurgias prévias para incontinência urinária de esforço), bem como a ausência da perda urinária no estudo urodinâmico, após a intervenção. O teste de Mann-Whitney será adotado para verificar a homogeneidade e possíveis diferenças das medianas das variáveis contínuas entre os grupos pré- e pós-tratamento que medem o sucesso

terapêutico da técnica (Questionário “I-QoL” e teste do absorvente). Em todos os testes fixar-se-á em 0,05 ou 5% (alfa menor ou igual a 0,05) o nível de rejeição da hipótese de nulidade.

7 Cronograma

A duração da pesquisa prevista é de 2 anos, seguindo o seguinte cronograma:

	**Ano 1**	**Ano 2**
Revisão da literatura	X	X
Recrutamento e seleção das participantes	X	
Avaliação clínica e realização dos testes diagnósticos pré-terapia	X	
Coleta das amostras teciduais	X	
Extração e cultivo celulares	X	
Terapia celular humana	X	
Avaliação clínica e realização dos testes diagnósticos pós-terapia		X
Compilação dos dados e análise dos resultados		X
Apresentação e divulgação dos resultados: apresentações em encontros científicos, redação e publicação de artigos em periódicos)		X

8 Avaliação e Disseminação

Os resultados obtidos serão apresentados em congressos nacionais e internacionais de Ginecologia, Uroginecologia, Biologia Celular e Molecular e Medicina Regenerativa. Temos a perspectiva de publicar os manuscritos em periódicos de fator de impacto superior a 3.0.

9 Outros Apoios

Este projeto de estudo recebe apoio parcial da FAPESP.

10 Bibliografia

1. Haylen BT, Ridder D, Freeman RM, Swift SE, Berghmans B, Lee J et al. An International Urogynecological Association (IUGA)/International Continence Society (ICS) Joint Report on the Terminology for Female Pelvic Floor Dysfunction. Neurourol Urodyn 2010;29:4–20
2. Diokno AC, Brock BM, Brown HB, Herzog AR. Prevalence of urinary incontinence and other urologic symptoms in the non-institutionalized elderly. J Urol 1986;136:1022-5
3. Rortveit G, Daltveit AK, Hannestad YS, Hunskaar S. Norwegian EPINCONT Study. Urinary incontinence after vaginal delivery or cesarean section. N Engl J Med 2003;6: 348:900-7
4. DeLancey JO. Structural support of the urethra as it relates to stress urinary incontinence: the hammock hypothesis. Am J Obst Gynecol 1994;170:1713-23
5. Liapis A, Bakas P, Pafiti A, Hassiakos D, Frangos-Plemenos M, Creatsas G. Changes in the quantity of collagen type I in women with genuine stress incontinence. Urol Res 2000;28(5):323-6
6. Liapis A, Bakas P, Pafiti A, Frangos-Plemenos M, Arnoyannaki N, Creatsas G. Changes of collagen type III in female patients with genuine stress incontinence and pelvic floor prolapse. Eur J Obstet Gynecol Reprod Biol 2001;97(1):76-9
7. Goepel C, Hefler L, Methfessel HD, Koelbl H. Periurethral connective tissue status of postmenopausal women with genital prolapse with and without stress incontinence. Acta Obstet Gynecol Scand 2003;82(7):659-64
8. Hale DS, Benson JT, Brubaker L, Heidkamp MC, Russell B. Histologic analysis of needle biopsy of urethral sphincter from women with normal and stress incontinence with comparison of electromyographic findings. Am J Obstet Gynecol 1999;180(2 Pt 1):342-8
9. Smith AR, Hosker GL, Warrell DW. The role of pudendal nerve damage in the aetiology of genuine stress incontinence in women. Br J Obstet Gynaecol 1989;96(1):29-32

10. Rud T, Andersson KE, Asmussen M, Hunting A, Ulmsten U. Factors maintaining the intraurethral pressure in women. Invest Urol 1980;17(4):343-7
11. Mason C, Dunnill P. A brief definition of regenerative medicine. Regen Med 2008;3(1):1-5
12. Ramalho-Santos M, Yoon S, Matsuzaki Y, Mulligan RC, Melton DA. "Stemness": transcriptional profiling of embryonic and adult stem cells. Science. 2002; 18;298(5593):597-600
13. Hart ML, Neumayer KM, Vaegler M, Daum L, Amend B, Sievert KD, Di Giovanni S, Kraushaar U, Guenther E, Stenzl A, Aicher WK. Cell-based therapy for the deficient urinary sphincter. Curr Urol Rep 2013;14(5):476-87
14. Williams JT. Cells isolated from adult human skeletal muscle capable of differentiating into multiple mesodermal phenotypes. Am Surg 1999; 65: 22–26
15. Chermansky CJ, Tarin T, Kwon DD, Jankowski RJ, Cannon TW, de Groat WC, Huard J, Chancellor MB. Intraurethral muscle-derived cell injections increase leak point pressure in a rat model of intrinsic sphincter deficiency. Urology 2004; 63:780–785
16. Badra S, Andersson KE, Dean A, Mourad S, Williams JK. Long-term structural and functional effects of autologous muscle precursor cell therapy in a nonhuman primate model of urinary sphincter deficiency. J Urol 2013;190(5):1938-45
17. Carr LK, Robert M, Kultgen PL, Herschorn S, Birch C, Murphy M, Chancellor MB. Autologous muscle derived cell therapy for stress urinary incontinence: a prospective, dose ranging study. J Urol 2013;189(2):595-601
18. Peters KM, Dmochowski RR, Carr LK, Robert M, Kaufman MR, Sirls LT et al. Autologous Muscle Derived Cells for Treatment of Stress Urinary Incontinence in Women. J Urol 2014;pii: S0022-5347(14)00302-4
19. Surcel C, Savu C, Chibelean C, Iordache A, Mirvald C, Sinescu I. Comparative analysis of different surgical procedures for female stress urinary incontinence. Is stem cell implantation the future? Rom J Morphol Embryol 2012;53(1):151-4
20. Roche R, Festy F, Fritel X. Stem cells for stress urinary incontinence: the adipose promise. 2010; J Cell Mol Med. 14 (1-2): 135-42.

21. Zuk PA, Zhu M, Mizuno H, Huang J, Futrell JW, Katz AJ . Multilineage cells from human adipose tissue: implications for cell based therapies. Tissue Eng. 2001; 7: 211-28.
22. Jack GS, Zhang R, Lee M. Urinary bladder smooth muscle engineered from adipose stem cell and a three dimensional synthetic composite. Biomaterials. 2009; 30: 3259-70.
23. Zeng X, Jack GS, Zhang R, et al. Treatment of SUI using adipose derived stem cells: restoration of urethral function. J Urol. 2006; 175:291.
24. Yamamoto T, Gotoh M, Hattori R, Toriyama K, Kamei Y, Iwaguro H, et al. Periurethral injection of autologous adipose derived stem cells for the treatment of stress urinary incontinence in patients undergoing radical prostatectomy: Report of two initial cases. Int J Urol. 2010; 17:75–82.
25. Becker AJ, McCullough EA, Till JE. Cytological demonstration of the clonal nature of spleen colonies derived from transplanted mouse narrow cells. Nature. 1963; 197: 452-4.
26. Friedenstein AJ, Chailakhjan RK, Lalykina KS. The development of fibroblast colonies in monolayer cultures of guinea-pig bone marrow and spleen cells. Cell Tissue Kinet. 1970; 3: 393-403.
27. Owen M. Marrow derived stromal stem cells. J Cell Science Supp. 1988; 10: 63-76.
28. Chung SY, Krivorov NP, Rausei V, Thomas L, Frantzen M, Landsittel D. Bladder reconstitution with bone marrow derived stem cells seeded on small intestinal submucosa improves morphological and molecular composition. J Urol. 2005; 174:353–359.
29. Zhang Y, Lin HK, Frimberger D, Epstein RB, Kropp BP. Growth of bone marrow stromal cells on small intestinal submucosa: an alternative cell source for tissue engineered bladder. BJU Int. 2005; 96:1120–1125
30. Zhang Y, Frimberger D, Cheng EY, Lin HK, Kropp BP. Challenges in a larger bladder replacement with cell-seeed and unseeded small intestinal submucosa grafts in a subtotal cystectomy model. BJU Int. 2006; 98:1100–1105.
31. Shukla D, Box GN, Edwards EA, Tyson DR. Bone marrow stem cells for urologic tissue engineering. World J Urol. 2008; 26:341–349.

32. Kinebuchi Y, Aizawa N, Imamura T, Ishizuka O, Igawa Y, Nishizawa O. Autologous bone marrow derived mesenchymal stem cell transplantation into injured rat urethral sphincter. Int J Urol. 2010; 17(4): 359-68.
33. Lose G, Rosenkilde P, Gammelgaard J, Schroeder T. Pad-weighing test performed with standardized bladder volume. Urology 1988;32(1):78-80
34. Patrick DL, Martin ML, Bushnell DM, Marquis P, Andrejasich CM, Buesching DP. Cultural adaptation of a quality-of-life measure for urinary incontinence. Eur Urol 1999;36(5):427-35
