# Supplementary material for: Stem cell therapy for female stress urinary incontinence: Results, limitations and lessons learned from a pilot clinical study
Source: PLoS One. 2026 Feb 27;21(2):e0342452. doi: 10.1371/journal.pone.0342452 (PMC12948050; doi:10.1371/journal.pone.0342452)
Supplement: S1 Appendix — (ZIP) [file pone.0342452.s004.zip › Supporting Information Files/StaMarcelina_PB_PARECER_CONSUBSTANCIADO_CEP_4400583_Ocultado.pdf]

CASA DE SAÚDE SANTA MARCELINA

[redacted]

PARECER CONSUBSTANCIADO DO CEP

Elaborado pela Instituição Coparticipante

DADOS DO PROJETO DE PESQUISA

Título da Pesquisa: Uso de Células-Tronco Adultas no tratamento de mulheres com incontinência urinária de esforço.

Pesquisador: RODRIGO CERQUEIRA DE SOUZA

Área Temática:

Versão: 1

CAAE: 18150613.7.3002.0066

Instituição Proponente: Casa de Saúde Santa Marcelina

Patrocinador Principal: FUNDACAO DE AMPARO A PESQUISA DO ESTADO DE SAO PAULO

DADOS DO PARECER

Número do Parecer: 4.400.583

Apresentação do Projeto:

O Assistente de Pesquisa submeteu os documentos abaixo, para analise deste comitê;

DiarioMiccional_CT_V5.docx
Projeto_CT_Plataforma_V5_Jan2020.docx
projeto_CT_Plataforma_V5_Jan2020.pdf

Objetivo da Pesquisa:

Não se aplica

Avaliação dos Riscos e Benefícios:

Não se aplica

Comentários e Considerações sobre a Pesquisa:

Não se aplica

Considerações sobre os Termos de apresentação obrigatória:

Não se aplica

Recomendações:

Não se aplica

Conclusões ou Pendências e Lista de Inadequações:

Não se aplica

Considerações Finais a critério do CEP:

Este parecer foi elaborado baseado nos documentos abaixo relacionados:

Tipo Documento	Arquivo	Postagem	Autor	Situação
Outros	DiarioMiccional_CT_V5.docx	12/01/2020 15:33:57	Maria Augusta Tezelli Bortolini	Aceito
Projeto Detalhado / Brochura Investigador	Projeto_CT_Plataforma_V5_Jan2020.docx	12/01/2020 15:32:48	Maria Augusta Tezelli Bortolini	Aceito
Projeto Detalhado / Brochura Investigador	projeto_CT_Plataforma_V5_Jan2020.pdf	12/01/2020 15:32:00	Maria Augusta Tezelli Bortolini	Aceito
TCLE / Termos de Assentimento / Justificativa de Ausência	TCLE_PF_CT_v4_revisado_Fev2019.docx	06/03/2019 22:44:48	Maria Augusta Tezelli Bortolini	Aceito
Outros	DiarioMiccional_CT.pdf	06/03/2019 22:43:53	Maria Augusta Tezelli Bortolini	Aceito
TCLE / Termos de Assentimento / Justificativa de Ausência	TCLE_PF_CT_v4_revisado_Fev2019.pdf	06/03/2019 22:42:03	Maria Augusta Tezelli Bortolini	Aceito
TCLE / Termos de Assentimento / Justificativa de Ausência	TCLE.pdf	06/12/2018 18:38:35	Maria Augusta Tezelli Bortolini	Aceito
Outros	Coep_CT.pdf	19/01/2017 13:16:54	Maria Augusta Tezelli Bortolini	Aceito
Projeto Detalhado / Brochura Investigador	351lula_tronco_humanos_Castro_v4.pdf	13/10/2016 10:35:55	Maria Augusta Tezelli Bortolini	Aceito
Declaração do Patrocinador	comprovante_Fapesp.pdf	11/10/2016 15:59:45	Maria Augusta Tezelli Bortolini	Aceito
TCLE / Termos de Assentimento / Justificativa de Ausência	TCLE_PF_CT_v4.pdf	11/10/2016 15:56:25	Maria Augusta Tezelli Bortolini	Aceito
Outros	Documento_RodrigoCastro2.jpg	24/05/2016 15:07:45	RODRIGO CERQUEIRA DE SOUZA	Aceito
Outros	Documento_RodrigoCastro1.jpg	24/05/2016 15:07:13	RODRIGO CERQUEIRA DE	Aceito

Outros	Documento_RodrigoCastro1.jpg	24/05/2016 15:07:13	SOUZA	Aceito

Situação do Parecer:
Aprovado

Necessita Apreciação da CONEP:
Não

SAO PAULO, 16 de Novembro de 2020

Assinado por:
Belmiro José Matos
(Coordenador(a))
