# Supplementary material for: Stem cell therapy for female stress urinary incontinence: Results, limitations and lessons learned from a pilot clinical study
Source: PLoS One. 2026 Feb 27;21(2):e0342452. doi: 10.1371/journal.pone.0342452 (PMC12948050; doi:10.1371/journal.pone.0342452)
Supplement: S1 Appendix — (ZIP) [file pone.0342452.s004.zip › Supporting Information Files/UNIFESP_PB_PARECER_CONSUBSTANCIADO_CEP_707060_Ocultado.pdf]

UNIVERSIDADE FEDERAL DE SÃO PAULO - UNIFESP/ HOSPITAL SÃO PAULO

[redacted]

PARECER CONSUBSTANCIADO DO CEP

DADOS DO PROJETO DE PESQUISA

Título da Pesquisa: Uso de Células-Tronco Adultas no tratamento de mulheres com incontinência urinária de esforço.

Pesquisador: RODRIGO CERQUEIRA DE SOUZA

Área Temática: Novos procedimentos terapêuticos invasivos;

Versão: 2

CAAE: 18150613.7.0000.5505

Instituição Proponente: Universidade Federal de São Paulo

Patrocinador Principal: Financiamento Próprio

DADOS DO PARECER

Número do Parecer: 707.060

Data da Relatoria: 25/06/2014

Apresentação do Projeto:

CONFORME PARECER CONSUBSTANCIADO CEP ORIGINAL Nº 401.895 de 20/9/2013 E PARECER CONSUBSTANCIADO CEP nº 655.877 de 21/05/2014

Objetivo da Pesquisa:

CONFORME PARECER CONSUBSTANCIADO CEP ORIGINAL Nº 401.895 de 20/9/2013 E PARECER CONSUBSTANCIADO CEP nº 655.877 de 21/05/2014

Avaliação dos Riscos e Benefícios:

CONFORME PARECER CONSUBSTANCIADO CEP ORIGINAL Nº 401.895 de 20/9/2013 E PARECER CONSUBSTANCIADO CEP nº 655.877 de 21/05/2014

Comentários e Considerações sobre a Pesquisa:

CONFORME PARECER CONSUBSTANCIADO CEP ORIGINAL Nº 401.895 de 20/9/2013 E PARECER CONSUBSTANCIADO CEP nº 655.877 de 21/05/2014

Considerações sobre os Termos de apresentação obrigatória:

TRATA-SE DE RESPOSTAS DE PENDÊNCIAS ENVIADOS NOS PARECERES CONSUBSTANCIADO CEP ORIGINAL Nº 401.895 de 20/9/2013 E PARECER CONSUBSTANCIADO CEP nº 655.877 de 21/05/2014

[redacted]

Recomendações:

NADA CONSTA

Conclusões ou Pendências e Lista de Inadequações:

Pendencias apontadas no parecer inicial:

Trata-se de estudo que visa aplicação de técnicas de produção de células tronco para injeção uretral objetivando melhoria da função do esfíncter uretral e melhora da incontinência urinária de esforço. A apresentação metodológica do trabalho não está clara e isto acaba impactando no julgamento ético do trabalho:

1. Na apresentação informações básicas do projeto (pdf) observa-se uma longa introdução sobre a evolução do uso de células-tronco mas na metodologia observa-se apenas o método de randomização e nenhuma informação sobre a pesquisa e impacto dela para os pacientes.
RESPOSTA: A descrição da metodologia foi refeita atendendo as recomendações feitas pela relatoria - PENDENCIA ATENDIDA

2. O projeto completo está mais detalhado mas não está clara a sequência de eventos, desde a biópsia em medula ou muscular, quando uma quanto outra, o que pode acontecer caso a cultura de células não seja positiva, nem se este método já tem resultado clínico relevante e de que ordem, uma vez que o paciente precisa saber para decidir sua participação. Nào está clara como será a aplicação das células: injeção na luz uretra ou na submucosa com agulha.
RESPOSTA: A descrição da metodologia foi refeita atendendo as recomendações feitas pela relatoria - PENDENCIA ATENDIDA

3. Fala-se em estudar 4 grupos, mas anteriormente em tipo de estudo, 6 grupos são apresentados O protocolo informa que o tratamento convencional seria cirurgia ou fisioterapia mas no grupo A 5 pacientes com incontinência urinária de esforço receberão injeção de 10ml de meio de cultura; e no Grupo B 5 plasma enriquecido com plaquetas; Apenas os outros grupos (C, D, E e F) receberão o tratamento proposto. Esses grupos são os controles? o que ocorrerá com eles após o estudo? As pacientes serão alocadas por sorteio nos grupos propostos, mas o TCLE não menciona que elas poderão ser sorteadas e serem incluidas nos grupos A e B onde NÃO SE ESTARÁ TESTANDO ESSE NOVO PROCEDIMENTO.

Endereço: Rua Botucatu, 572 1º Andar Conj. 14
Bairro: VILA CLEMENTINO **CEP:** 04.023-061
UF: SP **Município:** SAO PAULO
Telefone: (11)5539-7162 **Fax:** (11)5571-1062 **E-mail:** cepunifesp@unifesp.br

RESPOSTA: o TCLE foi refeito adequando-se aos questionamentos feitos -PENDENCIA ATENDIDA

Após revisão da literatura, decidimos por retirar os grupos com soro fisiológico e meio de cultura, mantivemos apenas os que contêm células-tronco e soro rico em plaquetas. A literatura é clara no uso terapêutico tanto das células-tronco quanto do plasma rico em plaquetas na medicina regenerativa, não havendo assim justificativa para placebos.

4. O TCLE é esquemático e não deixa claro a sequência clara dos eventos, que alguns grupos serão placebo e se submeterão à coleta de biópsias, riscos claros da biópsia óssea, riscos da cultura não ser bem sucedida e ter que ser feito novo procedimento ou ser abortado.
resposta: TCLE reapresentado atendendo as solicitações feitas. -PENDENCIA ATENDIDA

5. Sobre o financiamento: não há declaração de qualquer agente apoiador ou financiador e o custo é cerca de R$. 300.000,00. Apresentar esclarecimentos sobre esse financiamento.

resposta: quanto ao financiamento, em princípio serão utilizados recursos do próprio Departamento de Ginecologia, mas será solicitado fomento para a Fapesp. PENDENCIA ATENDIDA

6. Informa-se que o estudo será realizado na UNIFESP - Setor de Uroginecologia e Cirurgia Vaginal do Departamento de Ginecologia da UNIFESP-EPM. O CEP solicita carta do chefe do departamento dando ciência OU carta da Coordenadoria de Ensino e Pesquisa do HU/HSP dando ciência a esses procedimentos.
Em resumo, trata-se de assunto novo, sem comprovação clínica de eficácia e que apresenta na apresentação do estudo e TCLE uma simplificação exagerada, sem deixar o paciente suficientemente informado sobre o estudo e grupos que irão ser estudados. TCLE ADEQUADO
RESPONDER PONTUALMENTE CADA QUESTÃO E APRESENTAR NOVO TCLE COM AS MODIFICAÇÕES SOLICITADAS
RESPOSTA: Nova versão de TCLE apresentada - PENDENCIA ATENDIDA

[redacted]

projeto aprovado - pendências atendidas

Situação do Parecer:

Aprovado

Necessita Apreciação da CONEP:

Sim

Considerações Finais a critério do CEP:

O CEP informa que a partir desta data de aprovação, é necessário o envio de relatórios parciais (anualmente), e o relatório final, quando do término do estudo.

O presente projeto, seguiu nesta data para análise da CONEP e só tem o seu início autorizado após a aprovação pela mesma.

SAO PAULO, 02 de Julho de 2014

Assinado por:
José Osmar Medina Pestana
(Coordenador)

Endereço: Rua Botucatu, 572 1º Andar Conj. 14
Bairro: VILA CLEMENTINO **CEP:** 04.023-061
UF: SP **Município:** SAO PAULO
Telefone: (11)5539-7162 **Fax:** (11)5571-1062 **E-mail:** cepunifesp@unifesp.br
